# Mono- and Dimeric Xanthones with Anti-Glioma and Anti-Inflammatory Activities from the Ascidian-Derived Fungus *Diaporthe* sp. SYSU-MS4722

**DOI:** 10.3390/md20010051

**Published:** 2022-01-05

**Authors:** Senhua Chen, Heng Guo, Minghua Jiang, Qilin Wu, Jing Li, Hongjie Shen, Lan Liu

**Affiliations:** 1 School of Marine Sciences, Sun Yat-sen University, Zhuhai 519082, China; chensenh@mail.sysu.edu.cn (S.C.); guoh59@mail2.sysu.edu.cn (H.G.); jiangmh23@mail2.sysu.edu.cn (M.J.); wuqlin3@mail2.sysu.edu.cn (Q.W.); lijing356@mail.sysu.edu.cn (J.L.); 2 Southern Laboratory of Ocean Science and Engineering, Zhuhai 519082, China; 3 Guangdong Provincial Key Laboratory of Marine Resources and Coastal Engineering, Zhuhai 519082, China; 4 Pearl River Estuary Marine Ecosystem Research Station, Ministry of Education, Zhuhai 519082, China

**Keywords:** xanthones, ascidian-derived fungus, *Diaporthe* sp., anti-glioma activity

## Abstract

Seven new xanthones, diaporthones A−G (**1**−**7**), together with 13 known analogues, including five mono- (**8**−**14**) and six dimeric xanthones (**15**−**20**), were obtained from the ascidian-derived fungus *Diaporthe* sp. SYSU-MS4722. Their planar structures were established by extensive spectroscopic analyses, including 1D and 2D NMR and high-resolution mass spectrometry (HR-ESIMS). The absolute configurations of **1**−**7** were clearly identified by X-ray crystallographic analysis and calculation of the ECD Spectra. Compounds **15**−**20** showed significant anti-inflammatory activity with IC_50_ values between 6.3 and 8.0 μM. In addition, dimeric xanthones (**15**−**20**) showed selective cytotoxicity against T98G cell lines with IC_50_ values ranging from 19.5 to 78.0 μM.

## 1. Introduction

Glioma is a fatal disease of the central nervous system having an incidence rate of 3.20 per 100,000 people [1,2,3]. Due to limitations of the blood–brain barrier, only four drugs, including lomustine, carmustine, temozolomide, and bevacizumab, have been approved by FDA to treat glioma in the past four decades [4,5]. Temozolomide (TMZ) as the first-line therapy drug is used for the treatment of glioma; however, at least 50% of TMZ-treated patients and more than 90% of recurrent gliomas show no response to TMZ [6,7]. Thus, it is an urgent need to find more anti-glioma agents for the treatment of glioma. In recent years, anti-glioma molecules of marine origin have attracted many scientific research institutes or pharmaceutical companies’ attention. For example, marizomib (salinosporamide A) received orphan drug designation for glioblastoma in the United States [8,9], which was discovered from the marine actinomycete *Salinispora tropica* [10] and *Salinispora Arenicola* [11] and is an irreversible proteasome inhibitor with a nanomolar range IC_50_ value [11]. Depatuxizumab vedotin was an antibody–drug conjugate (ADC) in phase 3 trials to treat newly diagnosed glioblastoma with EGFR amplification [8], which was developed from pentapeptide dolastatin 10 that was produced from the sea hare *Dolabella Auricularia* [12].

**Figure 1:**
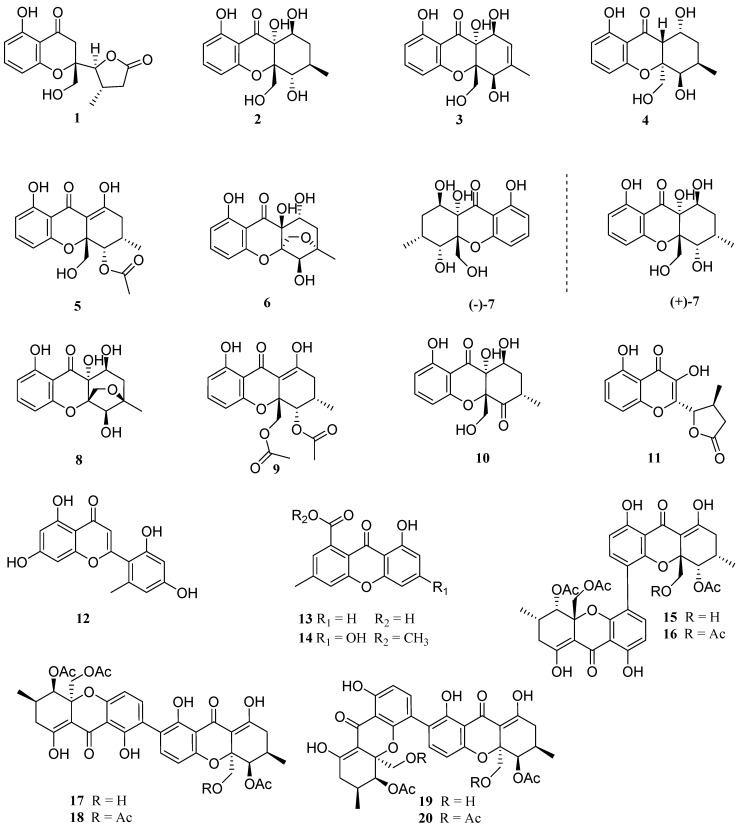
Chemical structures of compounds **1**–**20**.

In the past five years, our research group has focused on the bioactive secondary metabolites of ascidian-derived fungi collected from the South China Sea [5,13,14,15,16]. A marine-derived fungus *Diaporthe* sp. SYSU-MS4722 was isolated from the South China Sea, whose EtOAc extract of a fermentation broth showed moderate anti-glioma activity. A subsequent chemical investigation led to the isolation of seven new polyketides, diaporthones A−G (**1**−**7**), together with 13 known analogues, including five mono- (**8**−**14**) and six dimeric xanthones (**15**−**20**) (Figure 1). Dimeric xanthones (**15**−**20**) revealed selective cytotoxicity against T98G cell lines with IC_50_ values ranging from 19.5 to 78.0 μM.

## 2. Results and Discussion

The EtOAc extract of marine-derived fungus *Diaporthe* sp. SYSU-MS4722 was performed on the repeated silica gel and Sephadex LH-20 column chromatography, followed by semipreparative HPLC to afford seven new xanthones, diaporthones A−H (**1**−**7**) along with 13 known analogues including seven mono- (**8**−**14**) and six dimeric xanthones (**15**−**20**).

Diaporthone A (**1**) was obtained as yellow crystal, and its molecular formula was established as C_15_H_16_O_6_ by the negative HRESIMS ions at *m*/*z* 291.0869 [M − H]^−^ (calculated for C_15_H_15_O_6_, 291.0874) (Figure S1), indicating eight degrees of unsaturation. The ^1^H NMR (Table 1) displayed three aromatic protons (*δ*_H_ 6.40 (1H, d, *J* = 8.3 Hz); 6.43 (H, *J* = 8.3 Hz); 7.38 (1H, t, *J* = 8.3 Hz)) owing to a 1,2,3-trisubstituted benzene ring, two methines (δ_H_ 4.39 (1H, d, *J* = 4.2 Hz); 2.85 (H, m)), two methylenes (*δ*_H_ 2.22 (1H, dd, *J* = 8.9, 21.6 Hz), 2.86 (1H, m); 3.76 (2H, s), *δ*_H_ 2.98 (2H, br s)), and one methyl (*δ*_H_ 1.20 (3H, d, *J* = 6.6 Hz)). The ^13^C NMR showed the presence of 15 carbons, which were assigned with the help of an HMBC experiment. Except for signals (δ_C_ 161.4, 159.4, 138.1, 108.7, 107.2, 106.9) attributed to one aromatic ring, the other carbons were identified as one conjugated carbonyl (*δ*_C_ 196.8), one ester carbonyl (*δ*_C_ 177.3), and remaining six sp^3^ hybridized carbons. ^1^H and ^13^C NMR data (Table 1) suggested that **1** belonged to a chromone derivative containing a *γ*-lactone moiety.

**Figure 2:**
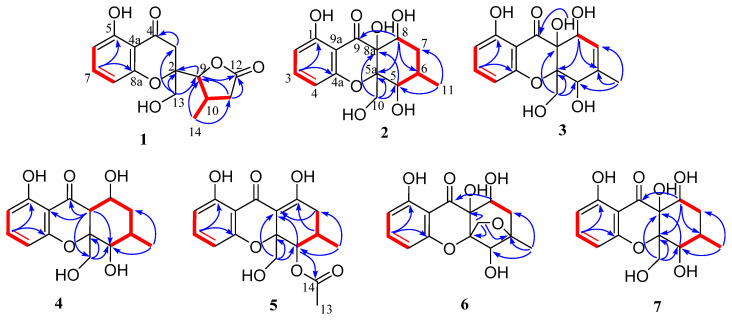
Key ^1^H-^1^H COSY (red line) and HMBC (blue arrow) correlations of compounds **1**–**7**.

The planar structure of **1** was identified based on the extensive 2D NMR (^1^H–^1^H COSY, HSQC, and HMBC) (Figures S2–S7) spectroscopic data (Figure 2). The HMBC correlations from H-7 to C-5 and C-8a, from H-8 to C-4a, from H-13 to C-2 and C-3, and ^1^H–^1^H COSY of H-6/H-7/H-8, as well as the NMR chemical shifts, complete a 4-hydroxylchromone skeleton with a hydroxymethyl group at C-2. The γ-lactone moiety with a methyl group was assigned by HMBC correlations from H-9 to ester carbonyl C-12, from H-11 to C-9 and C-12, from H-14 to C-9, C-10 and C-11, and the ^1^H–^1^H COSY correlations of H-9/H-10/H-11 and H-10/H-14. The *γ*-lactone moiety was finally linked to C-2 of the chromone skeleton, supported by HMBC correlations from H-13 to C-9 and H-9 to C-2.

**Figure 3:**
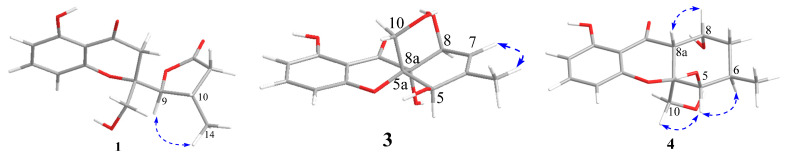
Key NOE (blue dash arrow) correlations of compounds **1**, **3**, and **4** (3D structures were generated by minimizing the energy using a molecular mechanics (MM2) computation by Chem3D).

**Figure 4:**
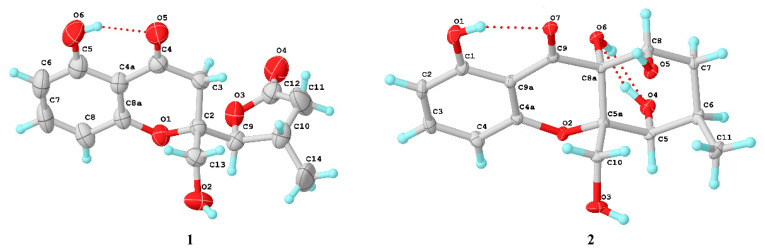
X-ray crystallographic analysis of **1** and **2**.

**Figure 5:**
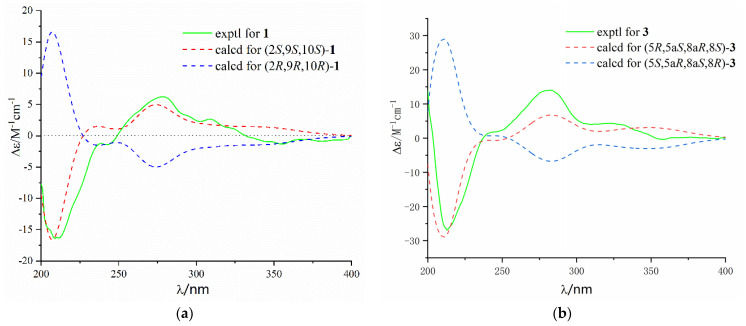
Experimental and predicted ECD spectra of **1** (**a**) and **3** (**b**) in MeOH.

The NOESY spectrum of **1** showed a correlation from H-9 to H-14, indicating that H-9 and H-14 were on the same side of the *γ*-lactone (Figure 3). The X-ray structure (Figure 4) showed that the protons H-13 and H-9 were located on the close position of the axis of rotation C2–C9, indicating that the relative configuration of **1** was 2S*, 9S*, and 10S*. In addition, the ECD spectrum of (2*S*, 9*S*, 10*S*)-**1** calculated by a quantum chemical method at the [B3LYP/6-311 + G(2d,p)] was in good agreement with that of the experimental one (Figure 5). Thus, the absolute configuration of **1** was identified as 2*S*, 9*S*, and 10*S*.

It can be seen that the proton H_2_-3 of **1** was the α-H atom of ketone with weak acid, whose α-hydrogen exchange (α-deuterodeprotonatioion reaction) could be found in CD_3_OD. The signal of H_2_-3 was observed as a weak and small peak, and the integrals were much less than two in the ^1^H NMR spectrum. The natural product with the α-H atom of ketone moiety would be better for acquiring the NMR data under the deuterated solvents without exchangeable deuterium.

Diaporthone B (**2**) was obtained as yellow crystal, and its molecular formula was established as C_15_H_18_O_7_ by the negative HRESIMS ions at *m*/*z* 309.0976 [M − H]^−^ (calculated for C_15_H_17_O_7_, 309.0980), indicating eight degrees of unsaturation. The ^1^H NMR (Figure S10) displayed three aromatic protons (*δ*_H_ 6.44 (1H, dd, *J* = 8.2, 0.9 Hz); 6.55 (H, d, *J* = 8.2 Hz); 7.39 (1H, t, *J* = 8.3 Hz)), three methines (*δ*_H_ 4.44 (1H, t, *J* = 4.2 Hz); 4.29 (1H, m); 2.34 (1H, m)), two methylenes (*δ*_H_ 1.57 (1H, dd, *J* = 14.5, 2.8 Hz), 2.43 (1H, m); 3.83 (1H, d, *J* = 13.5 Hz), 4.28 (1H, s)), and one methyl (*δ*_H_ 1.32 (3H, d, *J* = 7.8 Hz)). The ^13^C NMR showed the presence of 15 carbons corresponding to seven sp^2^ hybrid carbons including one ketone carbonyl (*δ*_C_ 196.4) for xanthone characteristic and eight sp^3^ hybrid carbons. The planar structure of **2** was identified by ^1^H–^1^H COSY, HSQC, and HMBC spectroscopic data (Figure 2). The HMBC correlations from H-10 to C-5a and C-8a, from H-3 to C-1 and C-4a, and ^1^H–^1^H COSY of H-2/H-3/H-4, as well as the NMR chemical shifts, suggested the presence of a hydroxylchromone skeleton with a hydroxymethyl group at C-5a and a hydroxyl at C-8a. The remaining ring was assigned to be 4-hydroxyl-5-methyl cyclohexenol on the basis of ^1^H–^1^H COSY of H-6/H-7/H-8/H-9 and H-6/H-11, and key HMBC correlations from H-8 to C-9 and C-5a, H-5 to C-8a. Finally, the structure and absolute configuration (5*S*, 5a*S*, 6*R*, 8a*R*, 8*S*) were distinctly demonstrated by X-ray crystallographic analysis (Figure 4) from Cu Kα data with a Flack parameter of −0.01(14) [17] and a Hooft parameter of 0.06(7) [18].

Diaporthone C (**3**) was obtained as a colorless oil, whose molecular formula was determined as C_15_H_16_O_7_ on the basis of negative-ion HRESIMS (*m*/*z* 307.0820 [M − H]^−^, calculated for C_15_H_15_O_7_, 307.0823). Detailed analysis of its NMR spectroscopic data (Table 2) suggested that **3** was similar to **2** belonging to the xanthone class, except for the presence of an additional double bond (*δ*_C_ 140.1 and 122.1; *δ*_H_ 5.60) in **3**. The key HMBC correlations from methyl protons H-11 to olefinic carbons C-6 and C-7 were allowed to assign the location of the double bond in **3**. The gross structure of **3** was identified by the 2D NMR spectroscopy (Figure 2). NOE correlations of H-5 with H-10 and H-10 with H-8 were not observed, and the relative configuration of compound **3** should be 5R*, 5aS*, 8aR*, and 8S* (Figure 3). The predicted ECD curve of **3** matched well with the experimental one (Figure 5). Hence, the absolute configuration of **3** was identified as 5*R*, 5a*S*, 8a*R*, and *8S*.

**Figure 6:**
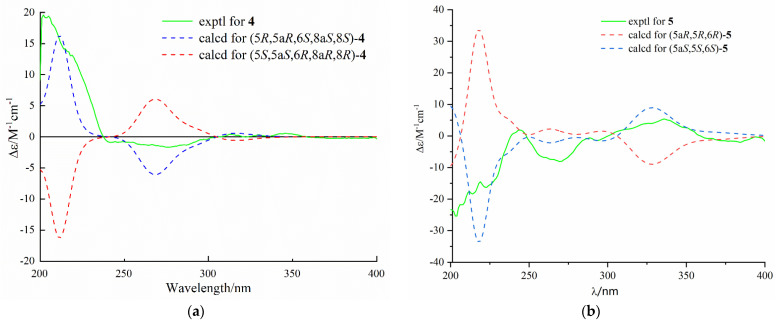
Experimental and predicted ECD spectra of **4** (**a**) and **5** (**b**) in MeOH.

Diaporthone D (**4**) was obtained as a colorless oil, and its molecular formula C_15_H_19_O_6_ was identified by the positive HRESIMS ion at HRESIMS *m*/*z* 295.1169 [M + H]^+^ (calculated for C_15_H_19_O_6_, 295.1176). Comparison of the ^1^H and ^13^C NMR data (Table 1) of **4** with those of **2** suggested that **4** possessed the same xanthone framework, except for one of the hydroxyl groups (8a-OH) in **2** being replaced by a proton in **4**. The ^1^H–^1^H COSY of H-8 and H-8a and HMBC correlations from H-8a to C-9 and C-10 assigned the position of the proton H-8, in accordance with the proton having double peaks at 2.48 ppm (Figures S26–S31). The gross structure of **4** was identified by the 2D NMR spectroscopy (Figure 2). The coupling constant value of *J*_8,8a_ = 4.5 Hz and NOE correlation of H-8a with H-8 indicated that the orientation of H-8 and H-8a was the same face of cyclohexane (Figure 3). The coupling constant value of *J*_5,6_ = 2.1 Hz and NOE correlation of H-5 with H-6 and H-10 suggested that H-5, H-6, and H-10 were on the same side. The relative configuration of compound **4** was assigned as 5R*, 5aR*, 6R*, 8aR*, and 8R*. The absolute configuration of **4** was assigned by comparing the experimental and calculated ECD spectra, and the calculated ECD spectrum was agreed with that of the experimental one (Figure 6). Therefore, the absolute configuration of **4** was assigned as 5*R*, 5a*R*, 6*R*, 8a*R*, and 8*R.*

Diaporthone E (**5**) was obtained as a colorless oil. The negative HRESIMS *m*/*z* 333.0975 [M − H]^−^ (calculated for C_17_H_17_O_7_, 333.0980) suggested the molecular formula of **5** was C_17_H_18_O_7_ with four degrees of unsaturation. The 1D and 2D NMR data (Figures S34–S39) indicated that compound **5** shared the same xanthone skeleton as penexanthone B (**9**). The only difference between them was that compound **5** was the absence of an additional acetyl group (10-Ac) compared to **6**. Compound **5** was a precursor of biosynthesis of 12-deacetylphomoxanthone A (**15**), and there is reason to believe that they share the same absolute configuration of 5*S*, 5a*S*, and 6*S*. The predicted ECD spectrum of (5*S*, 5a*S*, 6*S*)-**5** showed well fit with that of the experimental one (Figure 6). Thus, compound **5** was identified as de-10-acetylpenexanthone B.

**Figure 7:**
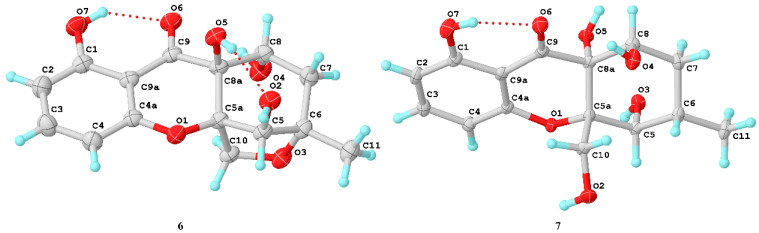
X-ray crystallographic analysis of **6** and **7**.

Diaporthone F (**6**) was obtained as a yellow crystal and had the same molecular formula (C_15_H_16_O_7_) as **8** established by the HR-ESIMS ions at *m*/*z* 307.0828 [M − H]^−^ (calculated for C_15_H_15_O_7_, 307.0823). Compound **6** shared the same planar structure as phomoxanthone G (**8**), which was further identified by ^1^H-^1^H COSY, HSQC, and HMBC spectroscopies (Figures S42–S47). Detailed analysis of their NMR (Table 3), diaporthone F (**6**) and phomoxanthone G should be a pair of epimers. The structure of **6** (Figure 7) and configuration (5*R*, 5a*R*, 6*S*, 8a*S*, 8*R*) were identified by X-ray crystallographic analysis from Cu Kα data with a Flack parameter of -0.13(15) [17] and a Hooft parameter of 0.16(9).

**Figure 8:**
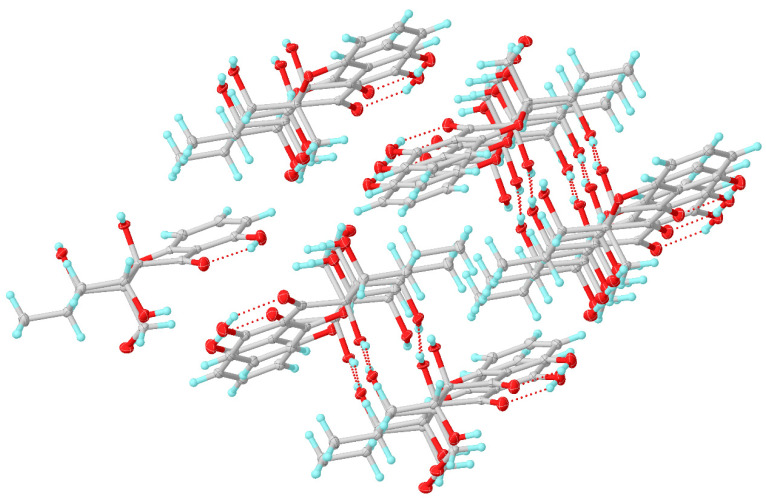
Molecular packing properties of **7**.

**Figure 9:**
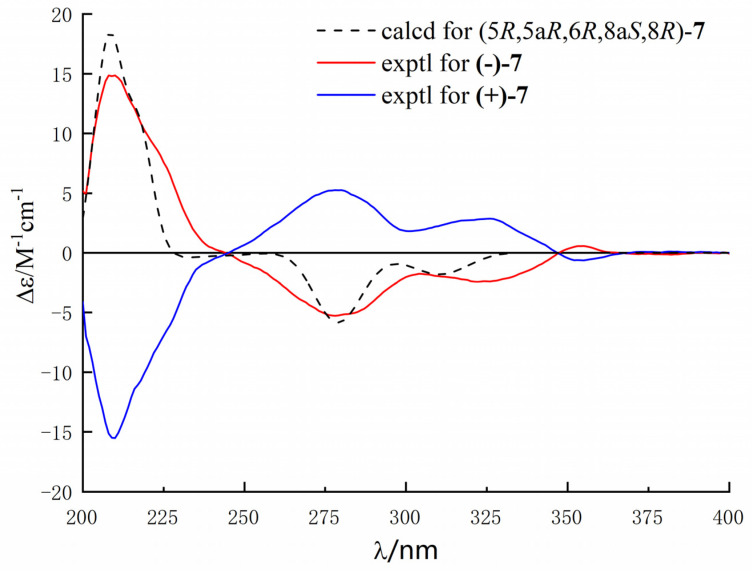
Experimental and predicted ECD spectra of (+)- and (-)-**7** (in MeOH).

Compounds **7** was obtained as a yellow crystal and had the molecular formula C_15_H_18_O_7_, which was established by the HR-ESIMS ions at *m*/*z* 309.0978 [M − H]^−^ (calculated for C_15_H_17_O_7_, 309.0979). The structure of **7** was clearly identified by X-ray crystallographic analysis (Figure 6), whose relative and planar structure was the same as that of phaseolorin D [19]. In detail, the X-ray structure was the monoclinic space group *P*2_1_/c (Figure 7), which was different from that of phaseolorin D ((+)-**7**) with the triclinic space group. It suggested that compound **7** should be an enantiomer in the solid crystals (Figure 8), which showed an excellent fit with the result of no signal of CD spectrum and no optical activity sign in methanol. Subsequently, the chiral HPLC was used for purification of (±)-**7**, and the two enantiomers, (+)-**7** (*t*_R_ = 28.7 min) and (−)-**7** (*t*_R_ = 31.5 min) were purified, respectively, and showed opposite Cotton effects in their CD spectra and opposite optical rotations (Figure 9). The calculated ECD curve of (5*R*, 5a*R*, 6*R*, 8a*S*, 8*R*)-**7** was comparable to the experimental one of (−)-**7**, resulting in that the absolute configuration of (−)-**7** was 5*R*, 5a*R*, 6*R*, 8a*S*, 8*R*. Compounds **2** and (±)-**7** were epimers derived from the same precursor, chrysophanol, and a possible biogenetic pathway for **2** and (±)-**7** was proposed, as shown in Figure S62 (supplementary mateial). Compounds **2**, **3**, and (+)-**7** showed a very similar ECD spectrum with a strong and negative Cotton effect (CE) at 209 nm and two negative CEs at approximately 281 and 326 nm (Figure S63, Supplementary Material), while (−)-**7** revealed opposite Cotton effects. The negative Cotton effect (CE) at 209 nm should be derived from the n−π* transition of the chromone chromophore with the absolute configuration of 5a*S* and 8a*R*.

The other known compounds were identified as phaseolorin D (+)-(**7**) [19], Phomoxanthone G (**8**) [20], penexanthone B (**9**) [21], phaseolorin E (**10**) [19], phomoxanthone F (**11**) [20], penimethavone A(**12**) [22], monodictyxanthone (**13**) [23], methyl 6,8-dihydroxy-3-methyl-9-oxo-9*H*-xanthene-1-carboxylate (**14**) [24], 12-deacetylphomoxanthone A (**15**) [25], phomoxanthone A (**16**) [26], dicerandrol B (**17**) [27], dicerandrol C (**18**) [27], phomoxanthone B (**19**) [26] and deacetylphomoxanthone B (**20**) [28] by comparing their spectroscopic data with those reported in the literature.

All isolates were evaluated for anti-glioma using T98G, U87MG, and U251 human cell lines with temozolomide as the positive control. Only dimeric xanthones (**15**−**20**) exhibited promising growth-inhibitory effects on the T98G cell line with IC_50_ values between 19.5 to 78.0 μM (Table 4). Additionally, all monomeric xanthones (**1**−**14**) displayed weak or no cytotoxicity against T98G, U87MG, and U251 human cell lines. The result suggested that the dimeric skeleton of xanthones played an important role in anti-glioma activity.

All compounds (**1**−**20**) were also tested for the inhibition of nitric oxide (NO) production in RAW264.7 cells activated by lipopolysaccharide. Compounds **15**−**20** showed strong inhibition of NO with IC_50_ values between 6.3 and 8.0 μM, compared to the positive control indomethacin, whose IC_50_ value was 35.8 μM (Table 4). Compounds **11** and **14** exhibited considerable anti-inflammatory activity with IC_50_ values of 41.4 and 32.2 μM, respectively. However, the other compounds do not have effects on anti-inflammatory activity (IC_50_ > 50 μM).

## 3. Materials and Methods

### 3.1. General Experimental Procedures

Optical rotations were recorded on an MCP-200 polarimeter (Anton Paar, Graz, Austria) with MeOH as solvent at 25 °C. UV spectra were measured on a Blue Star A spectrophotometer. IR data were carried out on a Fourier transformation infrared spectrometer coupled with an EQUINOX 55 infrared microscope (Bruker, Rheinstetten, Germany). A Bruker Avance 400 MHz spectrometer (Bruker, Karlsruhe, Germany) was used for 1D and 2D NMR spectra test with TMS as an internal standard. ESIMS and HRESIMS data were measured on an ACQUITY QDA (Waters Corporation, Milford, MA, USA) and an LTQ-Orbitrap LC-MS spectrometer (Thermo Corporation, Waltham, MA, USA), respectively. A Shimadzu Essentia LC-16 was used for HPLC preparative separations by a Welch-Ultimate XB-C18 column (250 × 21.2 mm, 5 μM, 12 nm, Welch Materials, Inc., Shanghai, China) an ACE-5-C18-AR, ACE-5-CN-ES and ACE-C18-PFP column (250 × 10 mm, 5 μM, 12 nm, FLM Advanced Chromatography Technologies Ltd., Guangzhou, China). Column chromatography (CC) was performed on silica gel (200−300 mesh, Qingdao Marine Chemical Inc., Qingdao, China) and Sephadex LH-20 (Amersham Biosciences, Uppsala, Sweden).

### 3.2. Fungal Material

The experimental strain SYSU-MS4722 was isolated from the ascidian *Styela plicata* that was obtained by Professor Lan Liu from the Bay of Da’ao, Shenzhen City, Guangdong, Province, China, in April 2016. The standard protocol [29] was used for the isolation of fungus. The molecular biological protocol that included DNA amplification and sequencing of the ITS region was used for fungal identification. The sequence data of the fungal strain have been deposited at GenBank with accession no. OK623372. A BLAST search result suggested that the sequence was most similar (100%) to the sequence of *Diaporthe* sp. NFIF-2-6 (compared to MW202988.1).

### 3.3. Extraction and Isolation

The strain *Diaporthe* sp. SYSU-MS4722 was fermented on a solid medium in a 1 L culture flask (containing 50 g of rice and 50 mL of H_2_O with 3% sea salt) with a total of 120 flasks incubating at room temperature for 30 days. The solid fermentation was extracted with MeOH four times to afford a crude extract, and then the crude was dissolved in H_2_O and continuously was extracted four times with EtOAc. The EtOAc extract (42 g) was subjected to a silica gel column eluting with gradient petroleum ether/EtOAc (from 8:2 to 0:1) to obtain six fractions (A–F).

Fr.A was fractionated on a Sephadex LH-20 column with MeOH/CH_2_Cl_2_ (50:50) to afford three fractions (Fr.A.1 to Fr.A.3). Fr.A.3 was further purified by RP-HPLC (MeOH/H_2_O, 80:20 flow rate 2 mL/min, ACE-C18-PFP column 10 × 250 mm, 5 μM) to give **5** (5.0 mg) and **9** (11.0 mg). Fr.B was fractionated on a Sephadex LH-20 column with CH_2_Cl_2_/MeOH to provide four subfractions (Fr.B.1 to Fr.B.4). Fr.B.1 was further subjected to silica gel chromatography eluting with CH_2_Cl_2_/MeOH (99:1) to afford **11** (2.0 mg). Fr.B.2 was further purified by silica gel chromatography and RP-HPLC (MeOH/H_2_O, 75:25 flow rate 2 mL/min, ACE-C18-PFP column 10 × 250 mm, 5 μM) to obtain **13** (6.0 mg), **16** (2.0 mg), and **19** (3.0 mg). Fr.B.3 was further fractionated on a silica gel column and RP-HPLC to give **18** (14.0 mg) and **20** (12.0 mg). Fr.B.4 was subjected to a silica gel column with CH_2_Cl_2_/MeOH (98:2) and then purified by RP-HPLC with MeOH/H_2_O (70:30) to give **1** (2 mg), **15** (5.0 mg), and **17** (7.0 mg). Fr.C was applied to a silica gel column eluting with CH_2_Cl_2_/MeOH (97:3) to afford five fractions (Fr.C.4.1 to Fr.C.4.5). Fr.C.4.2 was further purified by silica gel column to give **4** (8.0 mg). Fr.C.4.4 was subjected to silica gel chromatography to afford **8** (9.1 mg) and subfraction (Fr.C.4.4.2). Fr.C.4.4.2 was further purified by RP-HPLC (MeOH/H_2_O, 65:35 flow rate 2 mL/min, ACE-C18-PFP column 10 × 250 mm, 5 μM) to give **2** (5.0 mg, *t*_R_ = 11.2 min), **7** (2.0 mg, *t*_R_ = 12.1 min), and **10** (11.0 mg, *t*_R_ = 14.0). Fr.C.4.5 was further fractionated on a silica gel column and RP-HPLC to afford **3** (4.0 mg), **12** (10.0 mg), and **14** (9.0 mg).

**Compound 1:** C_15_H_16_O_6_; Yellow crystal; [α]20D (c 0.01, MeOH) +41.2; UV (MeOH) *λ*_max_ (log ε) 270 (3.91) nm; CD (MeOH) *λ*_max_ (Δε) 212 (−2.66), 243 (+1.49) nm; IR (neat) *v*_max_ 3458, 2976, 1786, 1653, 1468, 1234, 1065 cm^−1^; HR-ESIMS *m*/*z* 291.0869 [M − H]^−^ (calculated for C_15_H_15_O_6_, 291.0874).

**Compound 2:** C_15_H_18_O_7_; Colorless crystal; [α]20D (c 0.01, MeOH) +38.6; UV (MeOH) *λ*_max_ (log ε) 208 (4.10), 277 (3.85) nm; CD (MeOH) λ_max_ (Δε) 209 (−28.85), 281 (+10.75), 326 (+6.41) nm; IR (neat) *v*_max_ 3446, 2952, 1633, 1610, 1456, 1225, 1028 cm^−1^; HR-ESIMS *m*/*z* 309.0976 [M − H]^−^ (calculated for C_15_H_17_O_7_, 309.0980).

**Compound 3:** C_15_H_16_O_7_; Colorless crystal; [α]20D (c 0.02, MeOH) +32.4; UV (MeOH) *λ*_max_ (log ε) 205 (4.03), 278 (3.87) nm; CD (MeOH) *λ*_max_ (Δε) 212 (−17.55), 282 (+9.36), 323 (+2.89) nm; IR (neat) *v*_max_ 3402, 2962, 1641, 1583, 1468, 1213, 1043 cm^−1^; HR-ESIMS *m*/*z* 307.0820 [M − H]^−^ (calculated for C_15_H_15_O_7_, 307.0823).

**Compound 4:** C_15_H_18_O_6_; Colorless powder; [α]20D (c 0.02, MeOH) +13.4; UV (MeOH) *λ*_max_ (log ε) 207 (4.20), 276 (3.52) nm; CD (MeOH) *λ*_max_ (Δε) 202 (+24.71), 219 (+12.97) nm; IR (neat) *v*_max_ 3454, 2970, 1722, 1643, 1456, 1363, 1232, 1045 cm^−1^; HR-ESIMS *m*/*z* 295.1169 [M + H]^+^ (calculated for C_15_H_19_O_6_, 295.1176).

**Compound 5:** C_17_H_18_O_7_; Colorless powder; [α]20D (c 0.01, MeOH) +9.2; UV (MeOH) *λ*_max_ (log ε) 224 (3.75), 254(3.50) nm; CD (MeOH) *λ*_max_ (Δε) 243 (+0.44), 235 (+1.32) nm; IR (neat) *v*_max_ 3438, 2935, 2871, 1745, 1616, 1468, 1238, 1055 cm^−1^; HR-ESIMS *m*/*z* 333.0975 [M-H]^−^ (calculated for C_17_H_17_O_7_, 333.0980).

**Compound 6:** C_15_H_16_O_7_; Colorless crystal; [α]20D (c 0.02, MeOH) −175; UV (MeOH) *λ*_max_ (log ε) 208 (4.05), 278(3.78), 356 (3.30) nm; IR (neat) *v*_max_ 3367, 1630, 1459, 1222, 1033, 813 cm^−1^; HR-ESIMS *m*/*z* 307.08278 [M − H]^−^ (calculated for C_15_H_15_O_7_, 307.08233).

**Compound 7:** C_15_H_18_O_7_; Colorless crystal; UV (MeOH) *λ*_max_ (log ε) 209 (4.15), 279(3.78), 353 (3.30) nm; IR (neat) *v*_max_ 3499, 3289, 2936, 1648, 1629, 1461, 1224, 1022, 811, 694 cm^−1^; HR-ESIMS *m*/*z* 309.0978 [M − H]^−^ (calculated for C_15_H_19_O_7_, 309.0979).

(+)-(**7**): [α]20D (c 0.0035, MeOH) +73.4; ECD (MeOH) *λ*_max_ (∆ε): 209 (−16.8), 279 (+5.6), 327 (+2.8) nm.

(−)-(**7**): [α]20D (c 0.0011, MeOH) −79.2; ECD (MeOH) *λ*_max_ (∆ε): 209 (+15.4), 279 (−5.1), 327 (+2.5) nm.

### 3.4. X-ray Crystallographic Analysis

The crystals of compounds **1**, **2**, **6**, and **7** were obtained on the base of the vapor diffusion method. All single-crystal X-ray diffraction data were collected on a Rigaku Oxford diffractometer with Cu-Kα radiation (λ = 1.54178 A). The structures were solved by direct methods using SHELXS-97 and refined full-matrix least-squares difference Fourier techniques using Olex2-1.2. All hydrogen atoms bonded to carbons were added at the geometrically ideal positions by the “ride on” method. All crystallographic data of **1**, **2**, **6**, and **7** have been deposited with the Cambridge Crystallographic Data Centre. Copies of the data can be obtained, free of charge, on application to the Director, CCDC, 12 Union Road, Cambridge CB2 1EZ, UK (fax: 44-(0)1223-336033, or e-mail: deposit@ccdc.cam.ac.uk).

**Compound 1:** C_15_H_16_O_6_ (*Mr* = 291.09 g/mol), orthorhombic, space group P2_1_2_1_2_1_, *a* = 5.4585(4) Å, *b* = 11.5804(10) Å, *c* = 22.0066(14) Å, α = 90°, *β* = 90°, *γ* = 90°,*V* = 1391.07(18) Å^3^, *Z* = 4, *T* = 249.99(10) K, *µ*(Cu Kα) = 0.915 mm^−1^, *D*_calc_ = 1.3955 g/cm^3^, 5334 reflections measured, 2742 unique (*R*_int_ = 0.0538, *R*_sigma_ = 0.0628), which were used in all calculations. The final *R*_1_ was 0.0557 (*I* > = 2*u*(*I*)) and *wR*_2_ was 0.1503. The Flack parameter was 0.0(3), and the Hooft parameter was -0.2(3). Goodness of fit on F^2^ was 1.048. CCDC 2116967.

**Compound 2:** C_15_H_18_O_7_ (*Mr* = 310.31 g/mol), monoclinic, space group P2_1_, *a* = 10.1547(4) Å, *b* = 7.1459(2) Å, *c* = 10.3653(4) Å, α = 90°, *β* = 116.414(4)°, *γ* = 90°, *V* = 673.63(5) Å^3^, *Z* = 2, Crystal size 0.35 × 0.3 × 0.25 mm^3^, *T* = 150.00(10) K, *µ*(Cu Kα) = 1.036 mm^−1^, *D*_calc_ = 1.530 g/cm^3^, 4665 reflections measured, 2621 unique (*R*_int_ = 0.0268, *R*_sigma_ = 0.0298), which were used in all calculations. The final *R*_1_ was 0.0359 (I > = 2*u*(*I*)) and *wR*_2_ was 0.0986. The Flack parameter was −0.01(14), and the Hooft parameter was 0.06(7). Goodness of fit on F^2^ was 1.044. CCDC 2116970.

**Compound 6:** C_15_H_16_O_7_ (*Mr* = 308.29 g/mol): monoclinic, space group P2_1_, *a* = 8.0807(2) Å, *b* = 10.5548(2) Å, *c* = 8.3669(2) Å, α = 90°, *β* = 93.743(2)°, *γ* = 90°, *V* = 712.09(3) Å^3^, *Z* = 2, Crystal size 0.25 × 0.2 × 0.02 mm^3^, *T* = 149.98(10) K, *µ*(CuKα) = 0.055 mm^−1^, *D*_calc_ = 0.068 g/cm^3^, 6306 reflections measured, 2765 unique (*R*_int_ = 0.0384, *R*_sigma_ = 0.0323), which were used in all calculations. The final *R*_1_ was 0.0402 *(I* > 2*σ*(*I*)) and *wR*_2_ was 0.1178. The Flack parameter was 0.13(15), and the Hooft parameter was 0.16(9). Goodness of fit on *F*^2^ was 1.069. CCDC 2116974.

**Compound 7:** C_15_H_18_O_7_ (*Mr* = 310.30 g/mol): monoclinic, space group P2_1_/c, *a* = 11.3702(3) Å, b = 7.30602(17) Å, c = 16.9135(5) Å, α = 90°, *β* = 108.413(3)°, *γ* = 90°, *V* = 1333.09(7) Å^3^, *Z* = 4, Crystal size 0.3 × 0.25 × 0.08 mm^3^, *T* = 150.00(10) K, *µ*(Cu Kα) = 1.047 mm^−1^, *D*_calc_ = 1.5460 g/cm^3^, 3935 reflections measured, 2546 unique (*R*_int_ = 0.0295, *R*_sigma_ = 0.0370), which were used in all calculations. The final *R*_1_ was 0.0520 (*I* > = 2*u*(*I*)) and *ωR*_2_ was 0.1591. Goodness of fit on *F*^2^ was 1.058. CCDC 2116976.

### 3.5. Calculation of the ECD Spectra

Merck molecular force field (MMFF) and DFT/TD-DFT calculations were carried out with the Spartan’14 software package (Wavefunction Inc., Irvine, CA, USA) and the Gaussian 09 program, respectively. MMFF conformational search generated low-energy conformers within a 10 kcal·mol^−1^ energy window and optimized using PM6 semi-empirical optimizations. Then, each conformer was optimized with HF/6-31G(d) method in Gaussian09. Further optimization was performed at the b3lyp/6-311g** level. The frequency was calculated at the same level to confirm each optimized conformer with the true minimum and to estimate their relative thermal free energies (ΔG) at 298.15 K. The optimized conformers were continually used for the ECD calculations in methanol, which were carried out with Gaussian09 (b3lyp/6-311g**). Solvent effects were taken into account by using the polarizable continuum model (PCM). The ECD data were generated by the program SpecDis using a Gaussian band shape with 0.30 eV exponential half-width from dipole-length dipolar and rotational strengths, and the final ECD spectrum was drawn by Origin 2018. All calculations were performed by Tianhe-2 of the National Super Computer Center in Guangzhou.

### 3.6. Anti-Glioma Activity

The human glioma cell lines, T98G, U87MG, and U251, were purchased from the Cell Bank of the Chinese Academy of Sciences. Cells were cultured in Dulbecco’s Modified Eagle’s Medium (DMEM, Gibco, Carlsbad, CA, USA), containing 10% fetal bovine serum (FBS), 100 IU/mL penicillin, 100 µg/mL streptomycin (all from Gibco, Carlsbad, CA, USA) in a cell incubator with 5% CO_2_ at 37 °C.

Cell proliferation was analyzed by MTT according to the manufacturer’s instructions. Briefly, T98G, U87MG, and U251 were digested and seeded at 1 × 10^3^ cells/well in 96-well plates and cultured in 100 µL medium overnight. The cells were treated by tested compounds with gradient concentrations for 48 h. At each indicated time point, MTT solution (10 µL/well) was added and then incubated at 37 °C for 2 h. The optical density (OD) at 450 nm was recorded by a microplate reader (Multiskan GO, Thermo Scientific, Waltham, MA, USA). Each experiment was performed three times.

### 3.7. Anti-Inflammatory Activity

RAW 264.7 cells were seeded in 96-well plates at a density of 5 × 10^5^ cells/mL. After 12 h, the cells were treated with 1 µg/mL of LPS and tested samples, followed by additional incubation for 24 h at 37 °C. MTT stock solution (2 mg/mL) was added to wells for a total reaction volume of 100 µL. After 4 h incubation, the supernatants were aspirated. The formazan crystals in each well were dissolved in DMSO (100 µL), and the absorbance was measured with the wavelength of 490 nm by a microplate reader (Multiskan GO, Thermo Scientific). The data were expressed as mean percentages of the viable cells compared to the respective control. After pre-incubation of RAW 264.7 cells (1.5 × 10^5^ cells/mL) with 1 µg/mL LPS and samples at 37 °C for 24 h, the quantity of nitrite accumulated in the culture medium was measured as an indicator of NO production. Briefly, cell culture medium (50 µL) was added with Griess reagent (100 µL) and incubated at room temperature for 10 min. The absorbance was measured by a microplate reader (Multiskan GO, Thermo Scientific, Waltham, MA, USA) at 540 nm wavelength.

## 4. Conclusions

The chemical investigation of the ascidian-derived fungus *Diaporthe* sp. SYSU-MS4722 afforded seven new polyketides, diaporthones A−G (**1**−**7**), together with 13 known analogues, including five mono- (**8**−**14**) and six dimeric xanthones (**15**−**20**). The absolute configurations of **1**−**7** were identified by X-ray crystallographic analysis and Calculation of the ECD Spectra. Compounds **11** and **15**−**20** showed significant anti-inflammatory activity with inhibition of nitric oxide (NO) production in RAW264.7 cells activated by lipopolysaccharide with IC_50_ values between 6.3 and 8.0 μM. At the same time, dimeric xanthones (**15**−**20**) exhibited selectively growth-inhibitory effects on T98G cell lines with IC_50_ values ranging from 19.5 to 78.0 μM.

## Figures and Tables

**Table 1:** ^1^H (600 MHz) and ^13^C (150 MHz) NMR spectroscopic data of **1** in CD_3_OD.

No.	1		No.	1	
	*δ*_C_, Type	*δ*_H_, Mult (*J* in Hz)		*δ*_C_, Type	*δ*_H_, Mult (*J* in Hz)
2	83.2, C		8a	159.4, C	
3	36.7, CH_2_	2.98, br s	9	87.1, CH	4.39, d (4.2)
4	196.8, C		10	29.6, CH	2.85, m
4a	107.2,C		11	35.9, CH_2_	2.22, dd (21.6, 8.9)
5	161.4, C				2.85, m
6	106.9,CH	6.40, d (8.3)	12	177.3, C	
7	138.1, CH	7.36, t (8.3)	13	61.5, CH_2_	3.76, s
8	108.7, CH	6.43, d (8.3)	14	19.4, CH_3_	1.21, d (6.6)

**Table 2:** ^1^H (400 MHz) and ^13^C (100 MHz) NMR spectroscopic data of **2**–**4**.

No.	2 ^a^		3 ^b^		4 ^a^	
	*δ*_C_, Type	*δ*_H_, Mult (*J* in Hz)	*δ*_C_, Type	*δ*_H_, Mult (*J* in Hz)	*δ*_C_, Type	*δ*_H_, Mult (*J* in Hz)
1	163.4, C		163.1, C		162.5, C	
2	109.7, CH	6.44, dd (8.2, 0.9)	108.8, CH	6.36, d (8.7)	109.2, CH	6.41, dd (8.3, 1.0)
3	139.0, CH	7.39, t (8.3)	138.6, CH	7.36, t (8.3)	138.8, CH	7.34, t (8.3)
4	109.6, CH	6.55, d (8.2)	108.2, CH	6.39, d (8.8)	109.2, CH	6.41, dd (8.3, 1.0)
4a	159.8, C		160.9, C		161.1, C	
5	75.3, CH	4.29, m	73.9, CH	4.74, d (6.3)	71.6, CH	3.90, d (2.1)
5a	76.2, C		74.3, C		83.5, C	
6	36.2, CH	2.34, m	140.1, C		26.4, CH	2.43, m
7	29.9, CH_2_	1.57, dt (14.8, 2.8)	122.1, CH	5.60, dt (4.6, 1.8)	34.9, CH_2_	1.53, m
		2.43, m				1.84, m
8	69.6, CH	4.44, t (3.6)	68.0, CH	4.61, t (5.0)	69.4, CH	4.23, dt (4.7, 2.6)
8a	85.0, C		86.9, C		48.5, CH	2.48, d (4.5)
9	196.4, C		197.3, C		202.4, C	
9a	108.6, C		108.3, C		111.3, C	
10	60.5, CH_2_	3.83, d (13.5)	64.3, CH_2_	4.09, m	63.2, CH_2_	3.72, d (12.0)
		4.28, m				3.81, d (12.0)
11	20.7, CH_3_	1.32, d (7.8)	19.3, CH_3_	1.84, d (1.4)	18.0, CH_3_	1.06, d (7.0)
1-OH				11.26, s		
5-OH				4.84, d (6.6)		
8-OH				4.69, d (5.1)		
8a-OH				5.43, s		
10-OH				3.80, d (5.1)		

^a^ Spectra were recorded in CD_3_OD; ^b^ Spectra were recorded in acetone-*d*_6._

**Table 3:** ^1^H (400 MHz) and ^13^C (100 MHz) NMR spectroscopic data of **5**–**7**.

No.	5 ^a^		6 ^a^		7 ^b^	
	*δ*_C_, Type	*δ*_H_, Mult (*J* in Hz)	*δ*_C_, Type	*δ*_H_, Mult (*J* in Hz)	*δ*_C_, type	*δ*_H_, Mult (*J* in Hz)
1	162.0, C		164.4, C		163.4, C	
2	108.1, CH	6.42, d (8.2)	108.5, CH	6.52, d (8.2)	109.7, CH	6.48, dd, (8.3, 0.9)
3	138.2, CH	7.31, t (8.3)	139.0, CH	7.45, t (8.3)	139.0, CH	7.43, t, (8.3)
4	110.5, CH	6.52, d (8.3)	110.9, CH	6.54, d (8.2)	109.7, CH	6.59, dd, (8.3, 0.9)
4a	157.6, C		161.2, C		160.2, C	
5	70.3, CH	5.72, s	80.5, CH	4.22, s	74.5, CH	4.28, m
5a	82.4, C		78.1, C		76.3, C	
6	27.8, CH	2.39, m	82.9, C		29.2, CH	2.25, m
7	33.5, CH_2_	2.43, m	36.9, CH_2_	1.80, dt (15.2, 0.8)2.39, dd (15.2, 5.4)	31.9, CH_2_	2.25, m2.06, m
8	178.1, C		71.1, CH	4.47, dd (5.3, 0.9)	68.5, CH	4.40, t, (2.9)
8a	101.0, C		81.9, C		85.4, C	
9	187.7, C		195.6, C		196.2, C	
9a	106.7, C	3.51, d (13.2)	107.5, C		108.6, C	
10	65.6, CH_2_	4.03, d (13.2)	67.9, CH_2_	4.38, d (8.1)3.65, d (8.1)	60.4, CH_2_	4.26, d (13.4)3.78, d (13.4)
11	17.7, CH_3_	1.06, d (5.5)	23.9, CH_3_	1.33, s	18.1, CH_3_	1.12, d (6.7)
12	170.8, C					
13	21.0, CH_3_	2.09, s				
14	162.0, C					

^a^ Spectra were recorded in CDCl_3_; ^b^ Spectra were recorded in CD_3_OD.

**Table 4:** Anti-glioma activity against T98G, U87MG, and U251 human cell lines and inhibition of NO production in LPS-induced RAW264.7 cells of compounds **11** and **14**−**20**.

Compounds	T98G (IC_50_, μM)	U87MG (IC_50_, μM)	U251 (IC_50_, μM)	Inhibition of NO Production, IC_50_ (μM)
**11**	>100	>100	>100	41.4
**14**	>100	>100	>100	32.2
**15**	23.8	>100	>100	6.3
**16**	19.5	>100	>100	7.5
**17**	57.6	>100	>100	6.3
**18**	34.6	>100	>100	7.6
**19**	74.1	>100	>100	8.0
**20**	78.2	>100	>100	7.8
Temozolomide	151	203	189	-
Indomethacin	-	-	-	35.8

## Supplementary Materials

The following are available online at https://www.mdpi.com/article/10.3390/md20010051/s1, Figure S1: The HRESIMS spectrum of compound **1**, Figures S2–S7: The 1D and 2D NMR (600 MHz) spectra of compound **1** in CD_3_OD, Figure S8: The IR spectrum of compound **1**, Figure S9: The HRESIMS spectrum of compound **2**, Figures S10–S15: The 1D and 2D NMR (400 MHz) spectra of compound **2** in CD_3_OD, Figure S16: The IR spectrum of compound **2**, Figure S17: The HRESIMS spectrum of compound **3**, Figures S18–S23: The 1D and 2D NMR (400 MHz) spectra of compound **3** in acetone-*d*_6_, Figure S24: The IR spectrum of compound **3**, Figure S25: The HRESIMS spectrum of compound **4**, Figures S26–S31: The 1D and 2D NMR (400MHz) spectra of compound **4** in CD_3_OD, Figure S32: The IR spectrum of compound **4**, Figure S33: The HRESIMS spectrum of compound **5**, Figures S34–S39: The 1D and 2D NMR (400 MHz) spectra of compound **5** in CD_3_OD, Figure S40: The IR spectrum of compound **5**, Figure S41: The HRESIMS spectrum of compound **6**, Figures S42–S47: The 1D and 2D NMR (400 MHz) spectra of compound **6** in CDCl_3_, Figure S48: The IR spectrum of compound **6**, Figure S49: The HRESIMS spectrum of compound **7**, Figures S50–S55: The 1D and 2D NMR (400MHz) spectra of compound **7** in CD_3_OD, Figure S56: The IR spectrum of compound **7**.
