# Supplementary material for: Mono- and Dimeric Xanthones with Anti-Glioma and Anti-Inflammatory Activities from the Ascidian-Derived Fungus Diaporthe sp. SYSU-MS4722
Source: Mar Drugs. 2022 Jan 5;20(1):51. doi: 10.3390/md20010051 (PMC8780748; doi:10.3390/md20010051)
Supplement: Supplementary file 1 [file marinedrugs-20-00051-s001.zip › marinedrugs-1518629-supplementary.pdf]

Supporting Information

Mono- and dimeric xanthones with anti-glioma and anti-inflammatory from the ascidian-derived fungus *Diaporthe* sp. SYSU-MS4722

Senhua Chen [1,2], Heng Guo [1,2], Minghua Jiang [1,2], Qilin Wu [1,2], Jing Li [1,2], Hongjie Shen [1,2,*] and Lan Liu [1,2,3,4,*]

[1] School of Marine Sciences, Sun Yat-sen University, Zhuhai 519082, China; chensenh@mail.sysu.edu.cn.； hengeguo163@163.com； jiangmh23@mail2.sysu.edu.cn； wuqlin3@mail2.sysu.edu.cn; lijing356@mail.sysu.edu.cn

[2] Southern Laboratory of Ocean Science and Engineering, Zhuhai 519082, China

[3] Guangdong Provincial Key Laboratory of Marine Resources and Coastal Engineering, Zhuhai 519082, China.

[4] Pearl River Estuary Marine Ecosystem Research Station, Ministry of Education, Zhuhai 519082, China.

* Correspondence: shenhj5@mail2.sysu.edu.cn; cesllan@mail.sysu.edu.cn; Tel.: (optional; include country code; if there are multiple corresponding authors, add author initials)

Figure S1. The HRESIMS spectrum of compound **1**. 4

Figure S2. The ^{1}H NMR (600MHz) spectrum of compound **1** in CD_3OD. 4

Figure S3. The ^{13}C NMR (150MHz) spectrum of compound **1** in CD_3OD. 5

Figure S4. The HSQC spectrum of compound **1** in CD_3OD. 5

Figure S5. The ^{1}H-^{1}H COSY spectrum of compound **1** in CD_3OD. 6

Figure S6. The HMBC spectrum of compound **1** in CD_3OD. 6

Figure S7. The NOESY spectrum of compound **1** in CD_3OD. 7

Figure S8. The IR spectrum of compound **1** 7

Figure S9. The HRESIMS spectrum of compound **2**. 8

Figure S10. The ^{1}H NMR (400MHz) spectrum of compound **2** in CD_3OD. 8

Figure S11. The ^{13}C NMR (100MHz) spectrum of compound **2** in CD_3OD. 9

Figure S12. The HSQC spectrum of compound **2** in CD_3OD. 9

Figure S13. The ^{1}H-^{1}H COSY spectrum of compound **2** in CD_3OD. 10

Figure S14. The HMBC spectrum of compound **2** in CD_3OD. 10

Figure S15. The NOESY spectrum of compound **2** in CD_3OD. 11

Figure S16. The IR spectrum of compound **2** 11
Figure S17. The HRESIMS spectrum of compound **3**........ 12
Figure S18. The ^{1}H NMR (400MHz) spectrum of compound **3** in acetone-d_6. 12
Figure S19. The ^{13}C NMR (100MHz) spectrum of compound **3** in acetone-d_6. 13
Figure S20. The HSQC spectrum of compound **3** in acetone-d_6........ 13
Figure S21. The ^{1}H-^{1}H COSY spectrum of compound **3** in acetone-d_6........ 14
Figure S22. The HMBC spectrum of compound **3** in acetone-d_6........ 14
Figure S23. The NOESY spectrum of compound **3** in acetone-d_6. 15
Figure S24. IR spectrum of compound **3**. 15
Figure S25. The HRESIMS spectrum of compound **4**........ 16
Figure S26. The ^{1}H NMR (400MHz) spectrum of compound **4** in CD_3OD. 16
Figure S27. The ^{13}C NMR (100MHz) spectrum of compound **4** in CD_3OD. 17
Figure S28. The HSQC spectrum of compound **4** in CD_3OD. 17
Figure S29. The ^{1}H-^{1}H COSY spectrum of compound **4** in CD_3OD. 18
Figure S30. The HMBC spectrum of compound **4** in CD_3OD. 18
Figure S31. The NOESY spectrum of compound **4** in CD_3OD........ 19
Figure S32. IR spectrum of compound **4**. 19
Figure S33. The HRESIMS spectrum of compound **5**........ 20
Figure S34. The ^{1}H NMR (400MHz) spectrum of compound **5** in $CDCl_3$. 20
Figure S35. The ^{13}C NMR (100MHz) spectrum of compound **5** in $CDCl_3$. 21
Figure S36. The HSQC spectrum of compound **5** in $CDCl_3$. 21
Figure S37. The ^{1}H-^{1}H COSY spectrum of compound **5** in $CDCl_3$. 22
Figure S38. The HMBC spectrum of compound **5** in $CDCl_3$. 22
Figure S39. The NOESY spectrum of compound **5** in $CDCl_3$........ 23
Figure S40. IR spectrum of compound **5**. 23
Figure S41. The HRESIMS spectrum of compound **6**........ 24
Figure S44. The HSQC spectrum of compound **6** in $CDCl_3$. 25
Figure S45. The ^{1}H-^{1}H COSY spectrum of compound **6** in $CDCl_3$. 26
Figure S46. The HMBC spectrum of compound **6** in $CDCl_3$. 26
Figure S47. The NOESY spectrum of compound **6** in $CDCl_3$........ 27
Figure S48. IR spectrum of compound **6**. 27
Figure S49. The HRESIMS spectrum of compound **7** CD_3OD........ 28

Figure S50. The ^{1}H NMR (400MHz) spectrum of compound **7** in CD_3OD. 28

Figure S51. The ^{13}C NMR (100MHz) spectrum of compound **7** in CD_3OD. 29

Figure S52. The HSQC spectrum of compound **7** in CD_3OD. 29

Figure S53. The ^{1}H-^{1}H COSY spectrum of compound **7** in CD_3OD. 30

Figure S54. The HMBC spectrum of compound **7** in CD_3OD. 30

Figure S55. The NOESY spectrum of compound **7** in CD_3OD. 31

Figure S56. IR spectrum of compound **7**. 31

Experimental section: 32

Table S1. Energy of all conformers of diaporthone A (**1**). 32

Figure S57. B3LYP/6-311g** optimized low-energy conformers of diaporthone A (**1**). 32

Table S2. Cartesian coordinates of the low-energy reoptimized conformers of **1** calculated at b3lyp/6-311g** level of theory in PCM for methanol. 32

Table S3. Energy of all conformers of diaporthone C (**3**). 36

Figure S58. B3LYP/6-311g** optimized low-energy conformers of diaporthone C (**3**). 36

Table S4. Cartesian coordinates of the low-energy reoptimized conformers of **3** calculated at b3lyp/6-311g** level of theory in PCM for methanol. 36

Table S5. Energy of all conformers of diaporthone D (**4**). 38

Figure S59. B3LYP/6-311g** optimized low-energy conformers of diaporthone D (**4**). 39

Table S6. Cartesian coordinates of the low-energy reoptimized conformers of **4** calculated at b3lyp/6-311g** level of theory in PCM for methanol. 39

Table S7. Energy of all conformers of diaporthone E (**5**). 51

Figure S60. B3LYP/6-311g** optimized low-energy conformers of diaporthone E (**5**). 51

Table S8. Cartesian coordinates of the low-energy reoptimized conformers of **5** calculated at b3lyp/6-311g** level of theory in PCM for methanol. 51

Table S9. Energy of all conformers of compound **7.** 54

Figure S61. B3LYP/6-311g** optimized low-energy conformers of compound **7.** 55

Table S10. Cartesian coordinates of the low-energy reoptimized conformers of **7** calculated at b3lyp/6-311g** level of theory in PCM for methanol. 55

Figure S62. Plausible biogenetic pathway leading to a formation of 2 and (±)-**7**. 70

Figure S63. Experimental ECD spectra of **2**, **3**, (+) and (-)-**7** (in MeOH). 70

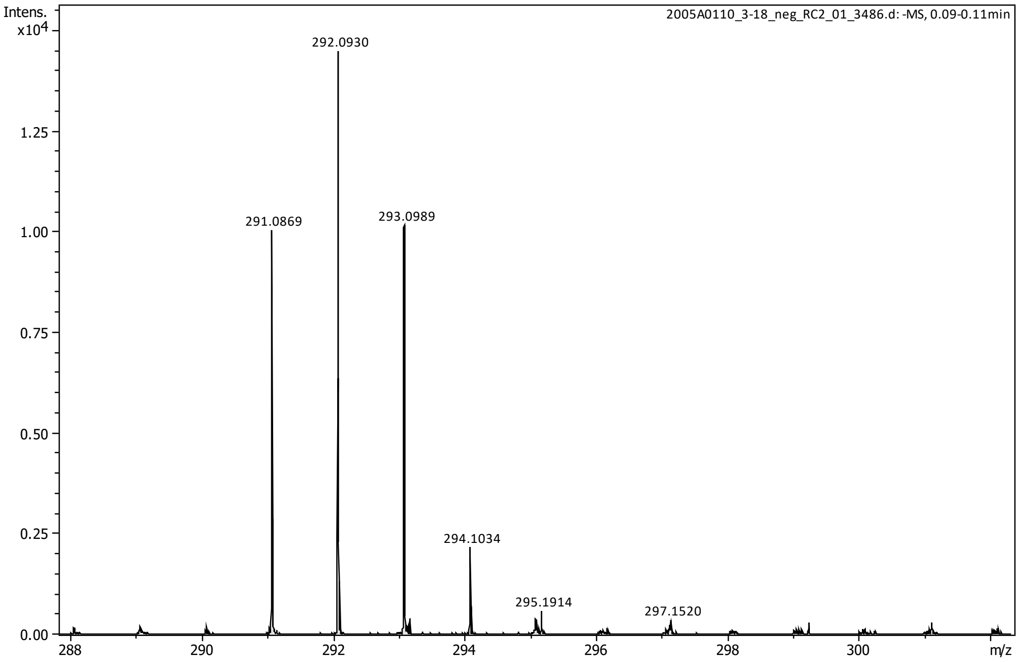

Meas. m/z	Ion Formula	m/z	z	err [ppm]	err [mDa]	mSigma	rdb	e⁻ Conf	N-Rule
291.0869	C15H15O6	291.0874	1-	1.8	0.5	644.6	8	even	ok

Figure S1. The HRESIMS spectrum of compound **1**.

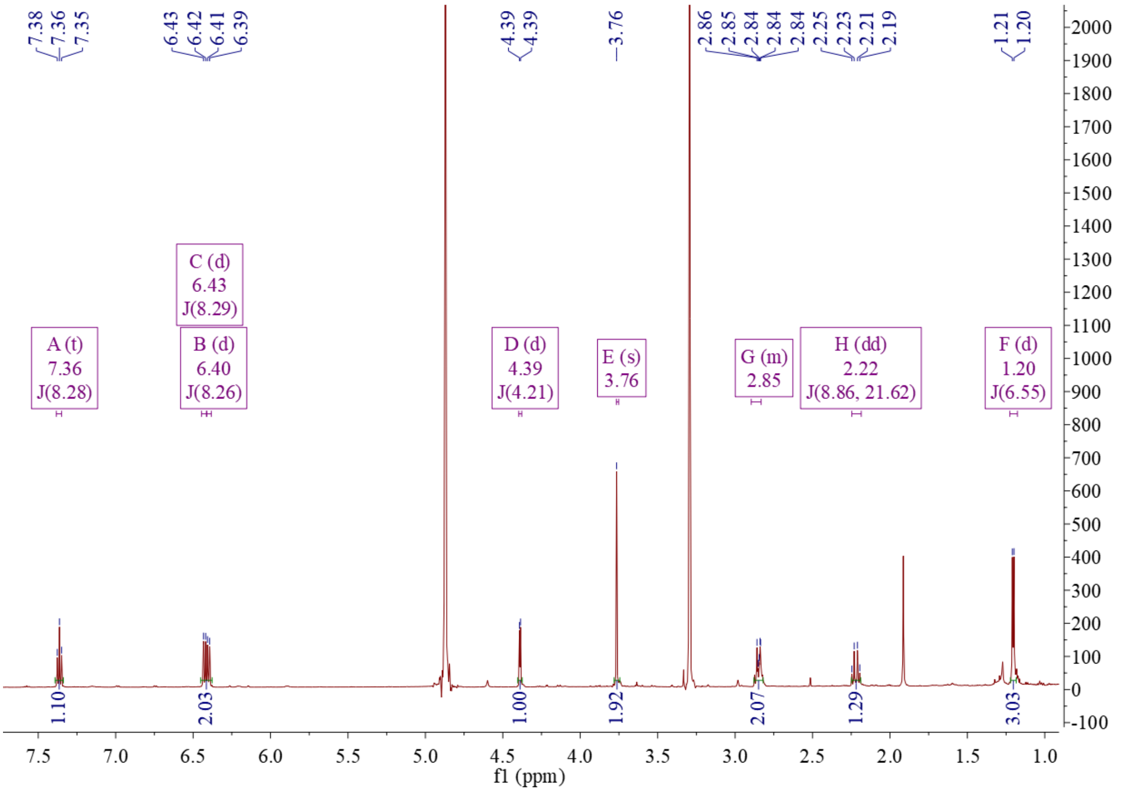

Figure S2. The ^{1}H NMR (600MHz) spectrum of compound **1** in CD_3OD.

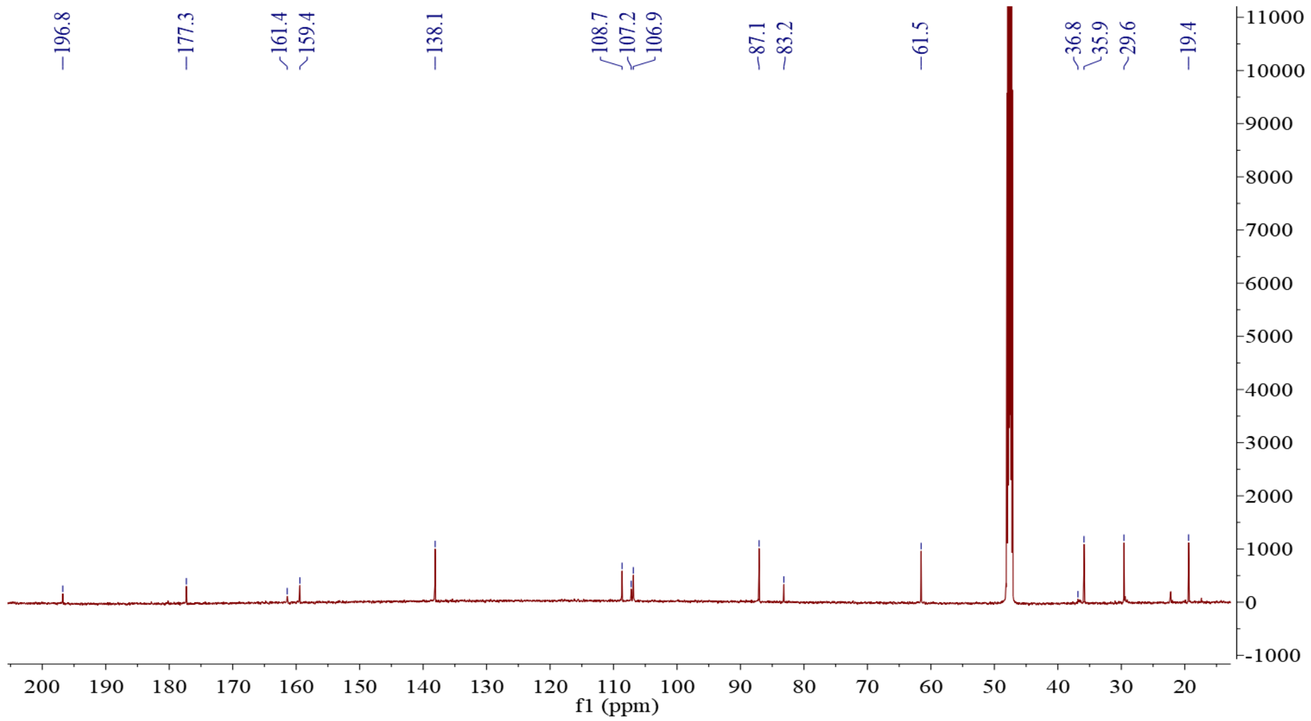

Figure S3. The ^{13}C NMR (150MHz) spectrum of compound **1** in CD_3OD.

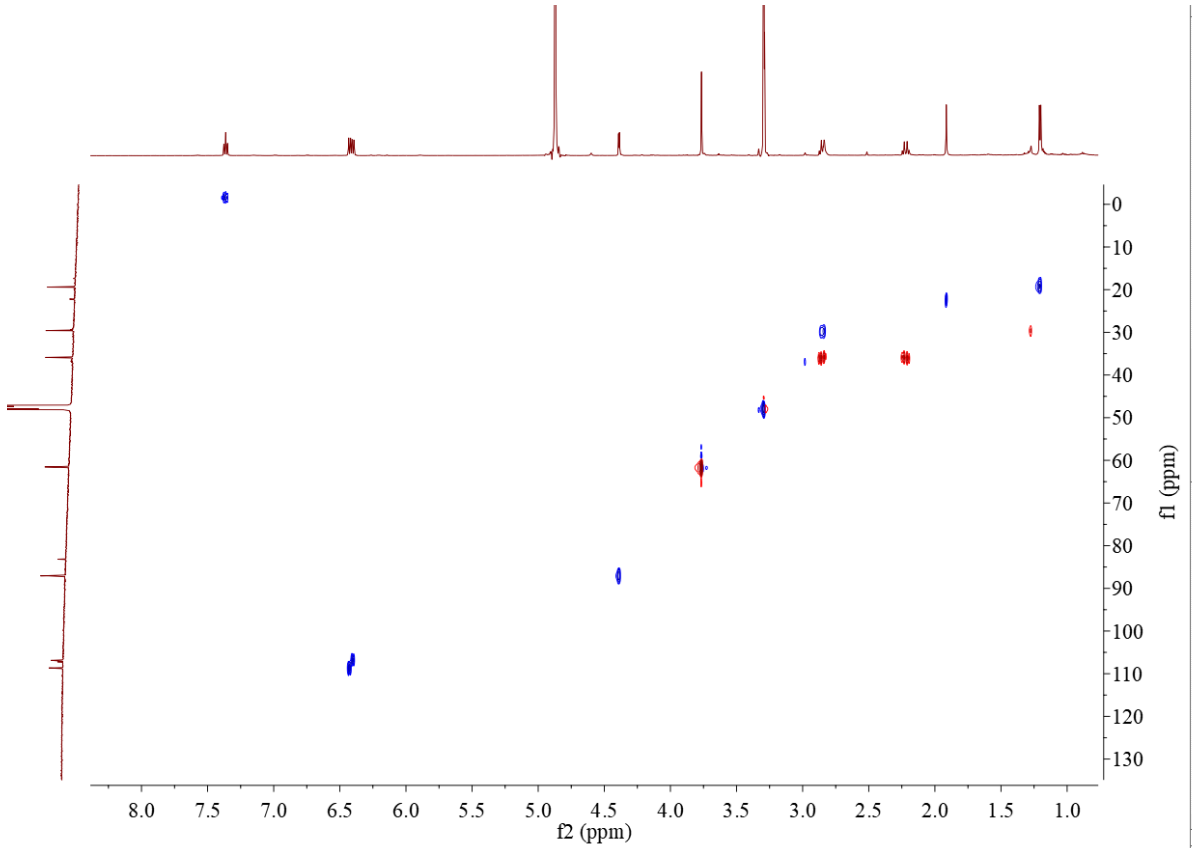

Figure S4. The HSQC spectrum of compound **1** in CD_3OD.

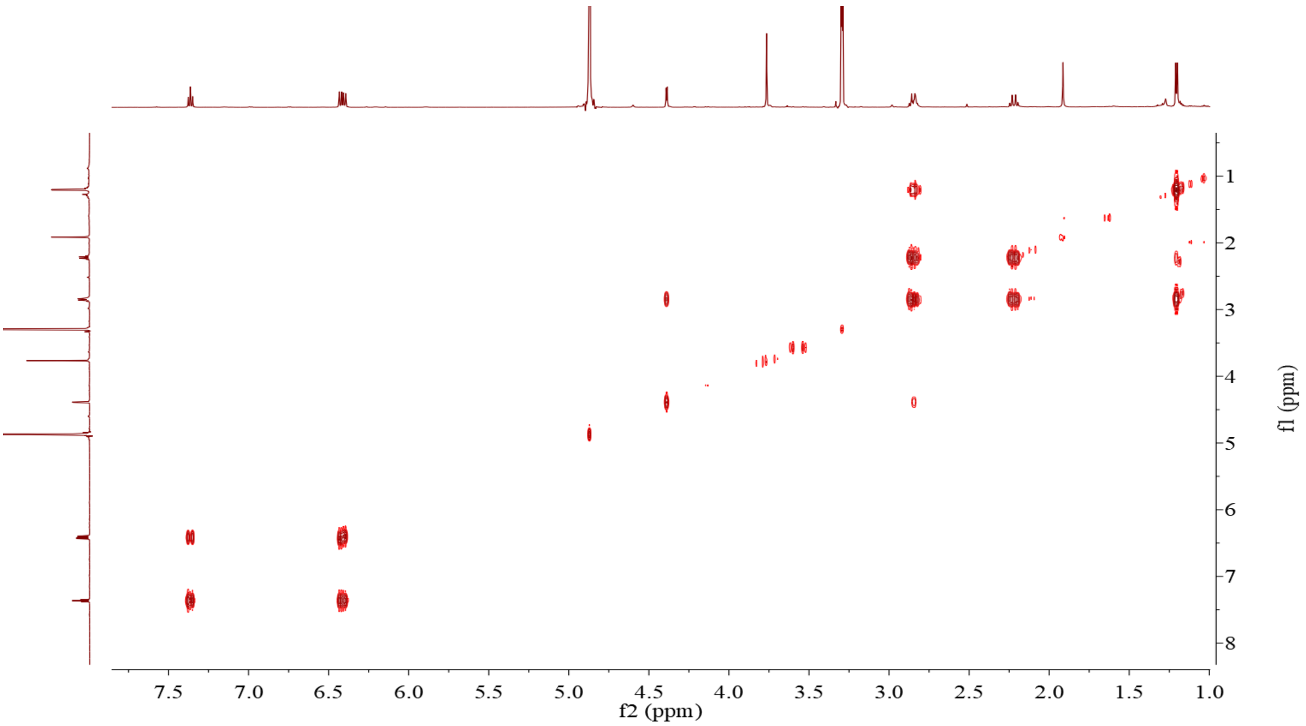

Figure S5. The ^{1}H-^{1}H COSY spectrum of compound **1** in CD_3OD.

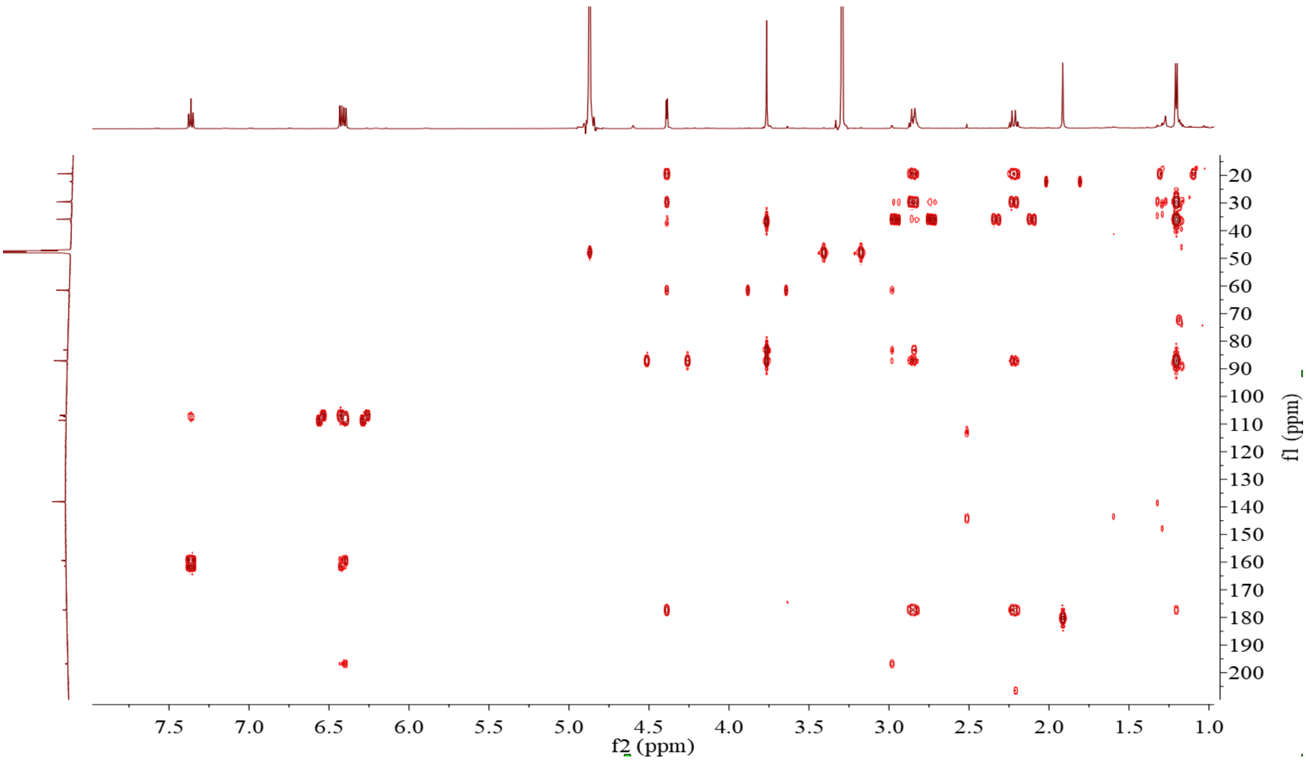

Figure S6. The HMBC spectrum of compound **1** in CD_3OD.

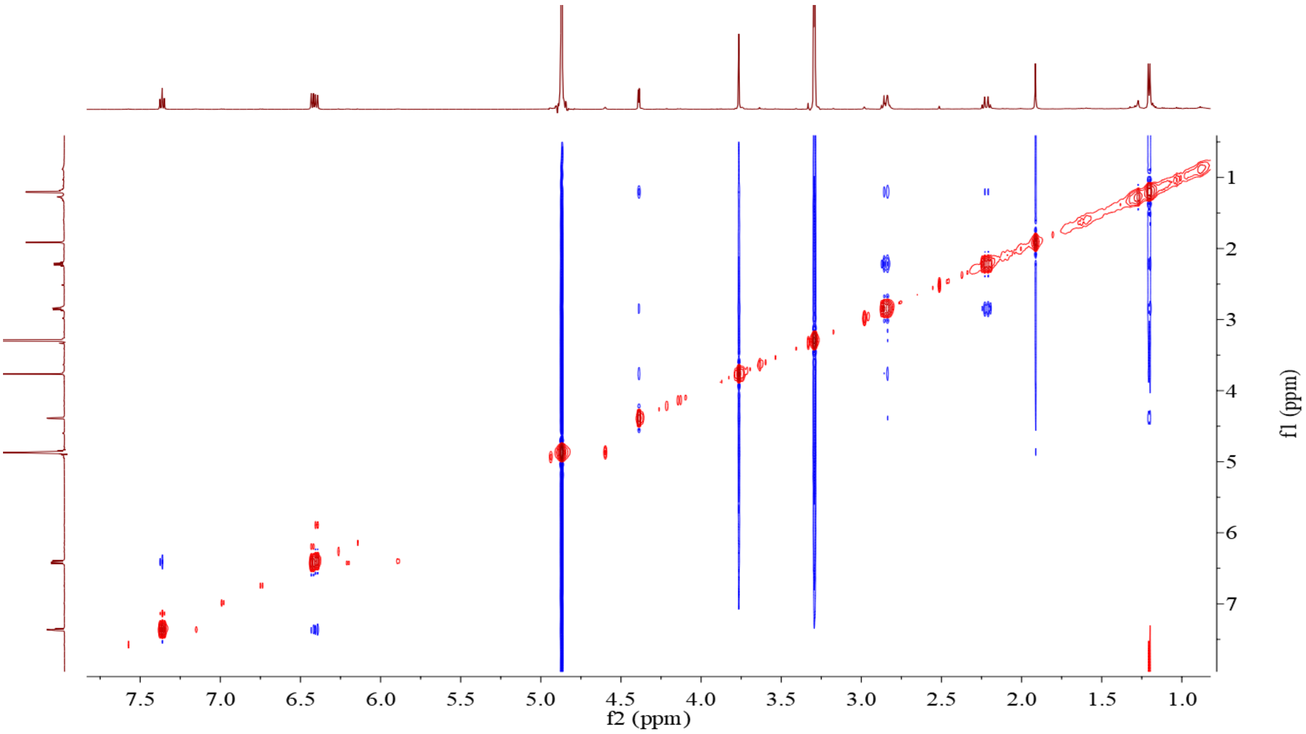

Figure S7. The NOESY spectrum of compound **1** in CD_3OD

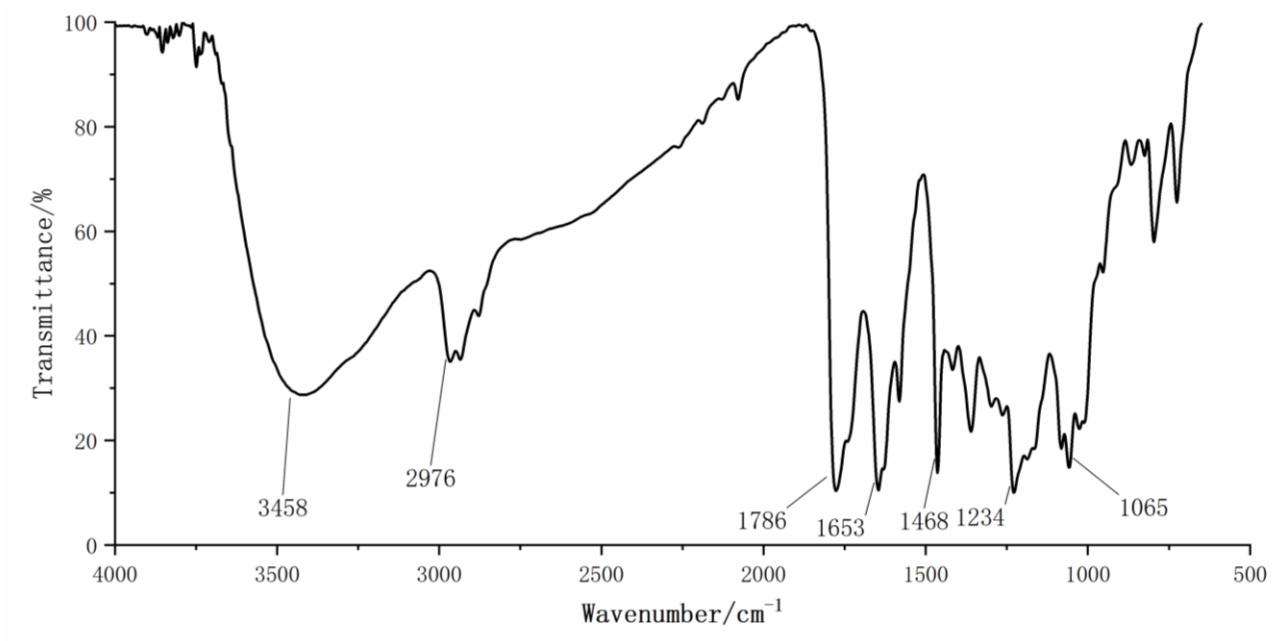

Figure S8. The IR spectrum of compound **1**

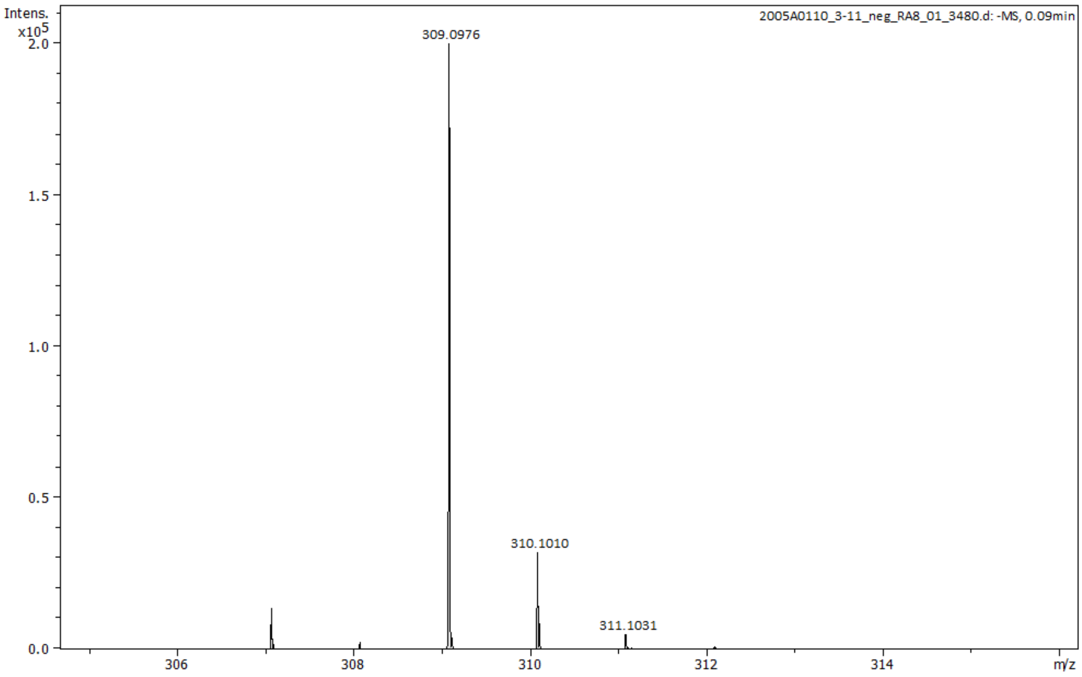

Meas. m/z	Ion Formula	m/z	z	err [ppm]	err [mDa]	mSigma	rdb	e⁻ Conf	N-Rule
309.0976	C15H17O7	309.0980	1-	1.1	0.3	4.4	7	even	ok

Figure S9. The HRESIMS spectrum of compound **2**.

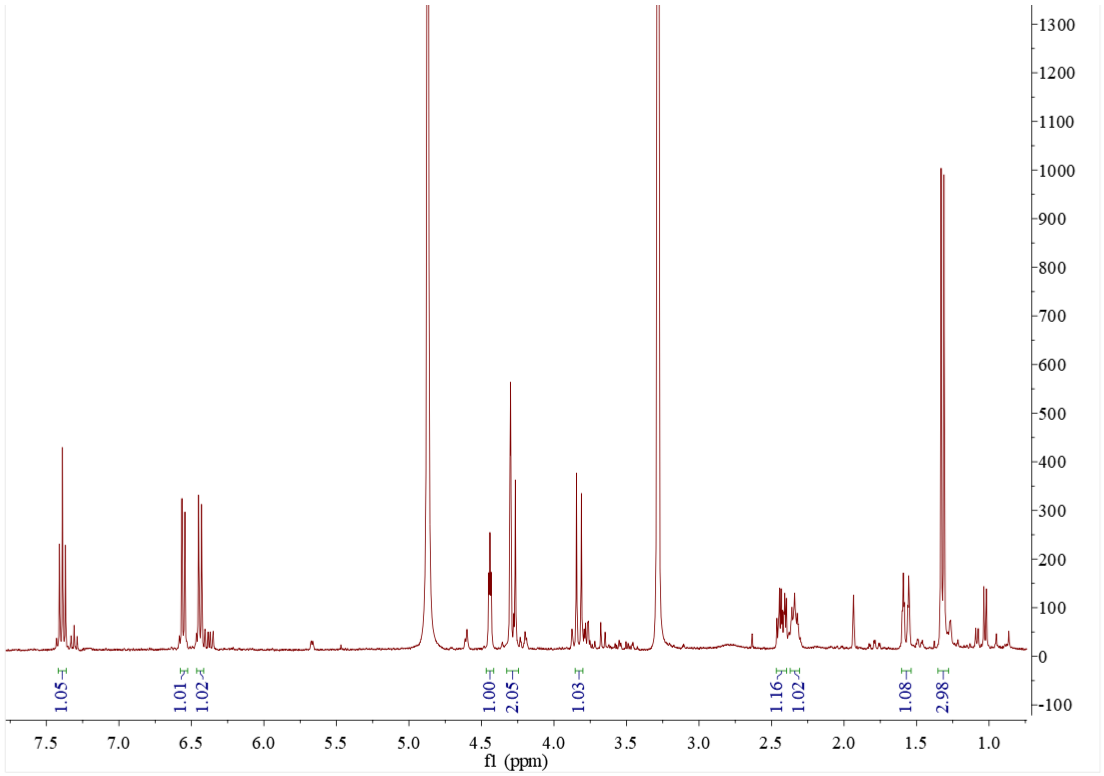

Figure S10. The ^{1}H NMR (400MHz) spectrum of compound **2** in CD_3OD.

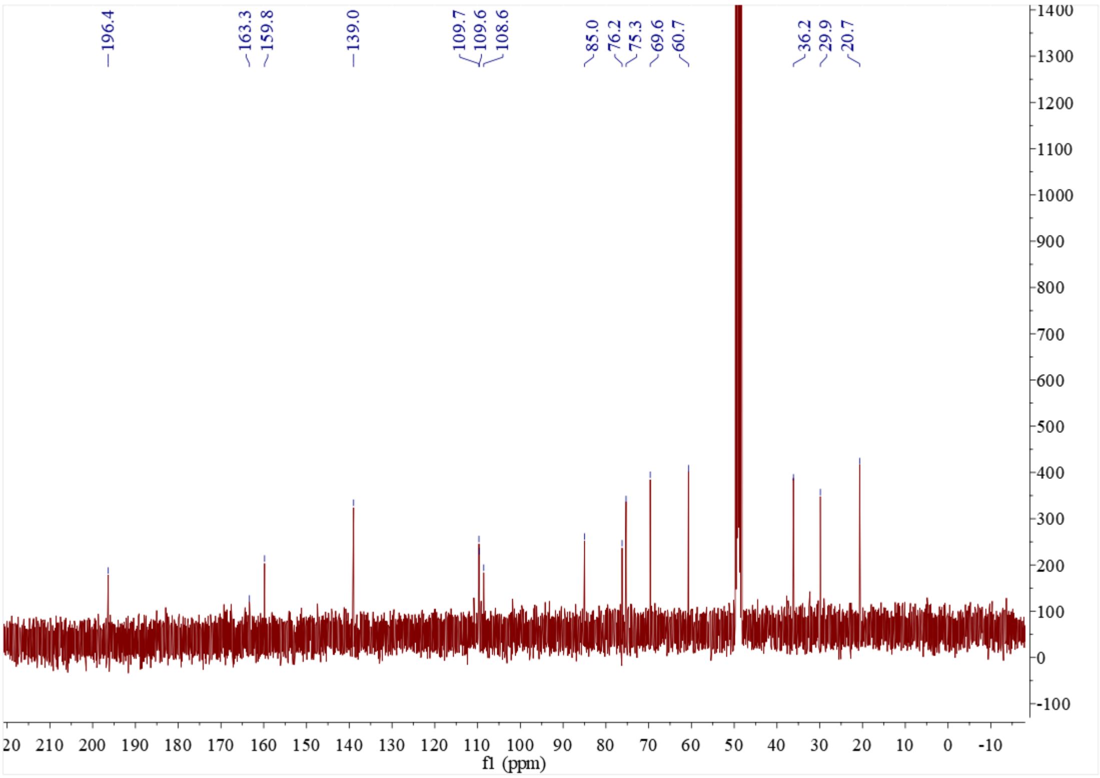

Figure S11. The ^{13}C NMR (100MHz) spectrum of compound **2** in CD_3OD.

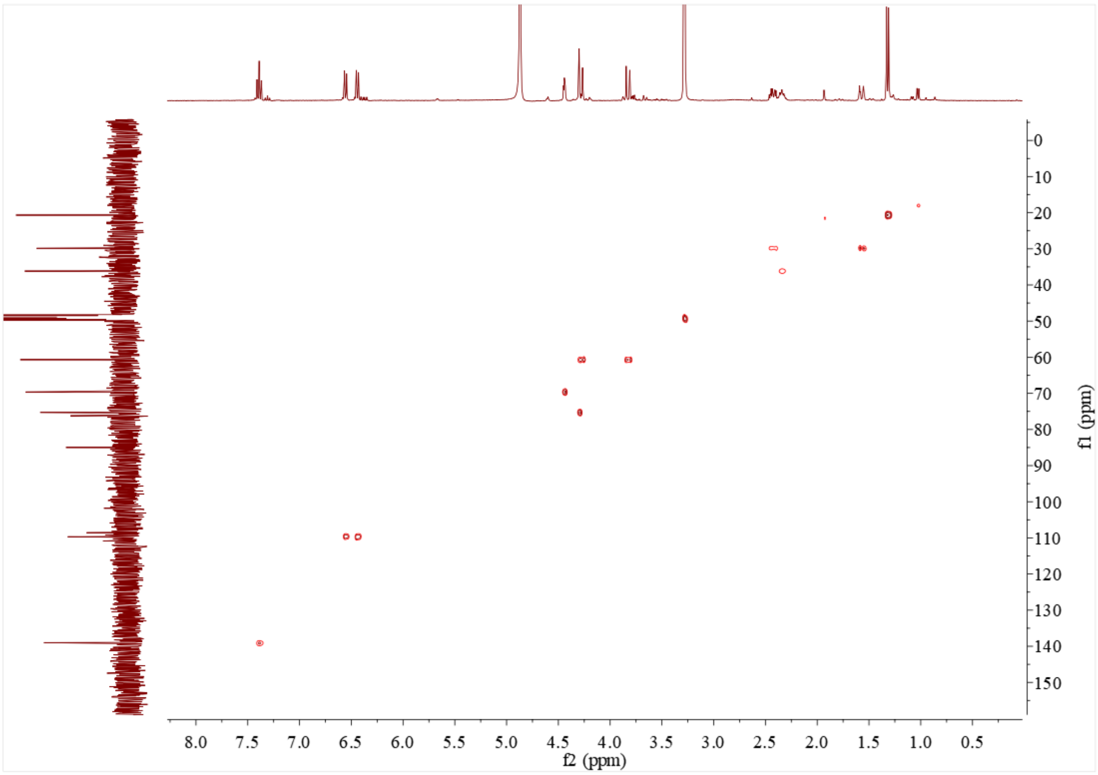

Figure S12. The HSQC spectrum of compound **2** in CD_3OD.

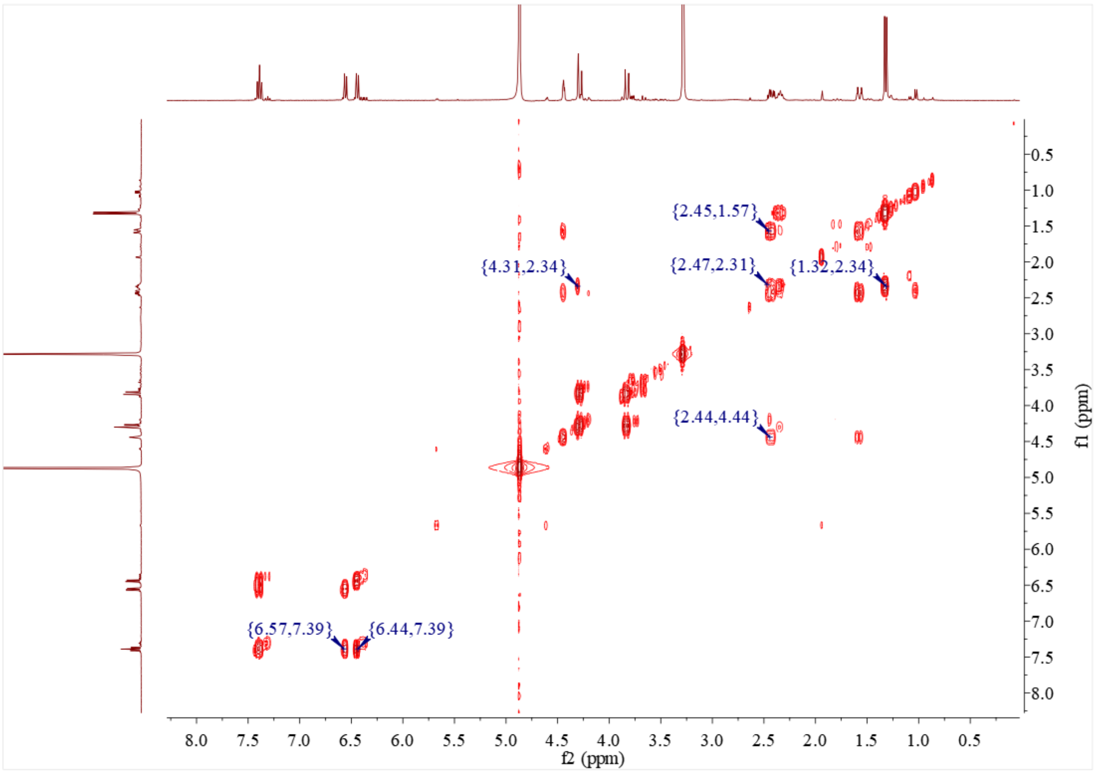

Figure S13. The ^{1}H-^{1}H COSY spectrum of compound **2** in CD_3OD.

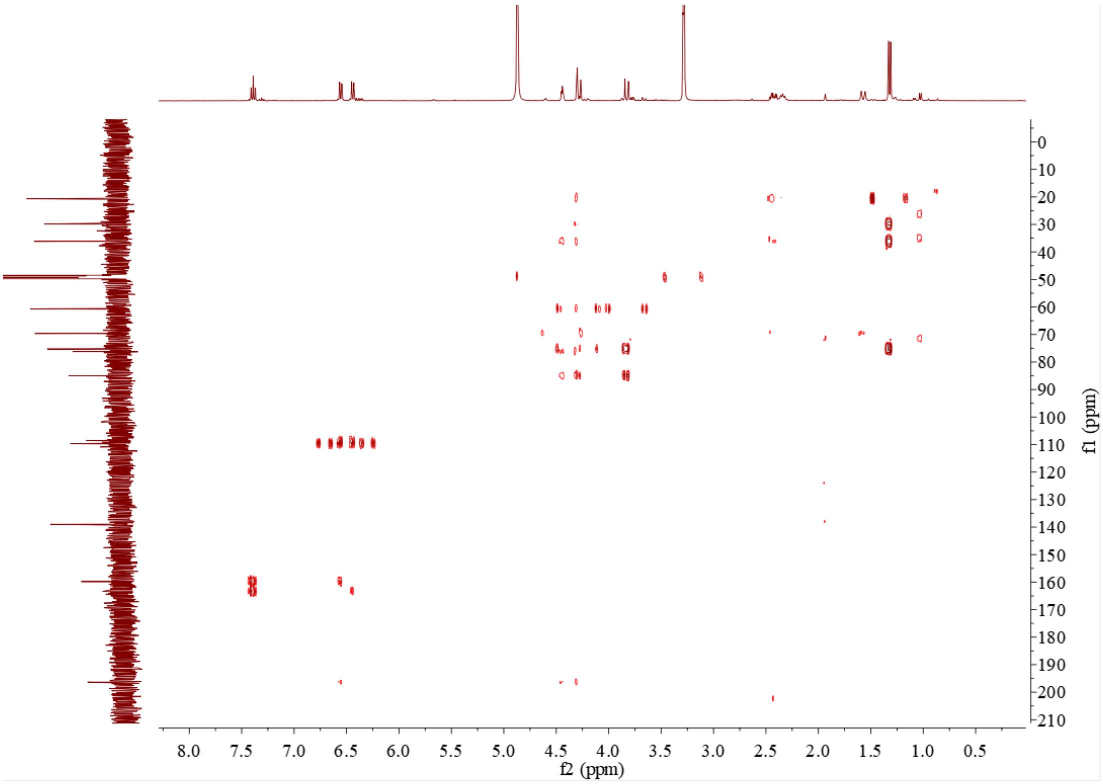

Figure S14. The HMBC spectrum of compound **2** in CD_3OD.

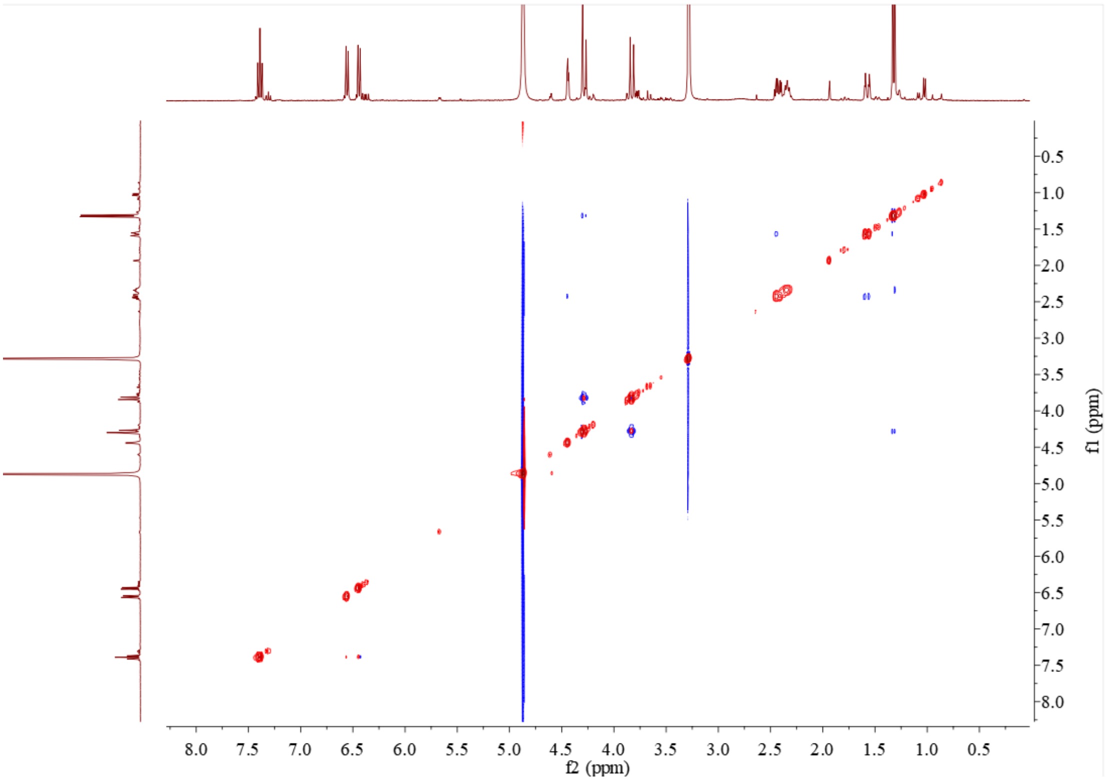

Figure S15. The NOESY spectrum of compound **2** in CD_3OD.

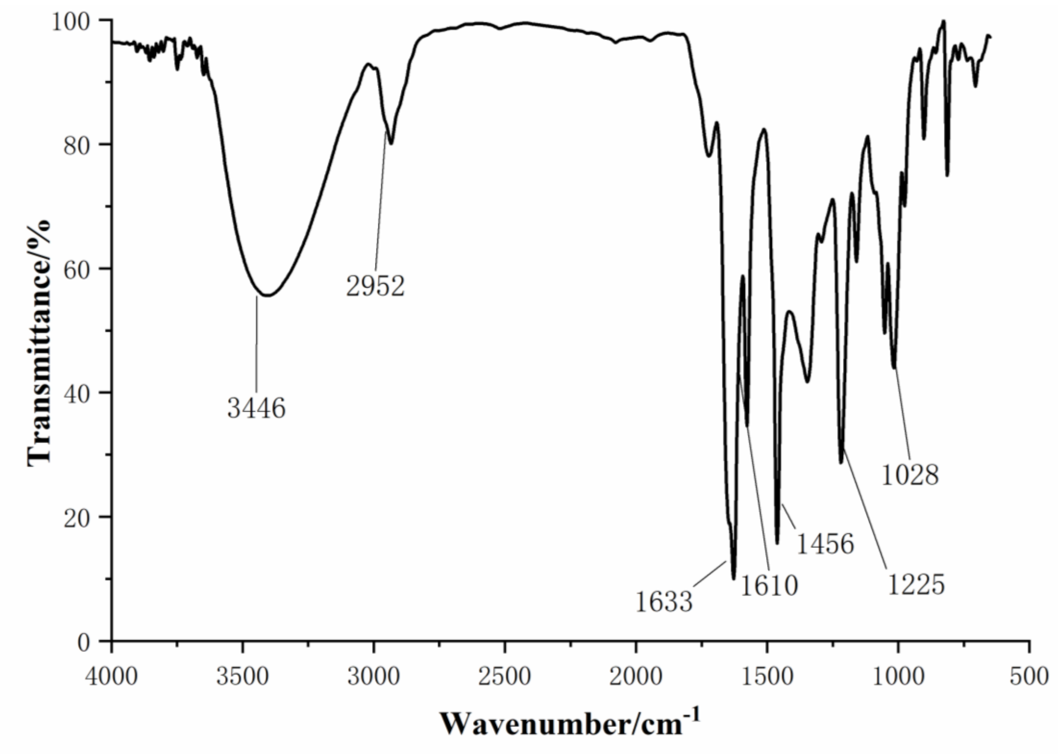

Figure S16. The IR spectrum of compound **2**

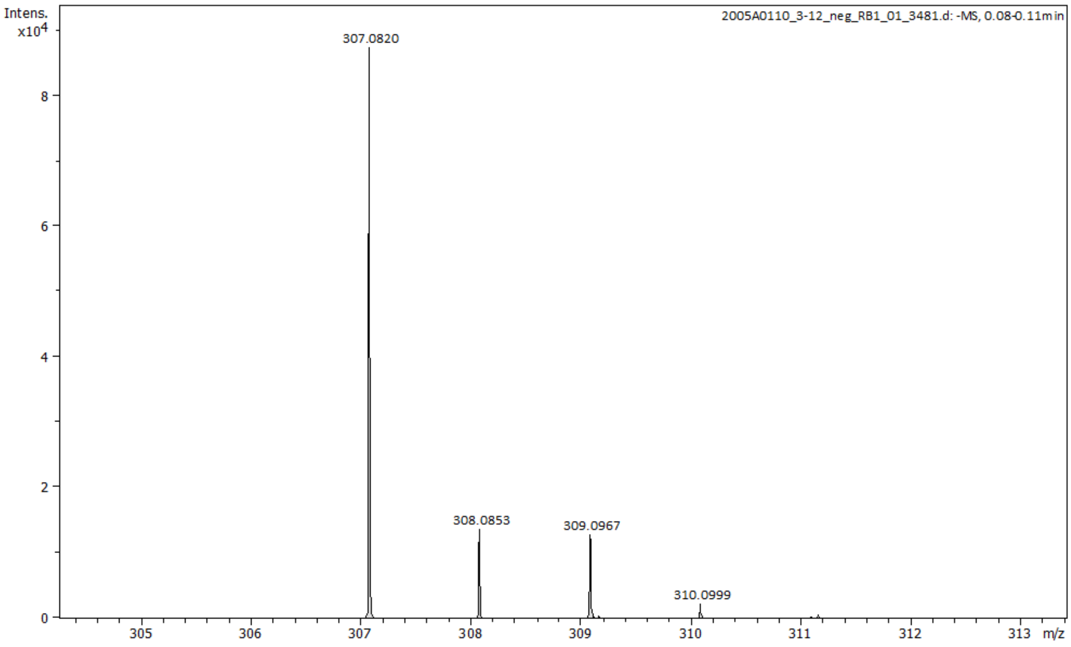

Meas. m/z	Ion Formula	m/z	z	err [ppm]	err [mDa]	mSigma	rdb	e⁻ Conf	N-Rule
307.082	C15H15O7	307.0823	1-	1.2	0.4	70	8	even	ok

Figure S17. The HRESIMS spectrum of compound **3**.

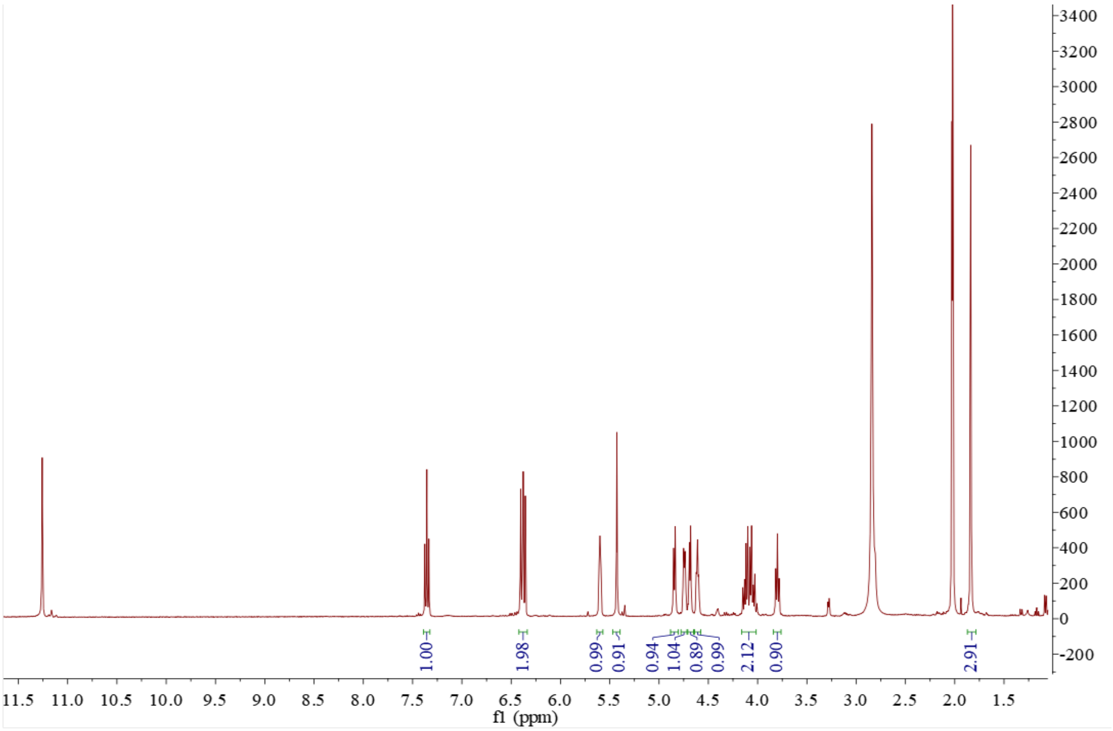

Figure S18. The ^{1}H NMR (400MHz) spectrum of compound **3** in acetone-d_6.

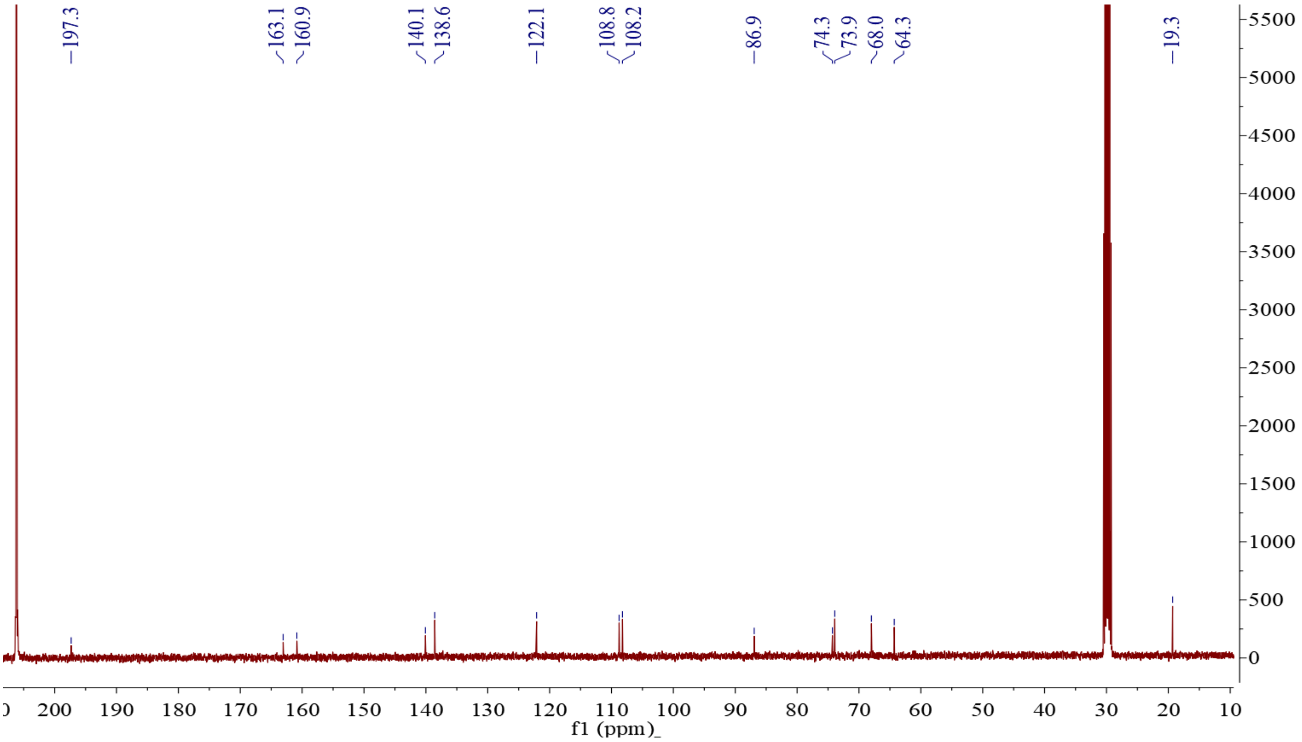

Figure S19. The ^{13}C NMR (100MHz) spectrum of compound **3** in acetone-d_6.

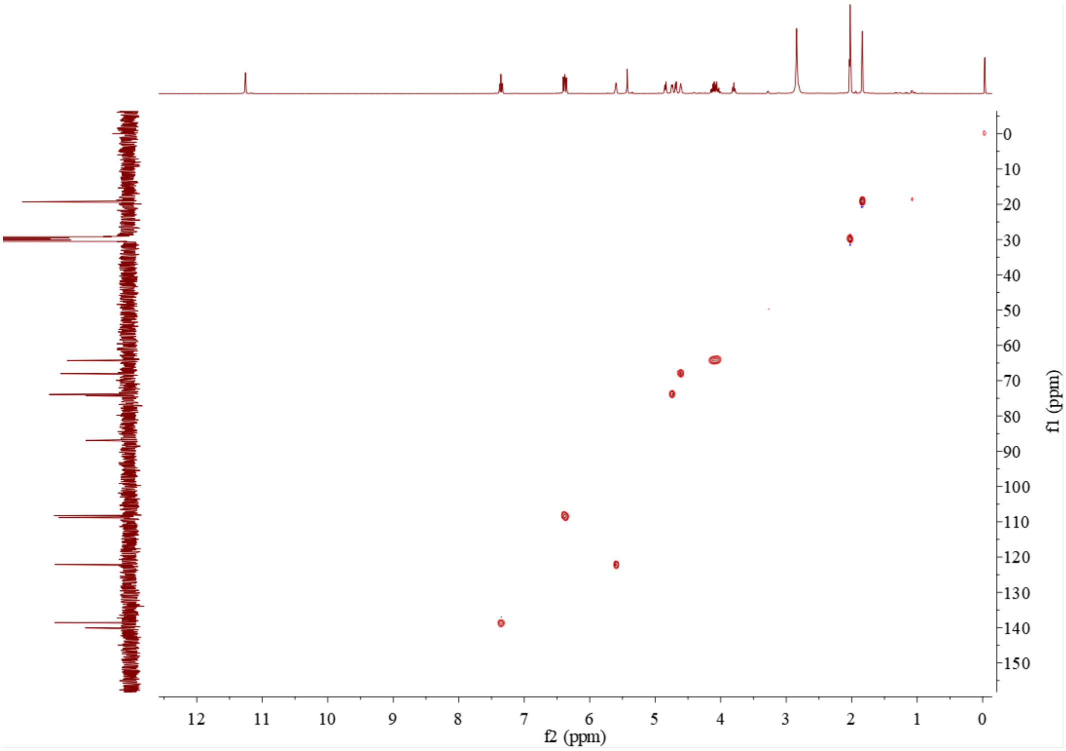

Figure S20. The HSQC spectrum of compound **3** in acetone-d_6.

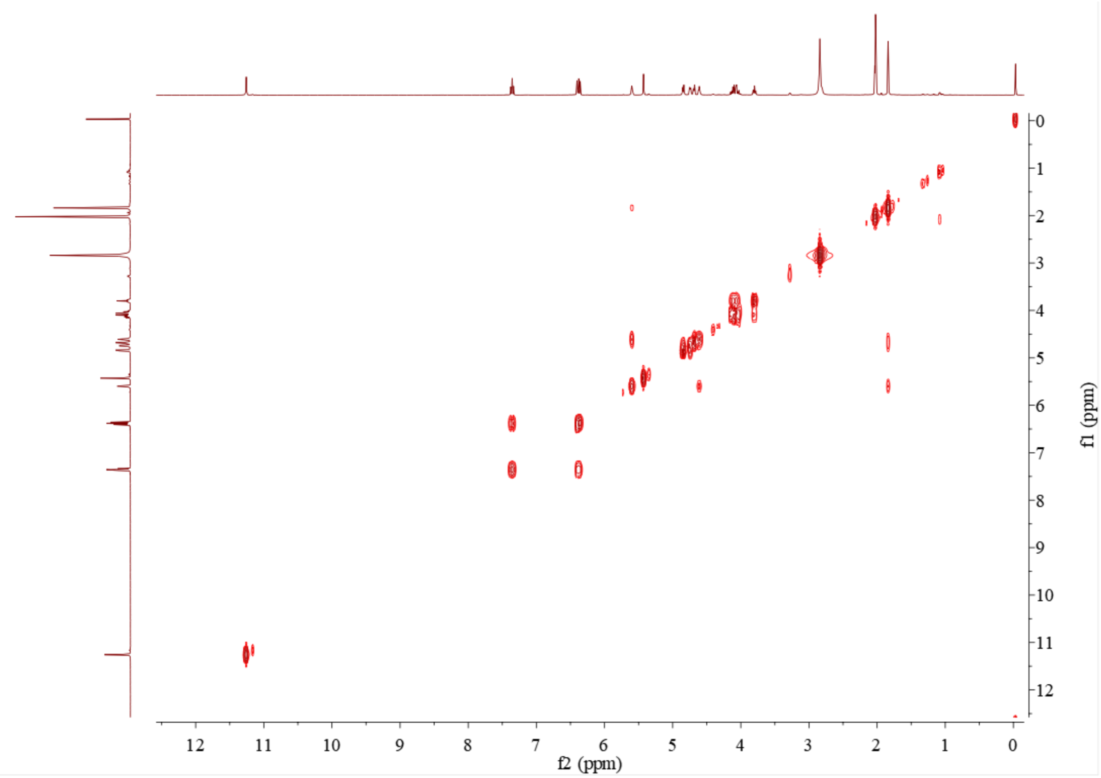

Figure S21. The ^{1}H-^{1}H COSY spectrum of compound **3** in acetone-d_6.

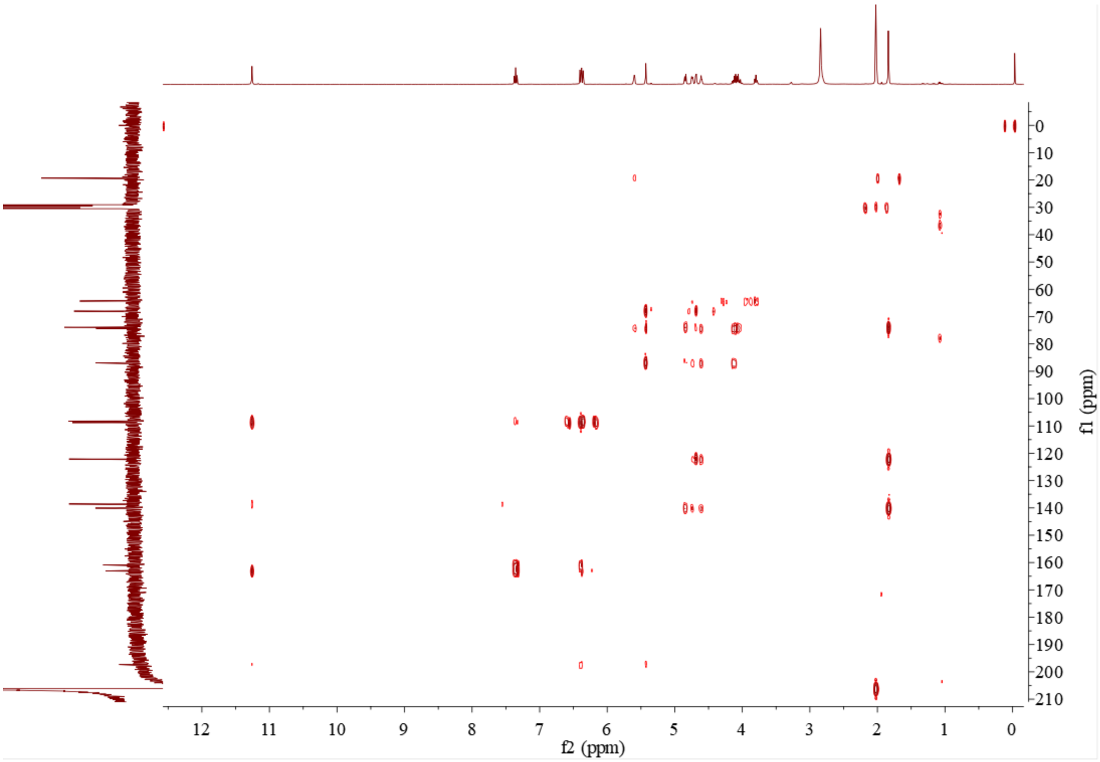

Figure S22. The HMBC spectrum of compound **3** in acetone-d_6.

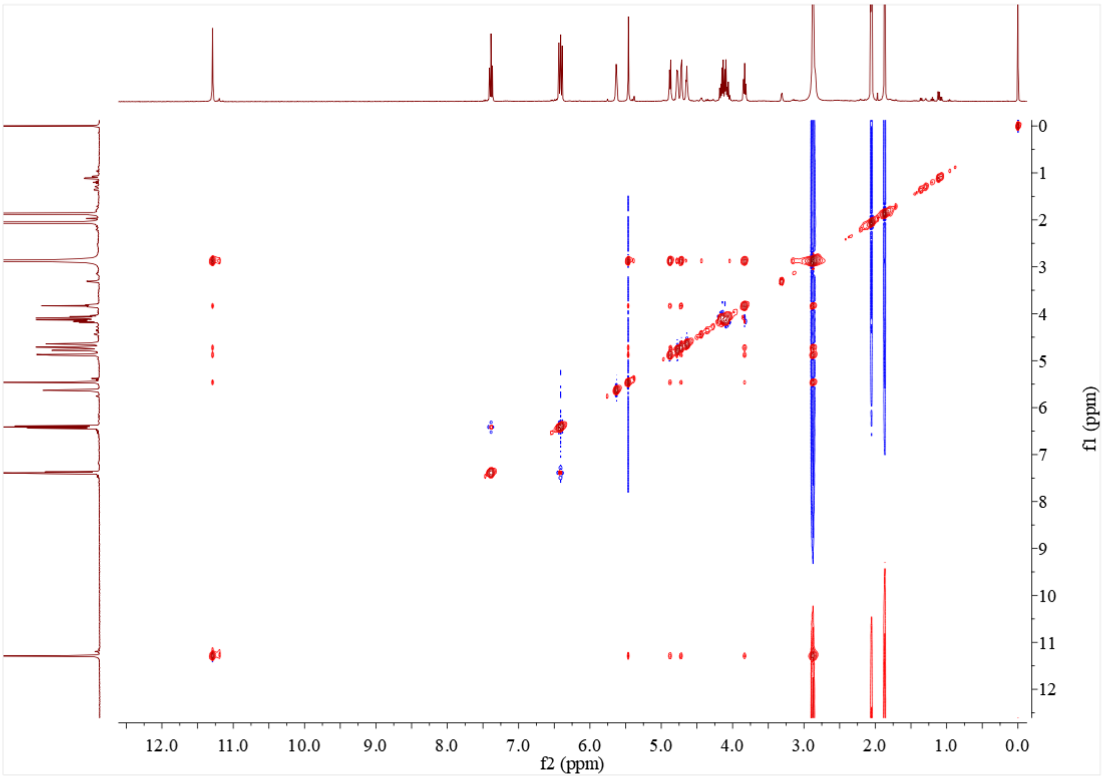

Figure S23. The NOESY spectrum of compound **3** in acetone-d_6.

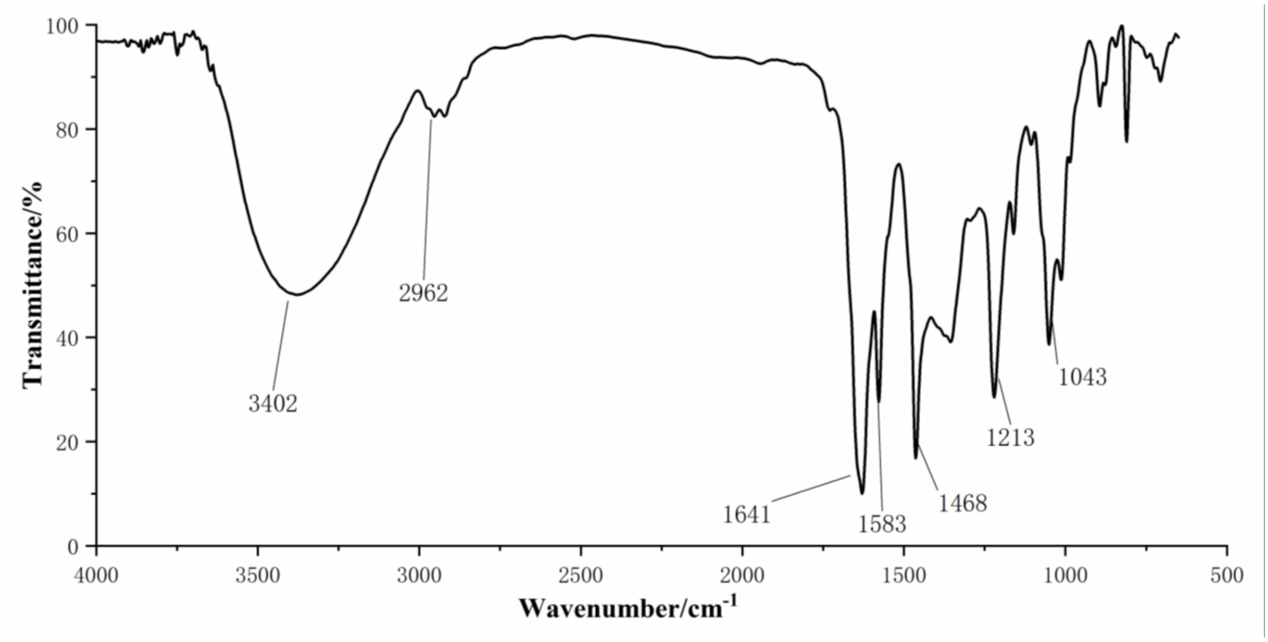

Figure S24. IR spectrum of compound **3**.

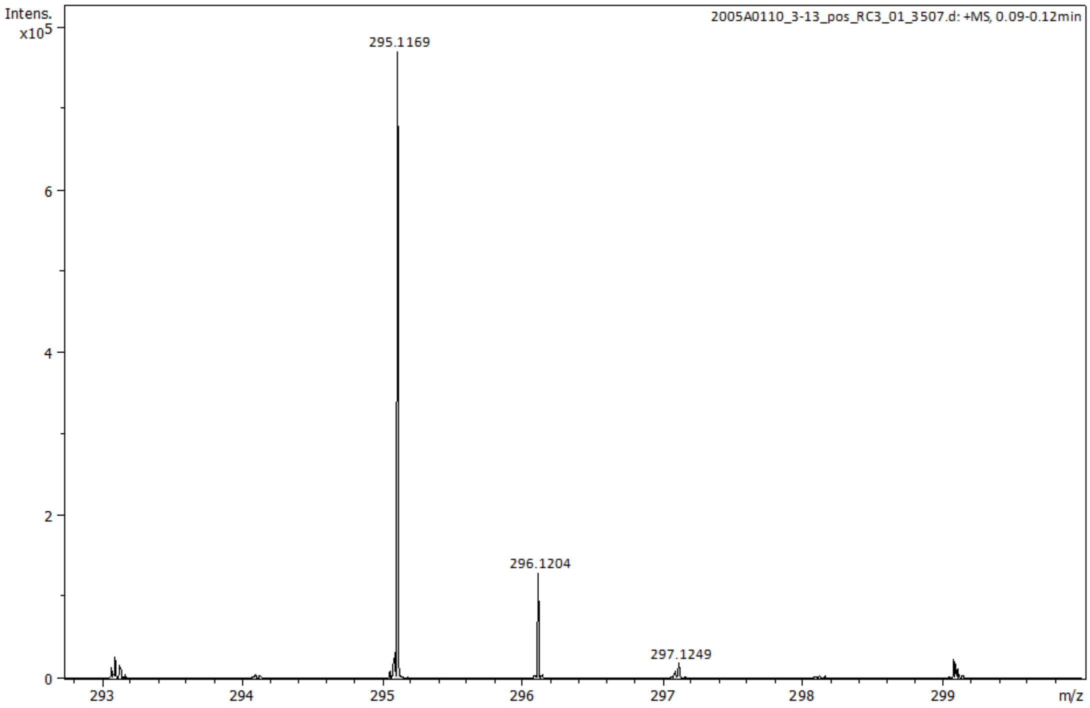

Meas. m/z	Ion Formula	m/z	z	err [ppm]	err [mDa]	mSigma	rdb	e⁻ Conf	N-Rule
295.1169	C15H19O6	295.1176	1+	2.3	0.7	1	7	even	ok

Figure S25. The HRESIMS spectrum of compound **4**.

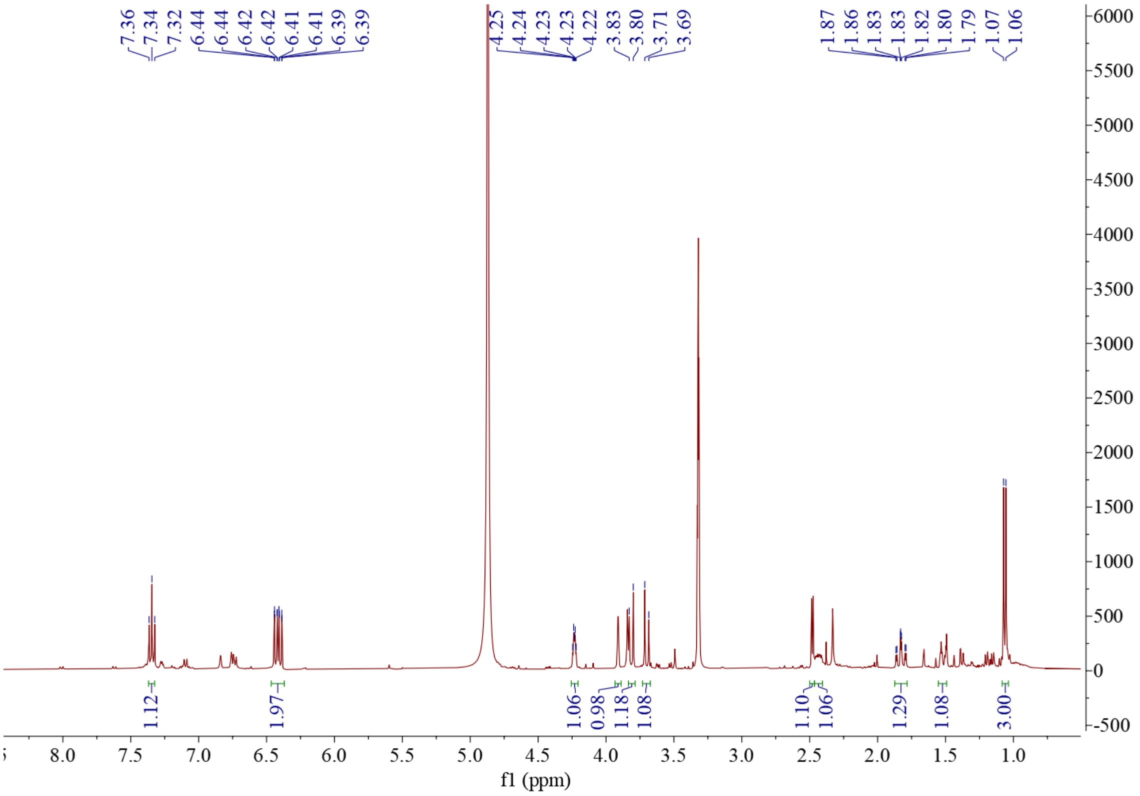

Figure S26. The ^{1}H NMR (400MHz) spectrum of compound **4** in CD_3OD.

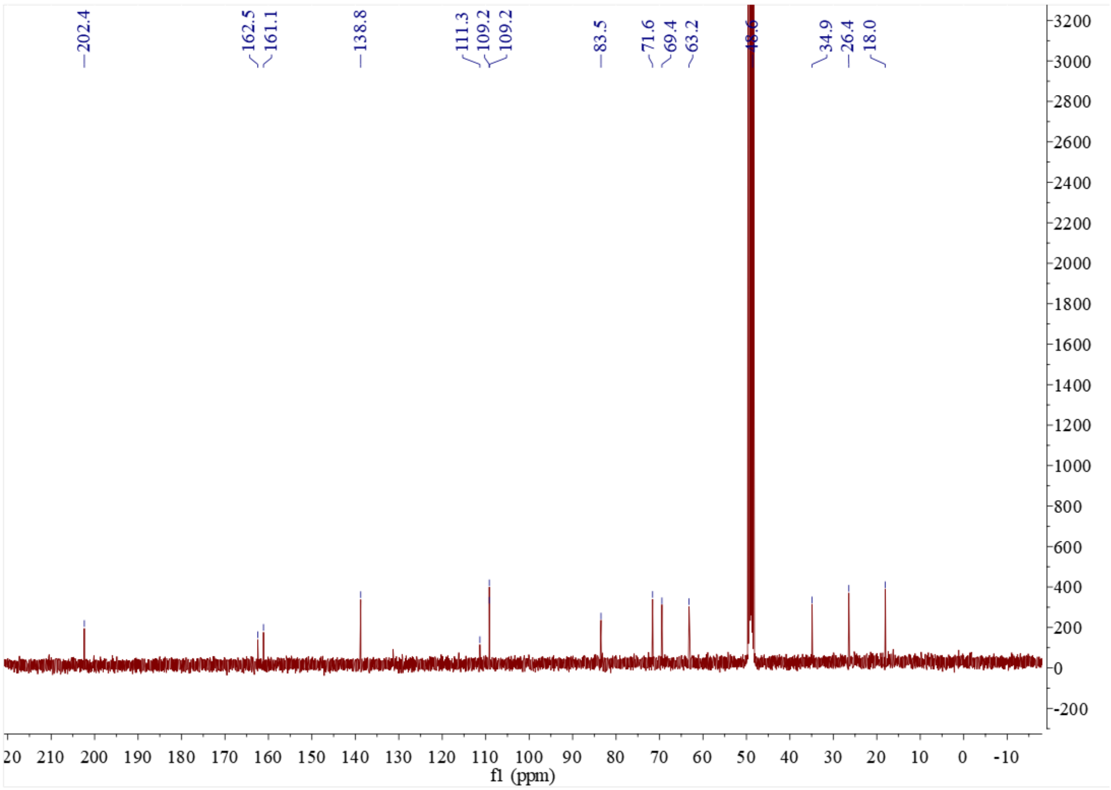

Figure S27. The ^{13}C NMR (100MHz) spectrum of compound **4** in CD_3OD.

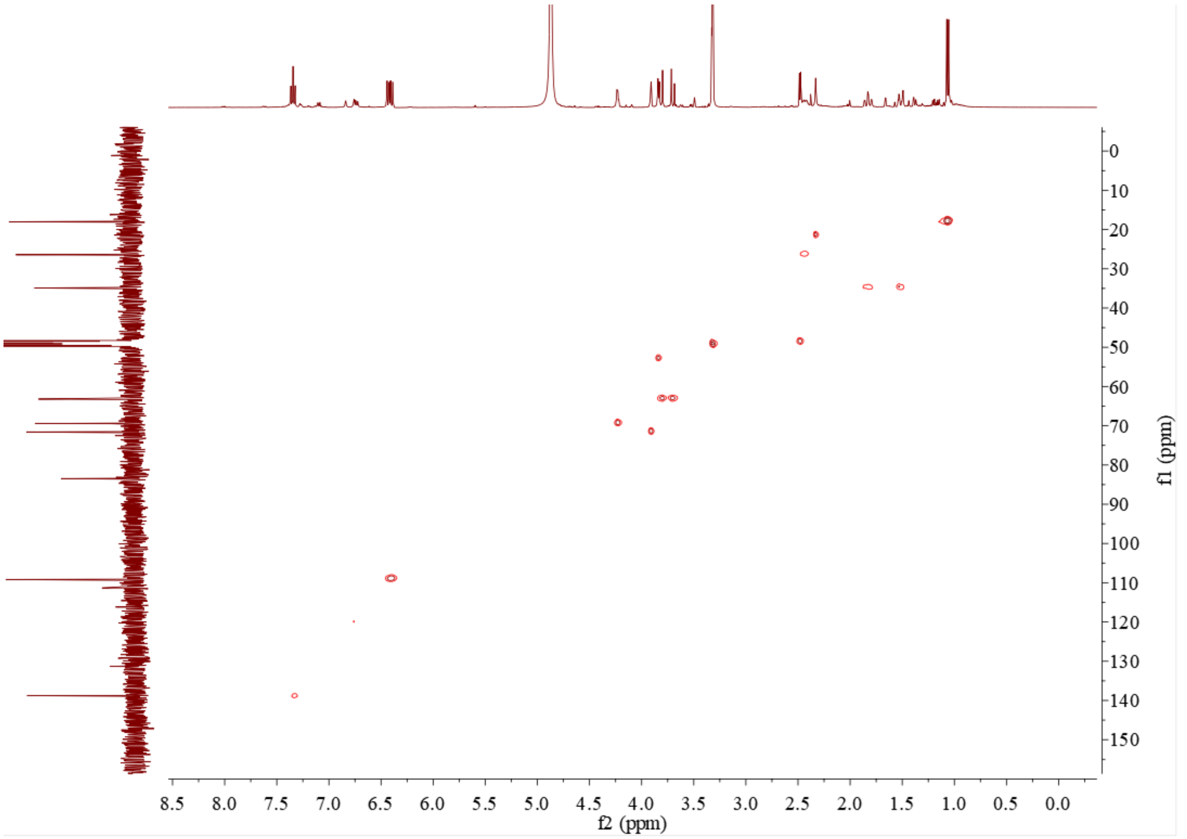

Figure S28. The HSQC spectrum of compound **4** in CD_3OD.

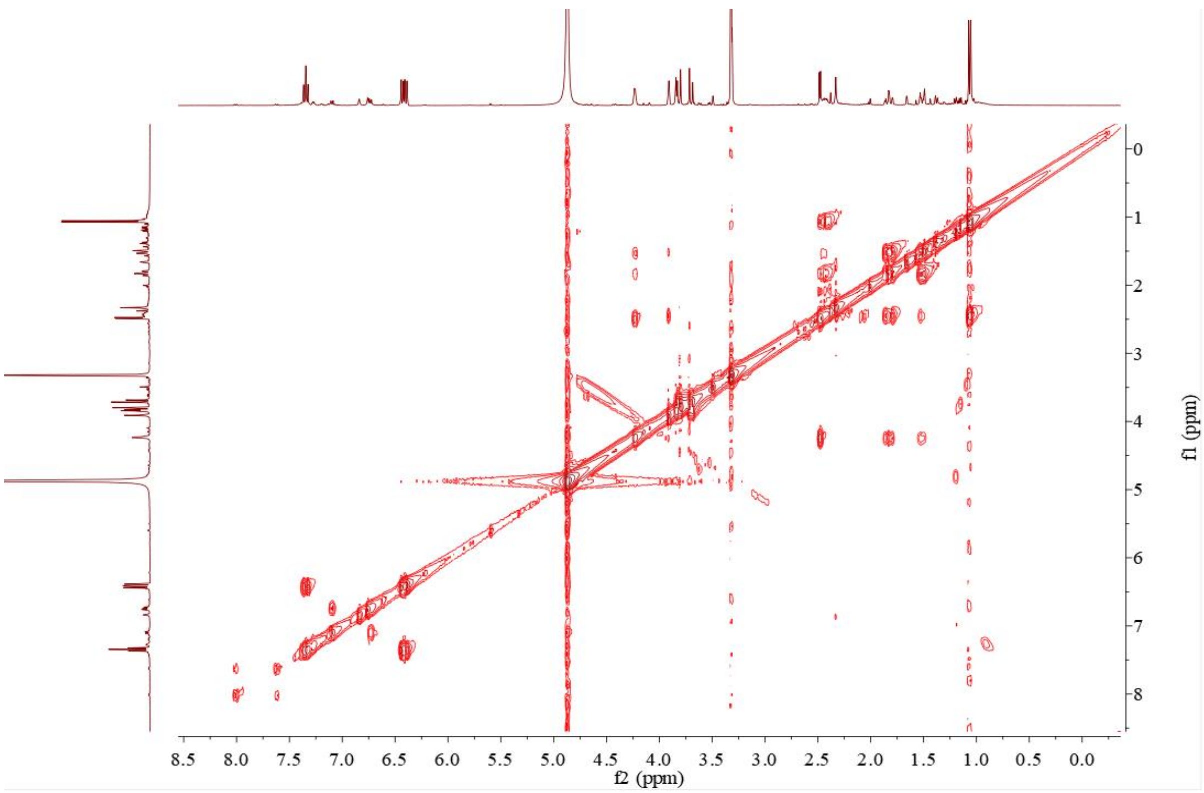

Figure S29. The ^{1}H-^{1}H COSY spectrum of compound **4** in CD_3OD.

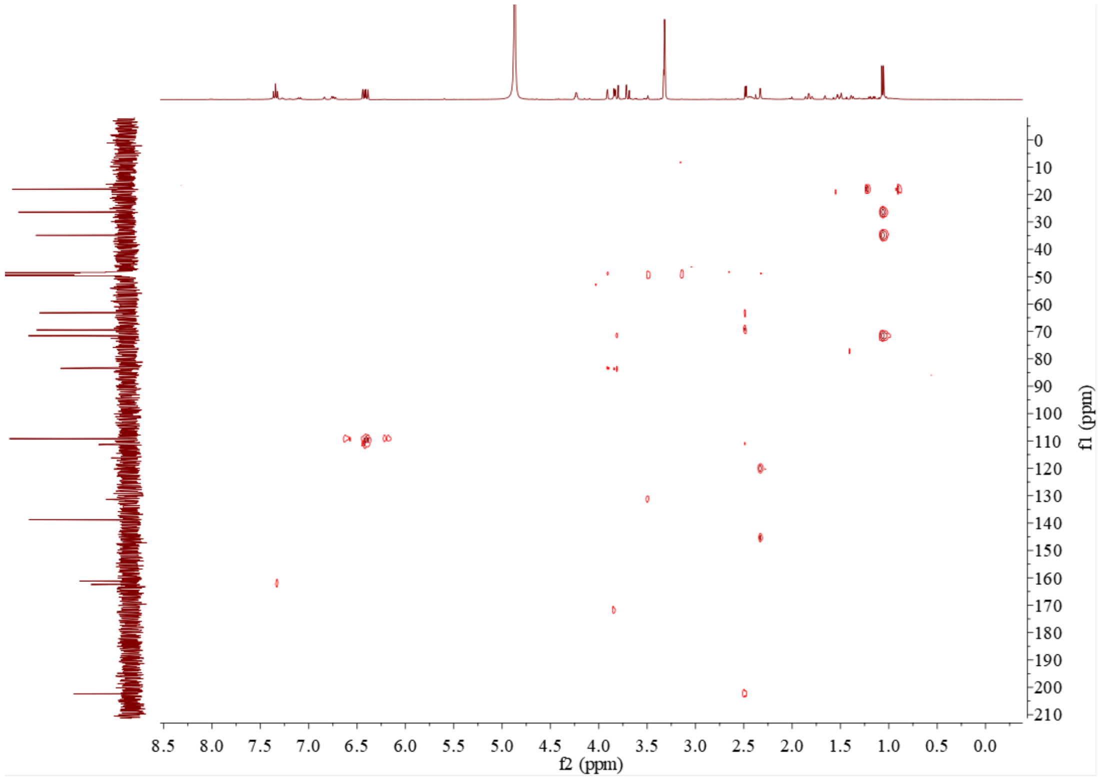

Figure S30. The HMBC spectrum of compound **4** in CD_3OD.

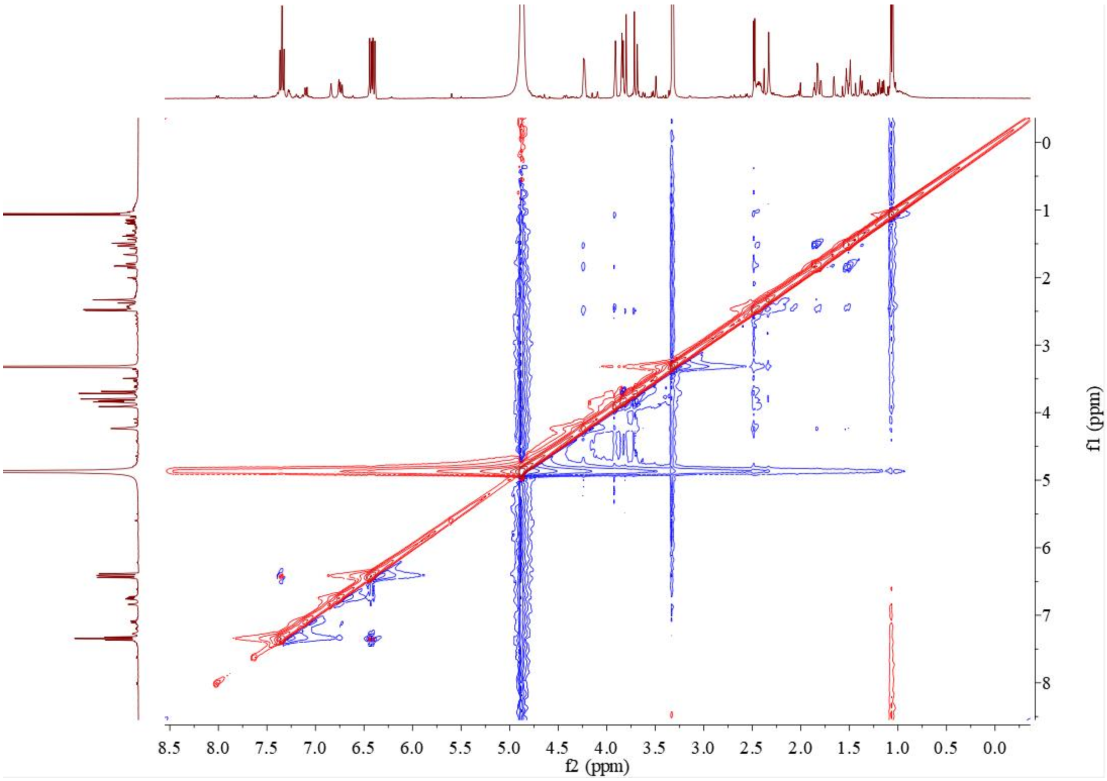

Figure S31. The NOESY spectrum of compound **4** in CD_3OD.

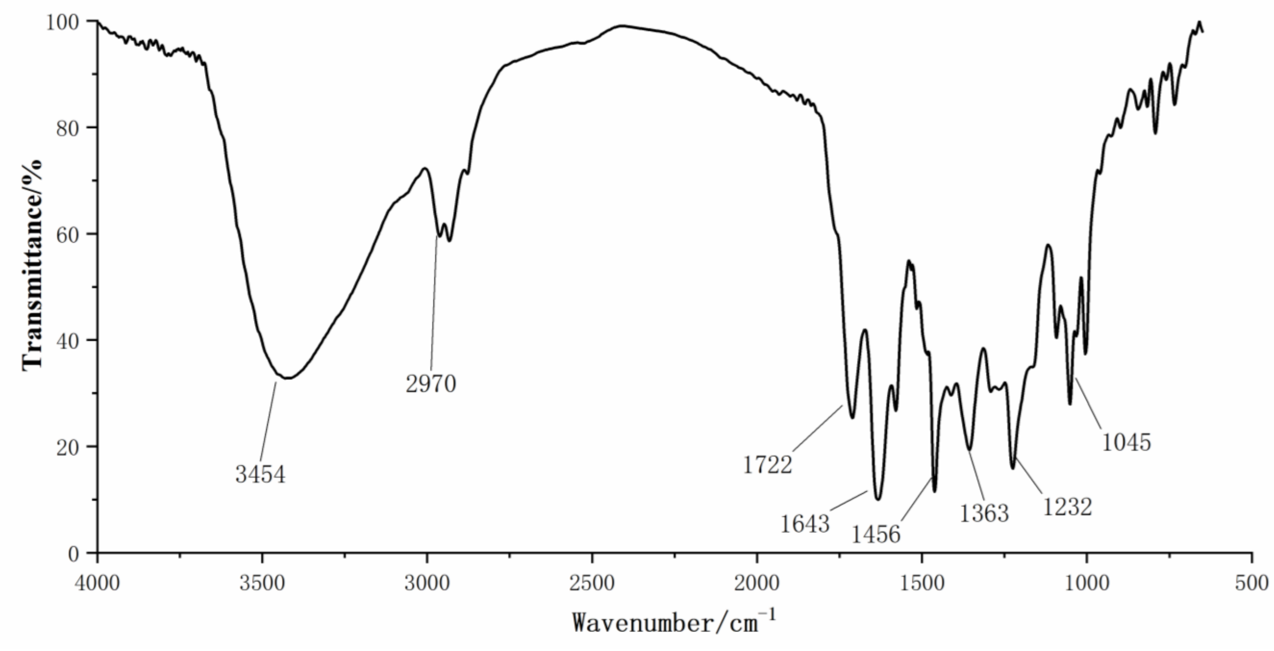

Figure S32. IR spectrum of compound **4**.

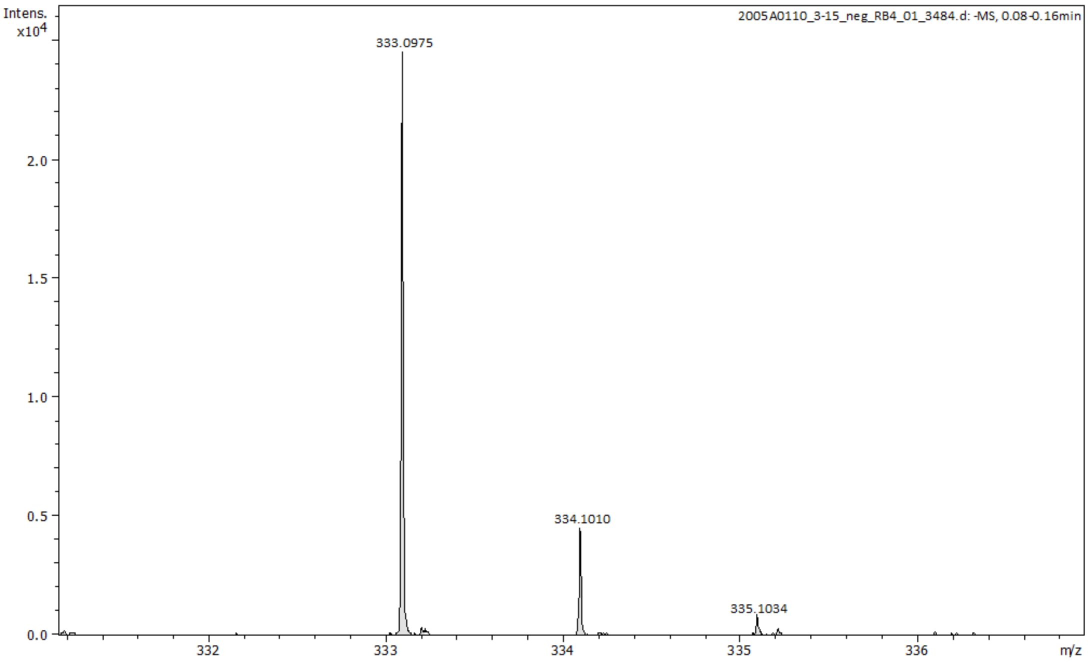

Meas. m/z	Ion Formula	m/z	z	err [ppm]	err [mDa]	mSigma	rdb	e⁻ Conf	N-Rule
333.0975	C17H17O7	333.0980	1-	1.6	0.5	3.4	9	even	ok

Figure S33. The HRESIMS spectrum of compound **5**.

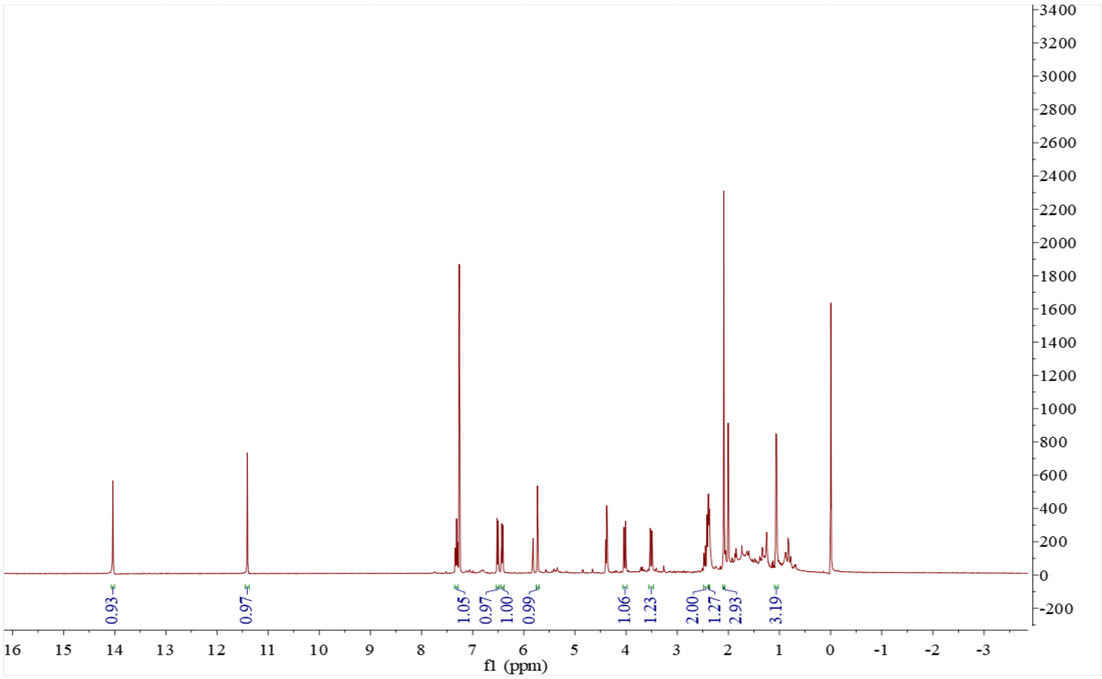

Figure S34. The ^{1}H NMR (400MHz) spectrum of compound **5** in $CDCl_3$.

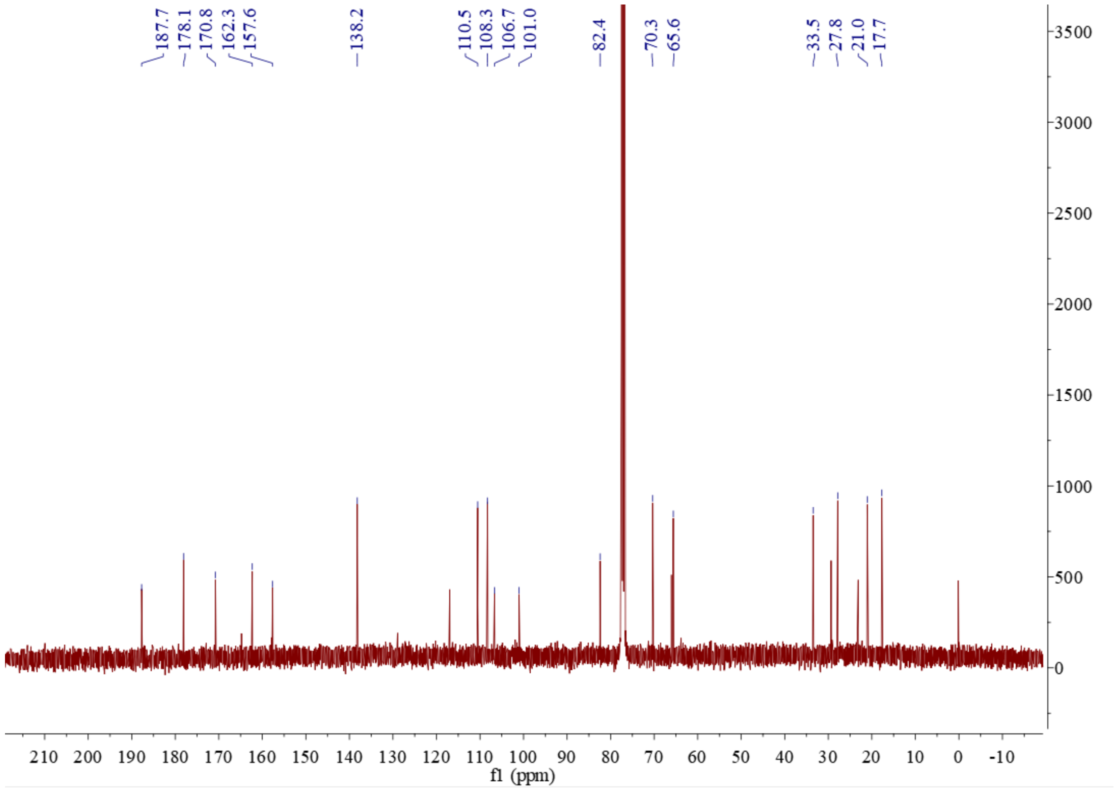

Figure S35. The ^{13}C NMR (100MHz) spectrum of compound **5** in $CDCl_3$.

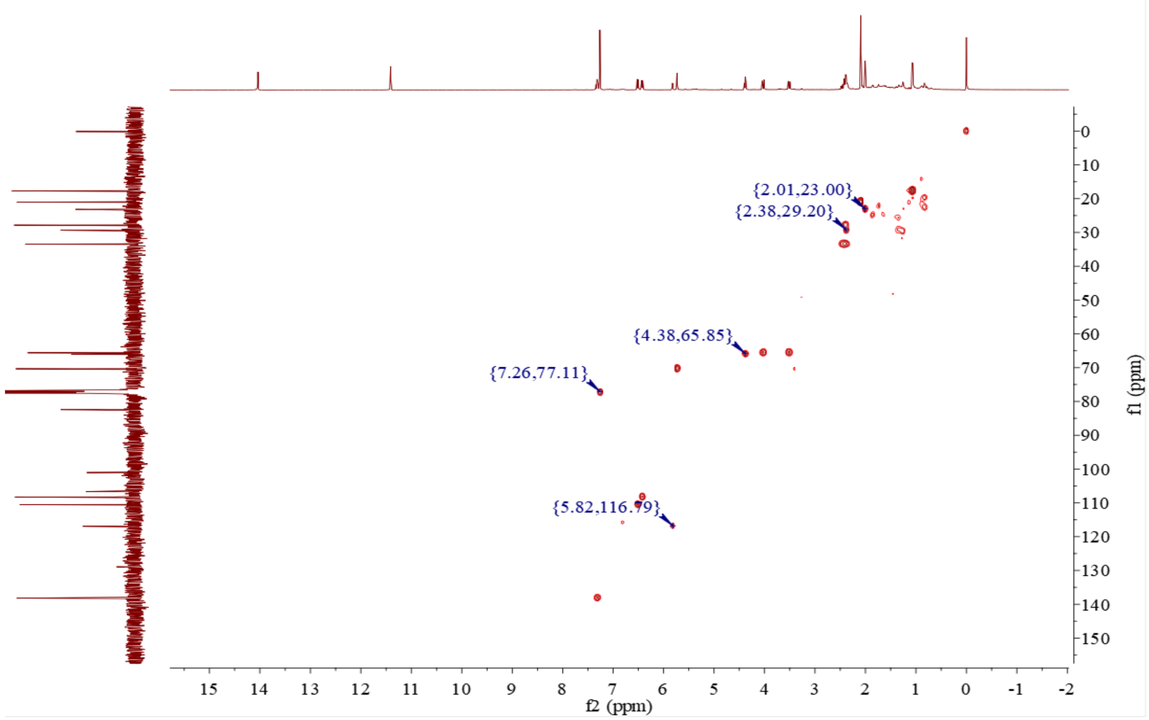

Figure S36. The HSQC spectrum of compound **5** in $CDCl_3$.

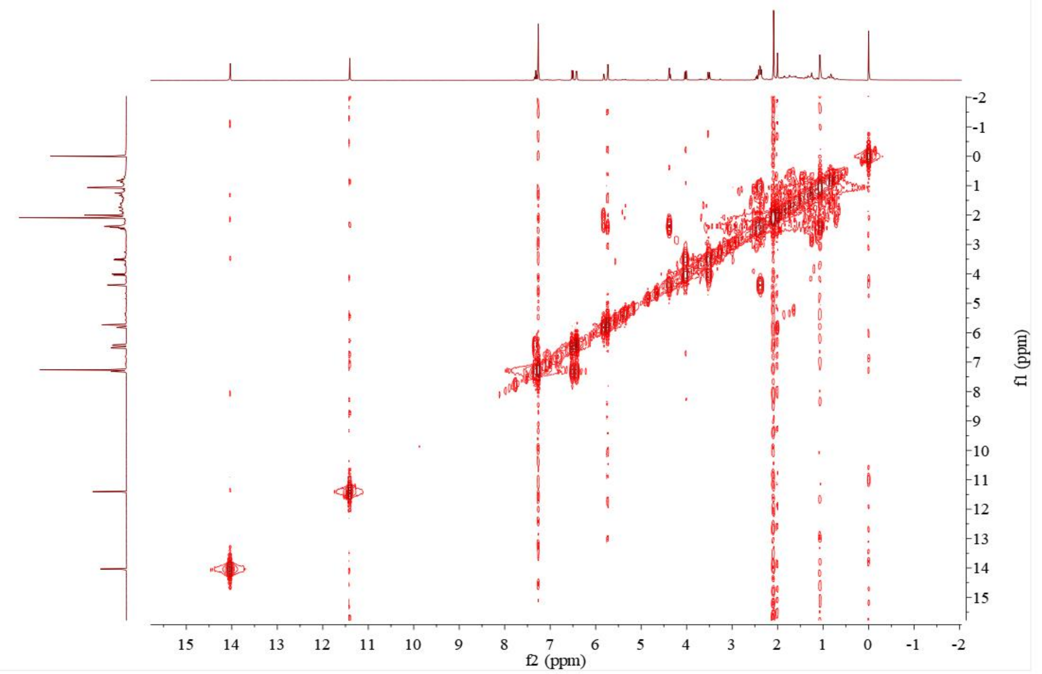

Figure S37. The ^{1}H-^{1}H COSY spectrum of compound **5** in $CDCl_3$.

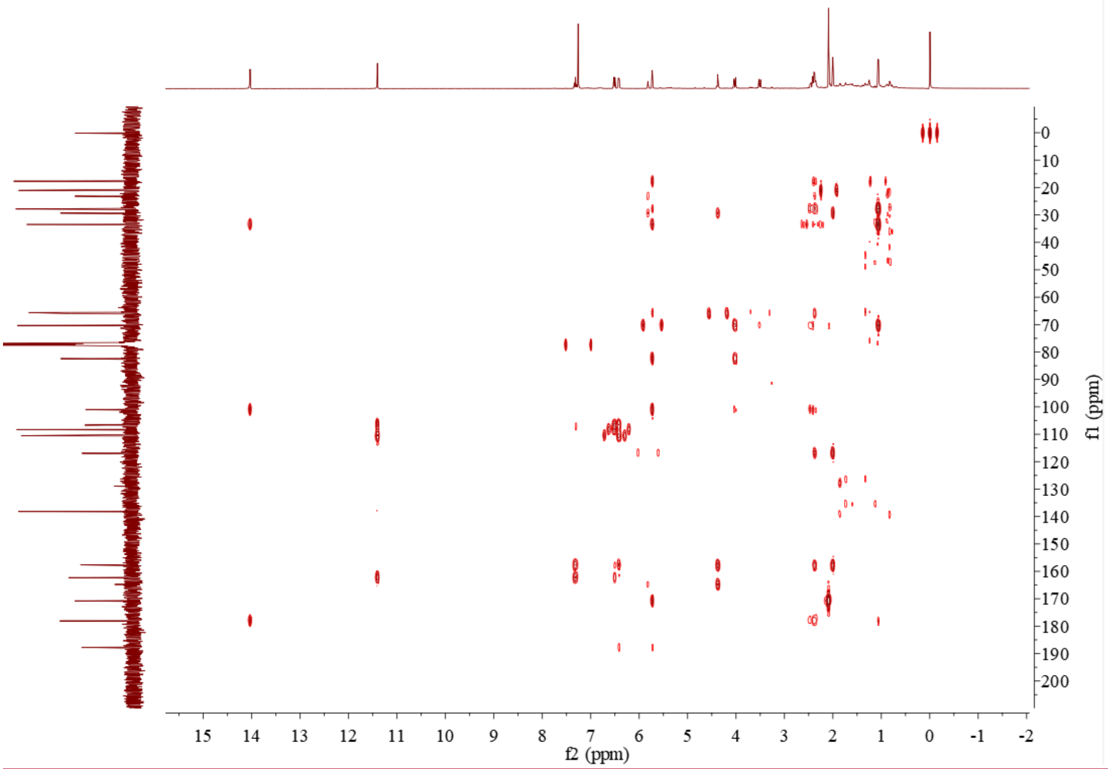

Figure S38. The HMBC spectrum of compound **5** in $CDCl_3$.

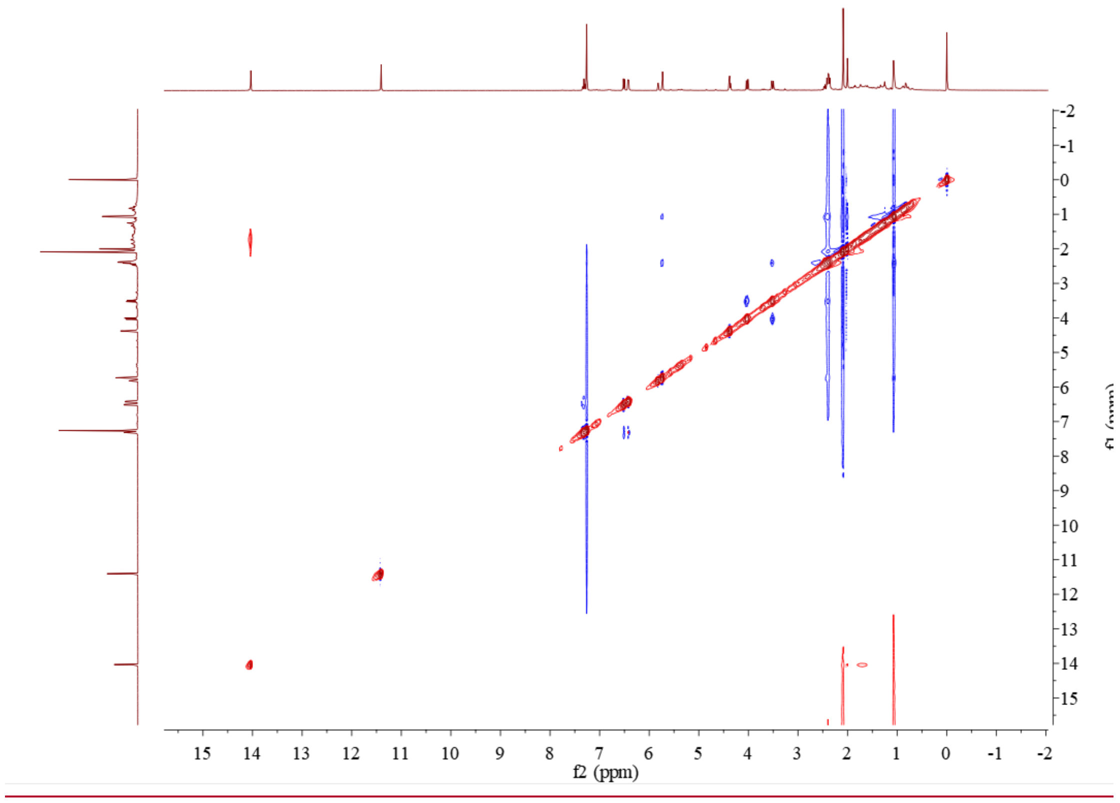

Figure S39. The NOESY spectrum of compound **5** in $CDCl_3$.

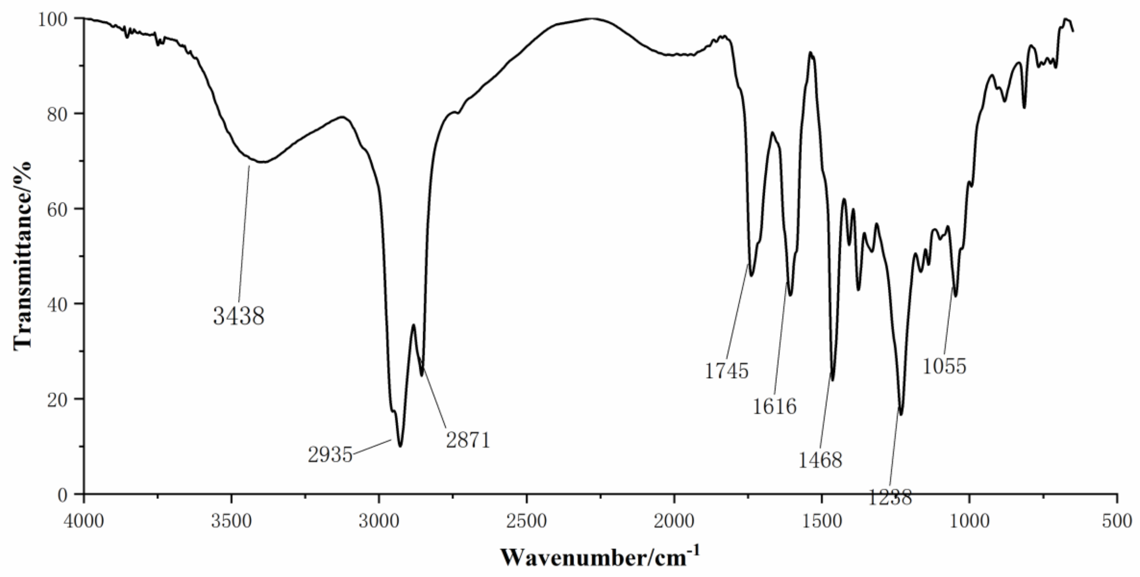

Figure S40. IR spectrum of compound **5**.

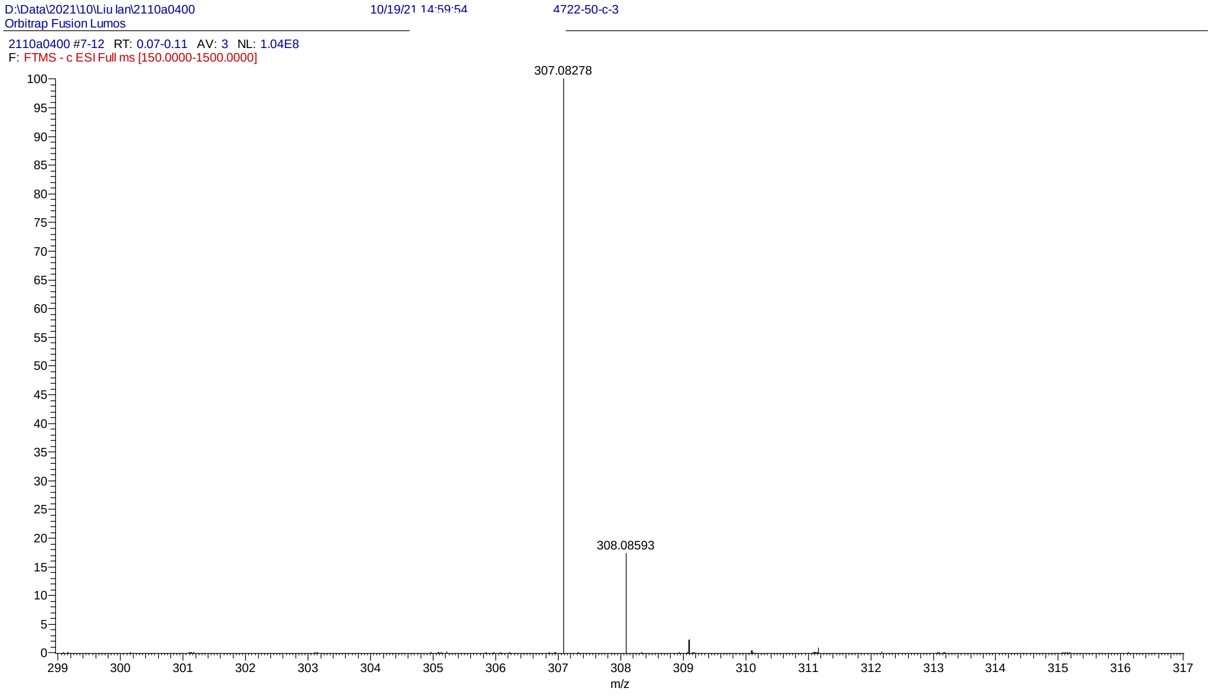

SPECTRUM - simulation :

m/z	Theo. Mass	Delta (ppm)	RDB equiv.	Composition
307.08278	307.08233	1.48	8.5	C15 H15 O7

Figure S41. The HRESIMS spectrum of compound **6**.

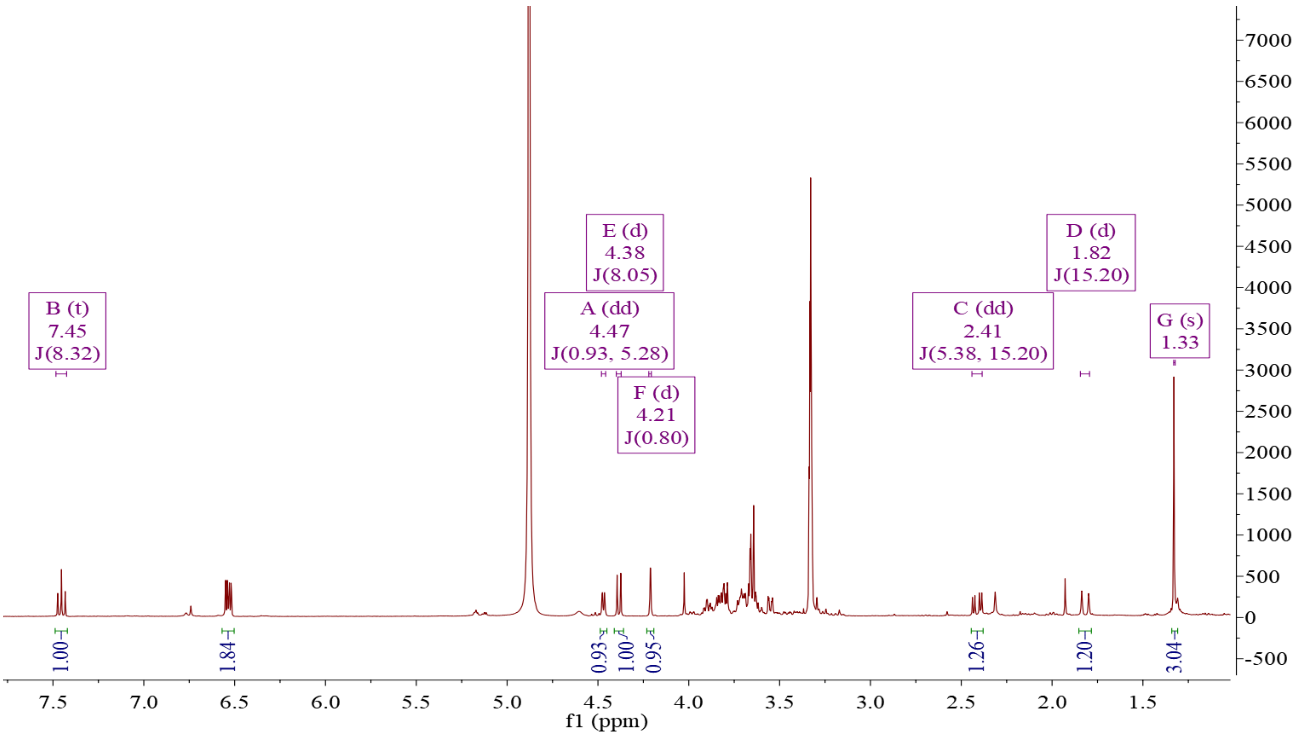

Figure S42 The 1H NMR (400MHz) spectrum of compound **6** in CD_3OD.

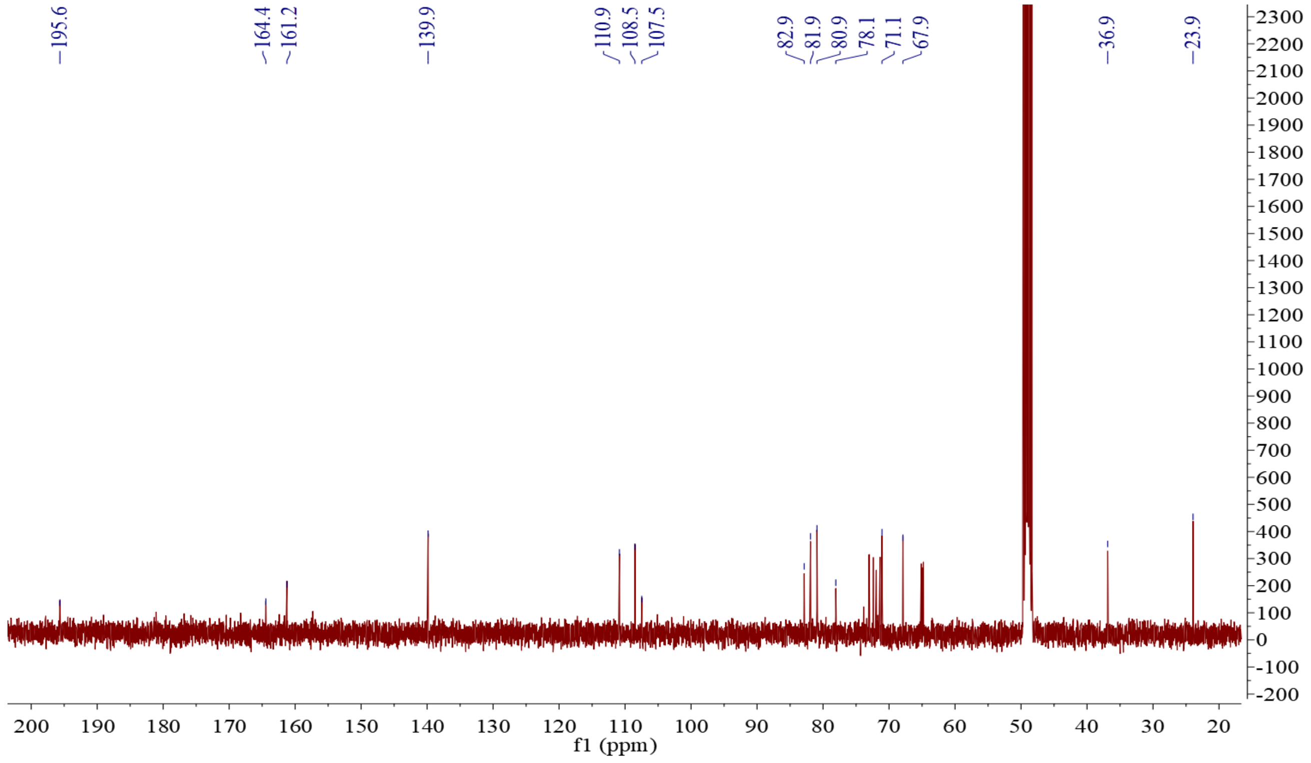

Figure S43. The 13C NMR (100MHz) spectrum of compound **6** in CD_3OD

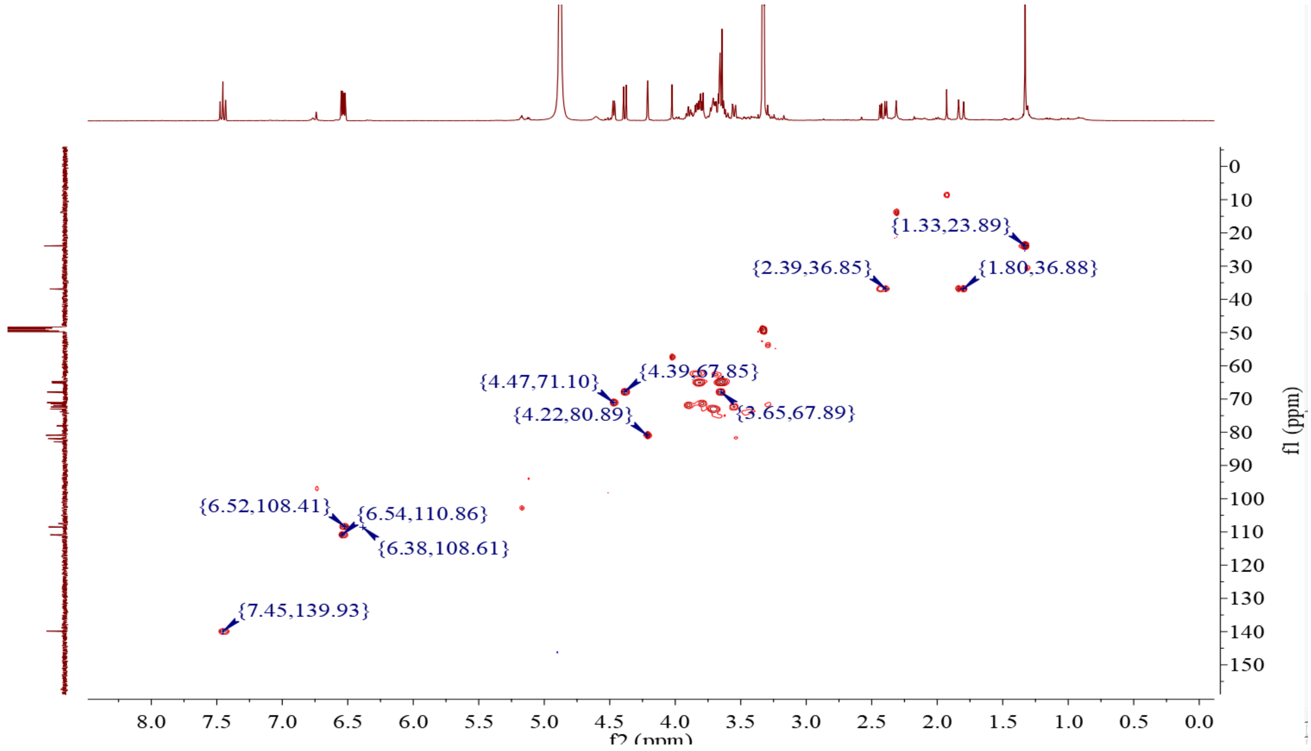

Figure S44. The HSQC spectrum of compound **6** in $CDCl_3$.

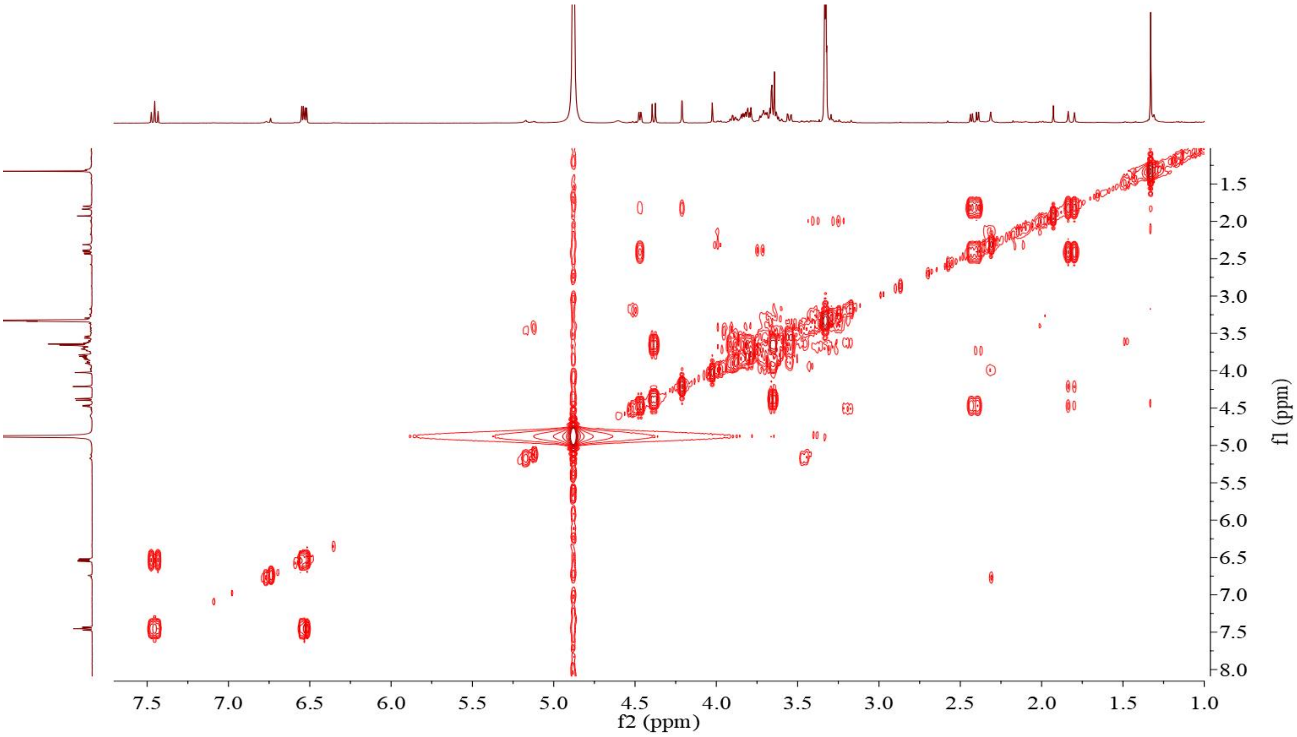

Figure S45. The ^{1}H-^{1}H COSY spectrum of compound **6** in $CDCl_3$.

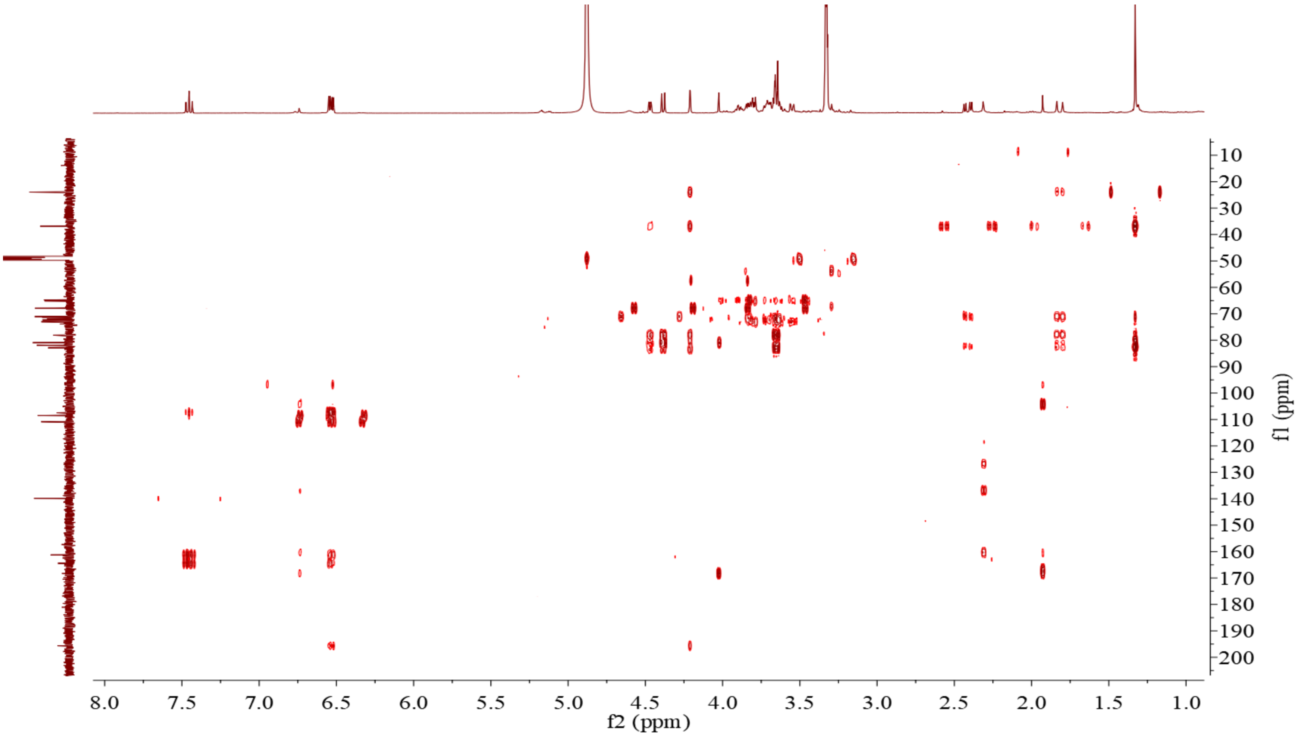

Figure S46. The HMBC spectrum of compound **6** in $CDCl_3$.

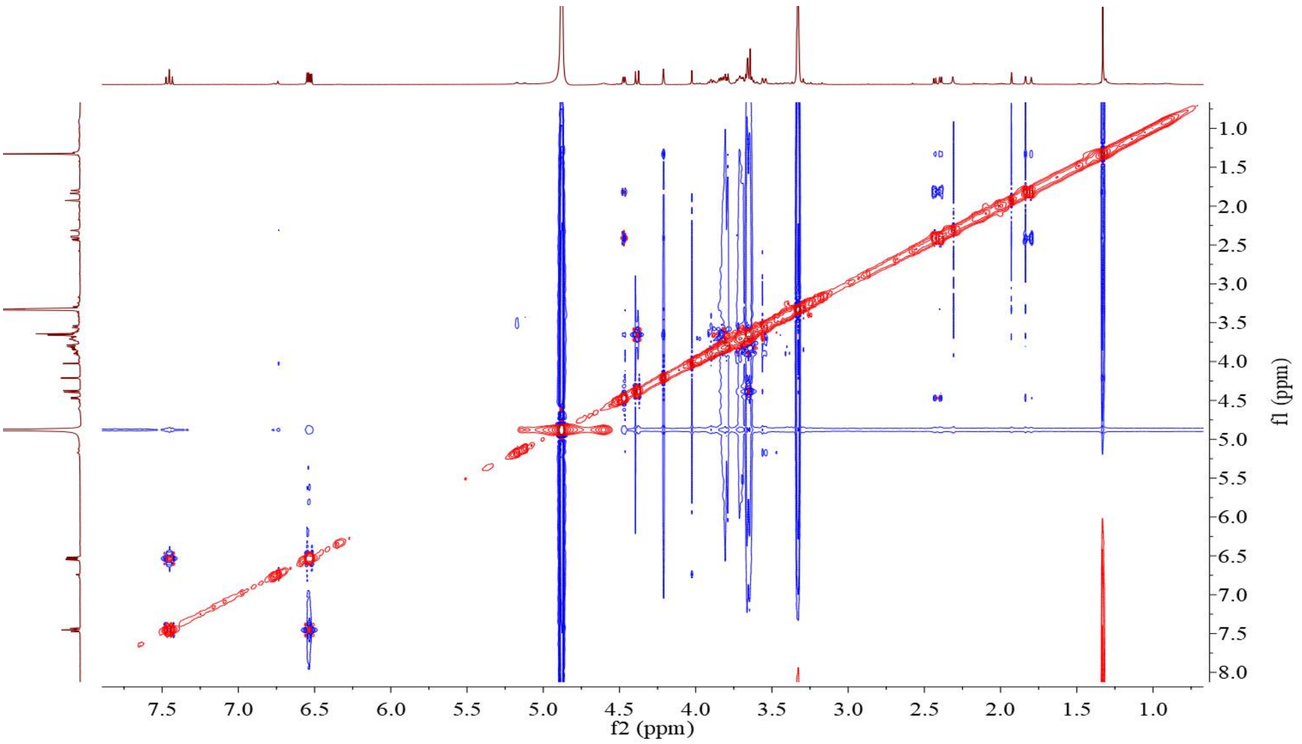

Figure S47. The NOESY spectrum of compound **6** in $CDCl_3$.

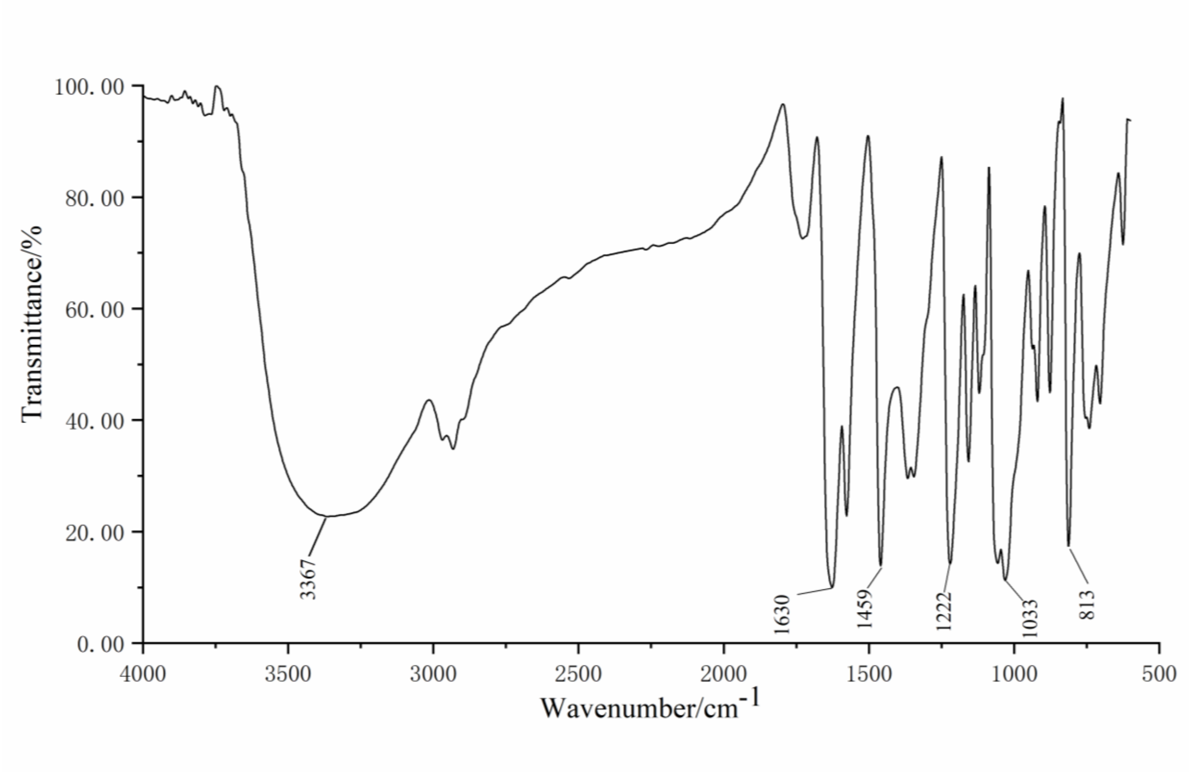

Figure S48. IR spectrum of compound **6**.

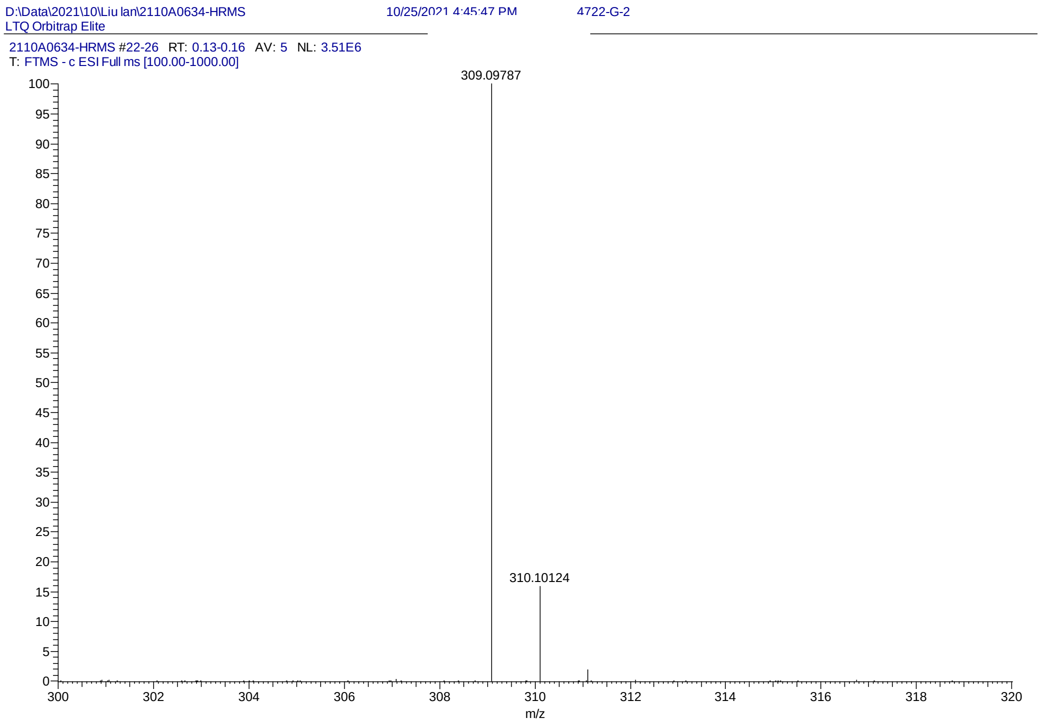

SPECTRUM - simulation :

m/z	Theo. Mass	Delta (ppm)	RDB equiv.	Composition
309.09787	309.09798	-0.34	7.5	C15 H17 O7

Figure S49. The HRESIMS spectrum of compound **7** CD_3OD.

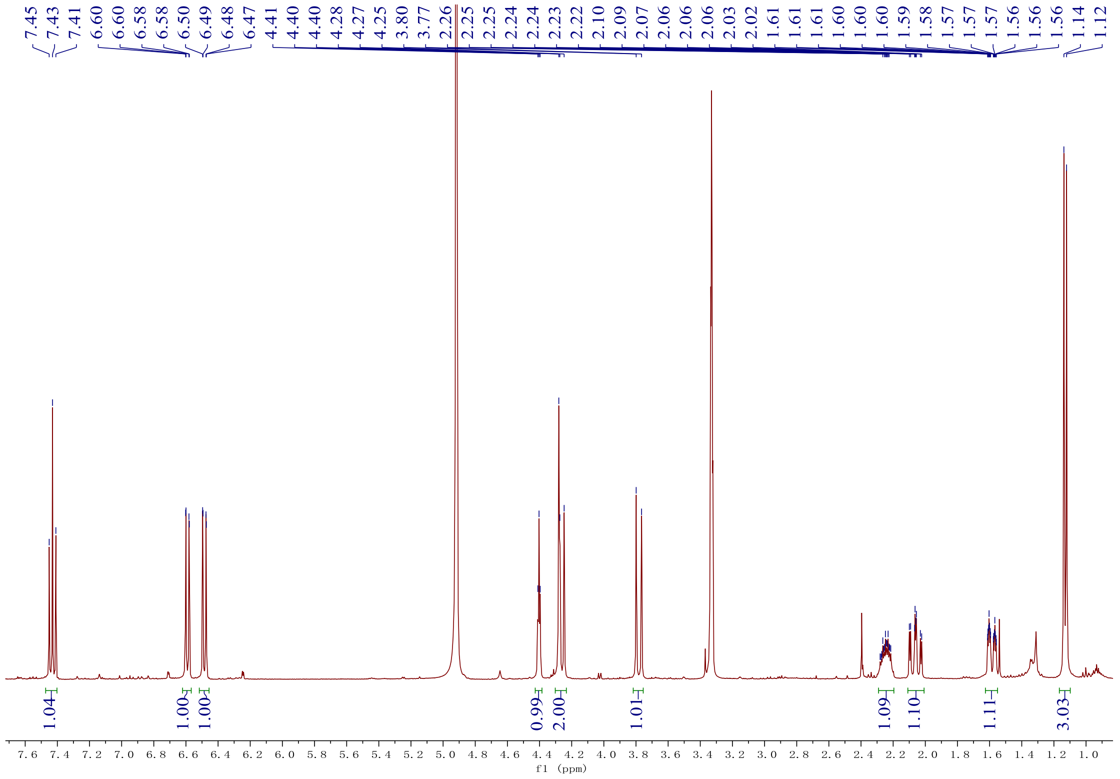

Figure S50. The ^1H NMR (400MHz) spectrum of compound **7** in CD_3OD.

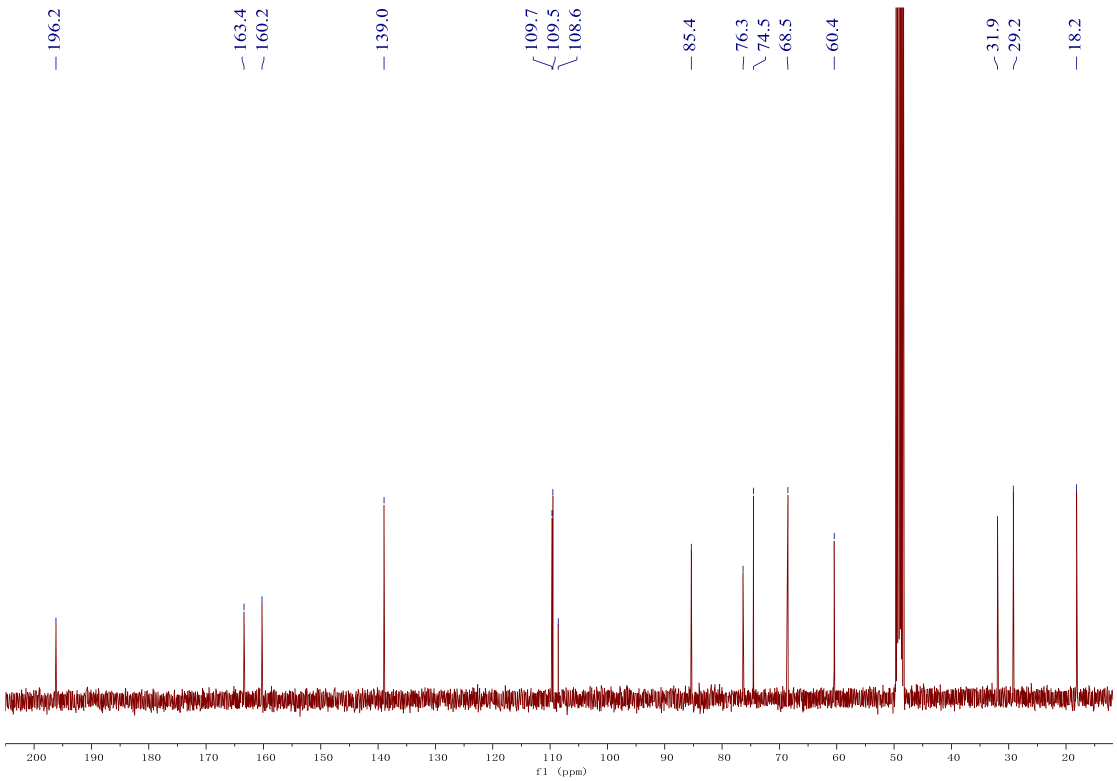

Figure S51. The ^{13}C NMR (100MHz) spectrum of compound **7** in CD_3OD.

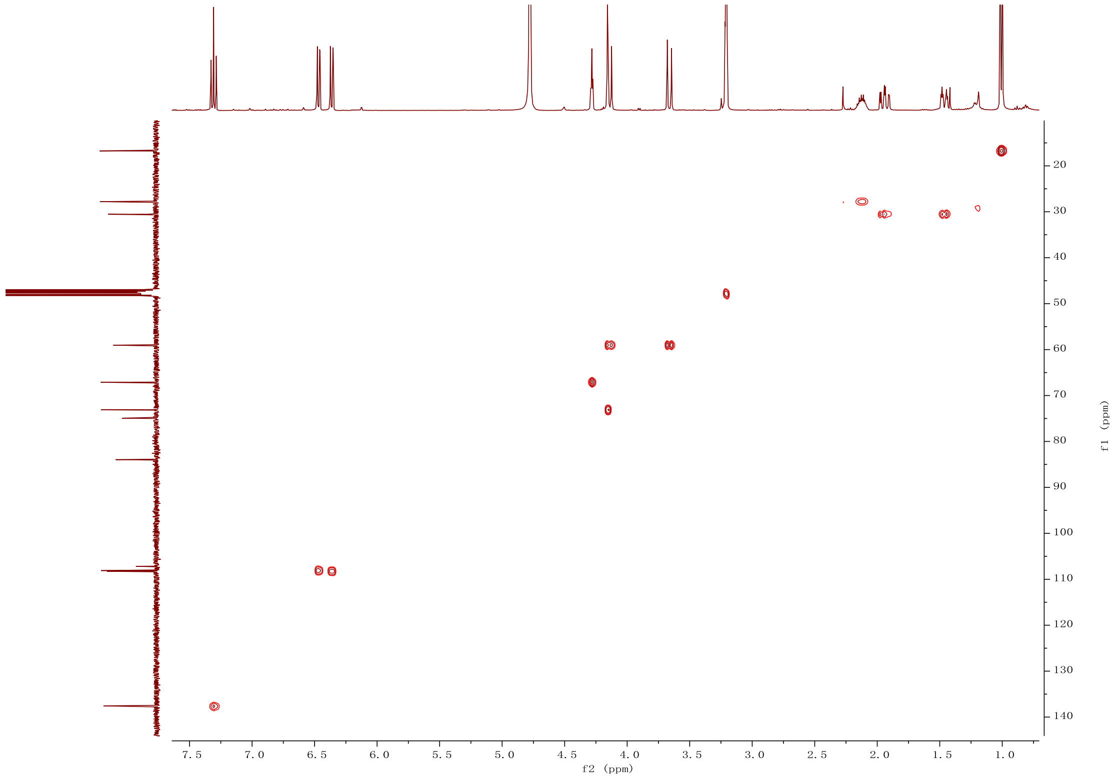

Figure S52. The HSQC spectrum of compound **7** in CD_3OD.

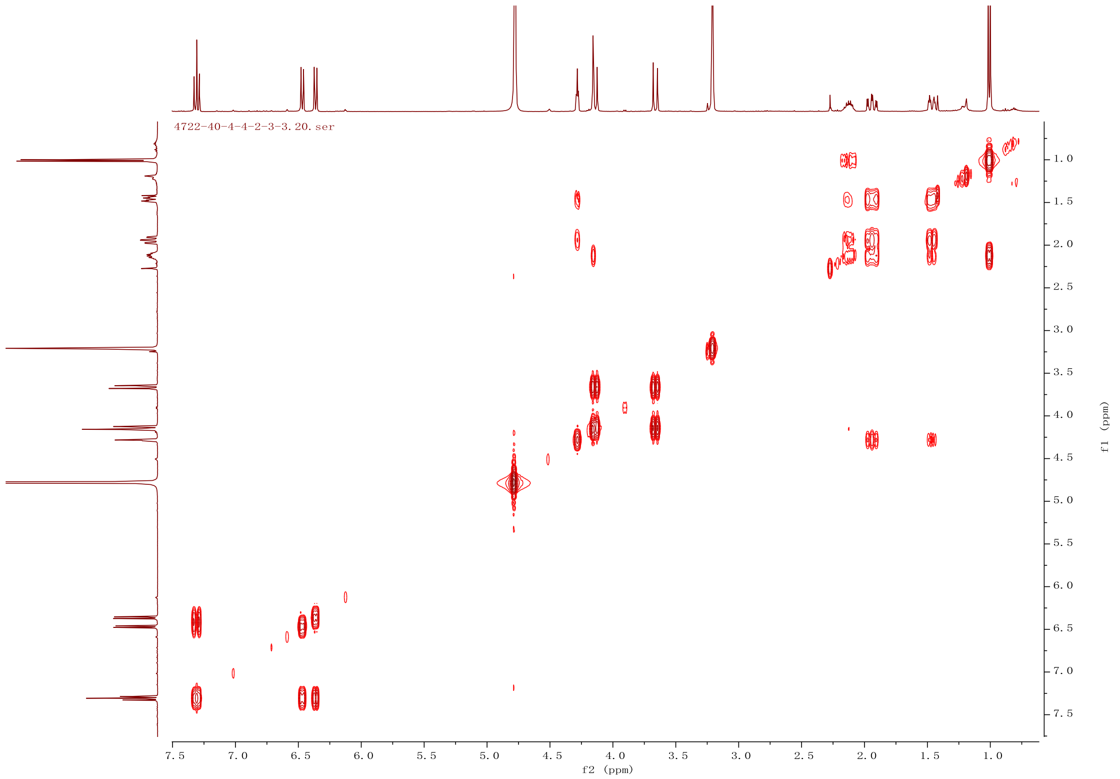

Figure S53. The ¹H-¹H COSY spectrum of compound **7** in CD_3OD.

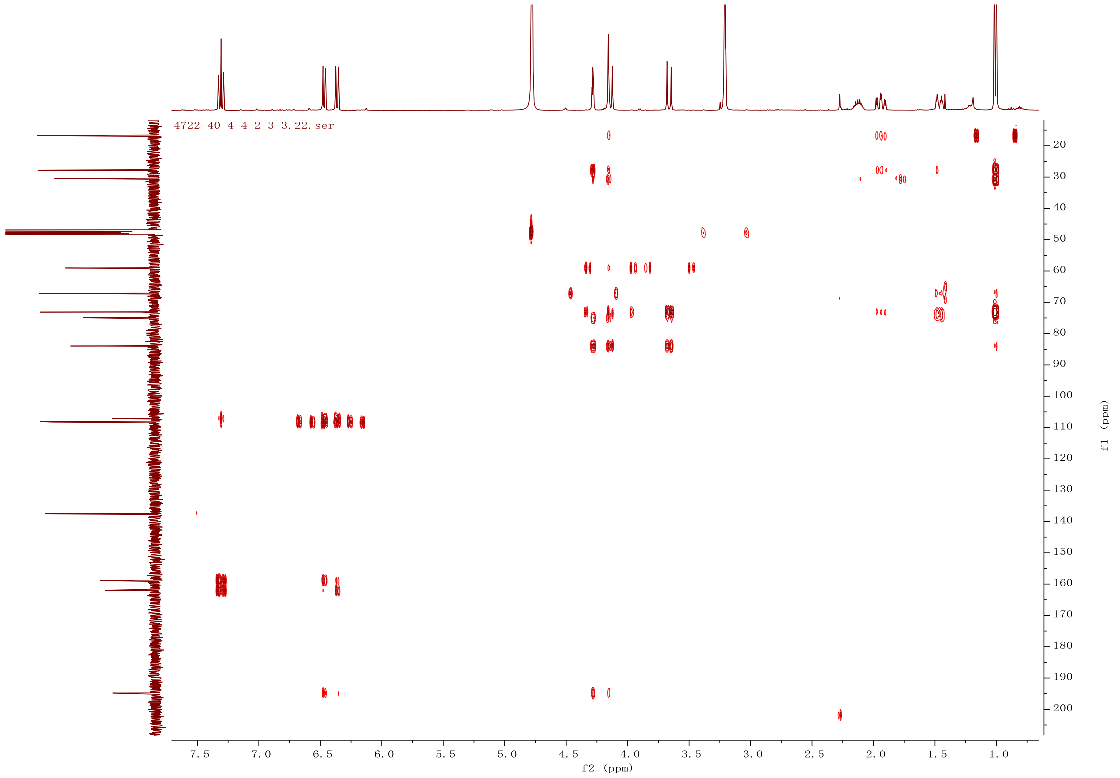

Figure S54. The HMBC spectrum of compound **7** in CD_3OD.

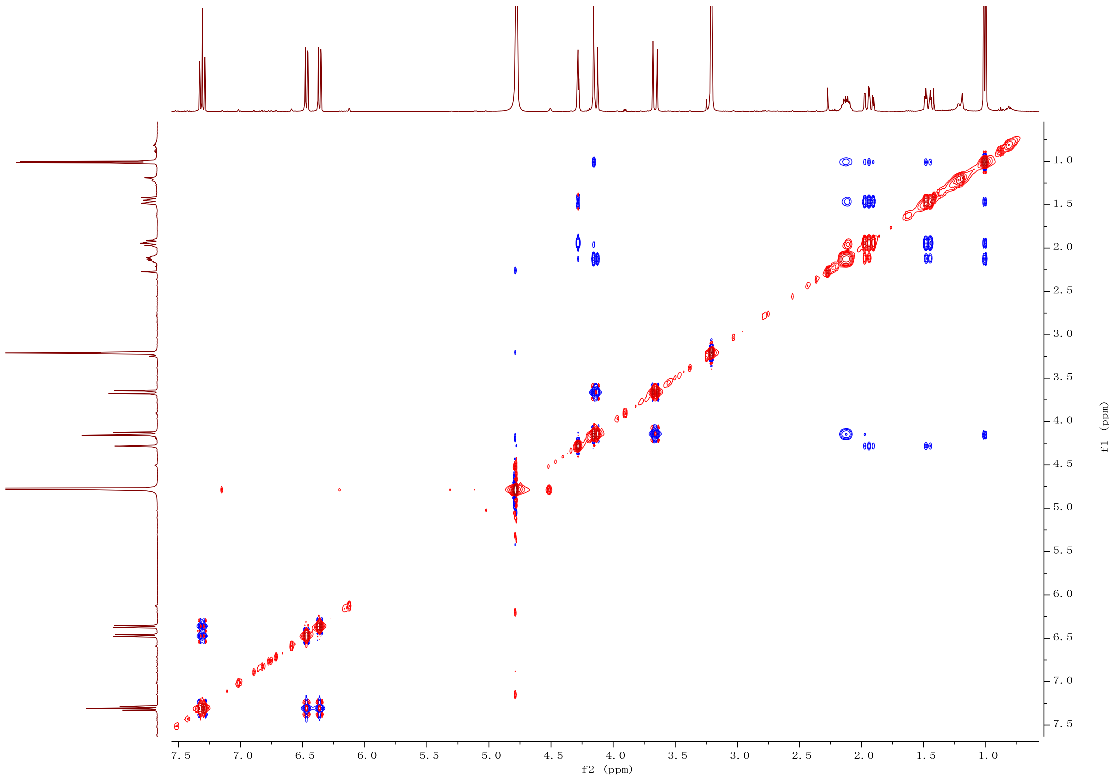

Figure S55. The NOESY spectrum of compound **7** in CD_3OD.

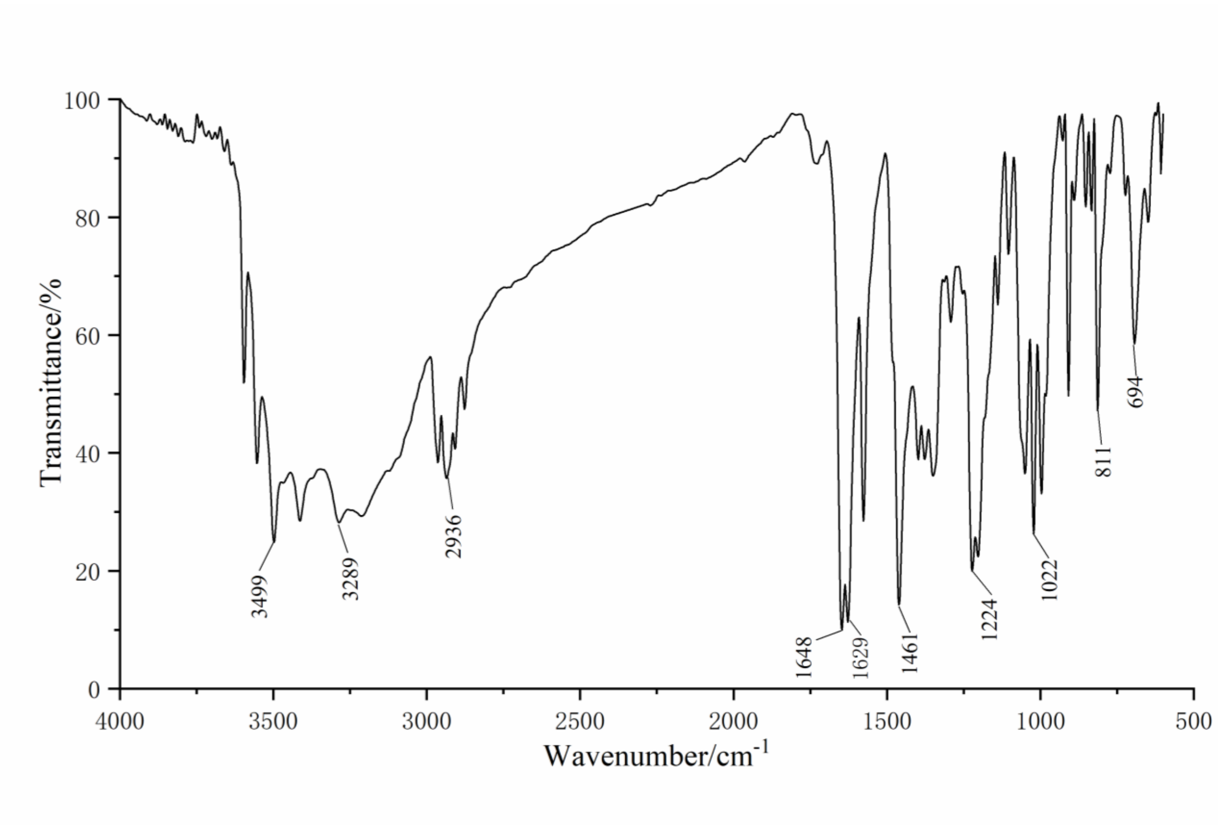

Figure S56. IR spectrum of compound **7**.

Experimental section:

Merck molecular force field (MMFF) and DFT/TD-DFT calculations were carried out with the Spartan'14 software package (Wavefunction Inc., Irvine, CA, USA) and the Gaussian 09 program, respectively. MMFF conformational search generated low-energy conformers within a 10 kcal·mol^{-1} energy window and optimized using PM6 semi-empirical optimizations. And then each conformer was optimized with HF/6-31G(d) method in Gaussian09. Further optimization was performed at the b3lyp/6-311g** level. The frequency was calculated at the same level to confirm each optimized conformer with true minimum and to estimate their relative thermal free energies (ΔG) at 298.15 K. The optimized conformers were continually used for the ECD calculations in methanol, which were carried out with Gaussian09 (b3lyp/6-311g**). Solvent effects were taken into account by using the polarizable continuum model (PCM). The ECD data was generated by the program SpecDis using a Gaussian band shape with 0.30 eV exponential half-width from dipole-length dipolar and rotational strengths and the final ECD spectrum was drawn by Origin 2018. All calculations were performed by Tianhe-2 of the National Super Computer Center in Guangzhou.

Table S1. Energy of all conformers of diaporthone A (**1**).

compound	conformer	E (kcal/mol)	rel.E(kcal/mol)	Boltzmann dist (%)
	**1-a**	-648144.4421	0	59.38%
**1**	**1-b**	-648143.991	0.451097732	27.72%
	**1-c**	-648143.5385	0.903563435	12.90%

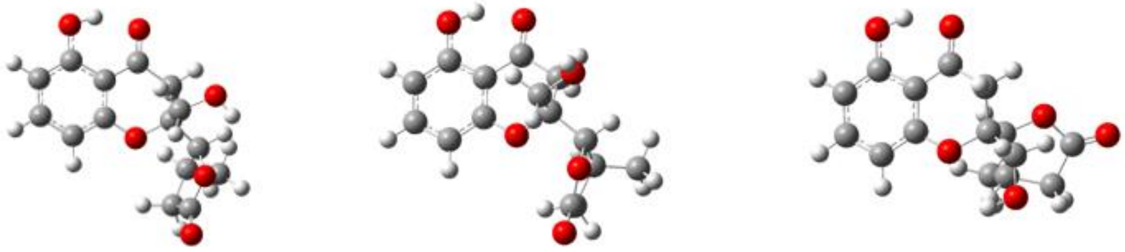

1-a **1-b** **1-c**

Figure S57. B3LYP/6-311g** optimized low-energy conformers of diaporthone A (**1**).

Table S2. Cartesian coordinates of the low-energy reoptimized conformers of **1** calculated at b3lyp/6-311g** level of theory in PCM for methanol.

**1-a**			
Atom	X axis (Å)	Y axis (Å)	Z axis (Å)
C	1.22981500	0.95812100	0.07502500
C	2.18791400	-0.05660900	-0.15790000
C	3.56604000	0.29718200	-0.20479100
C	3.95182500	1.62791700	-0.04271600

C	2.97716700	2.59786000	0.16600600
C	1.61963400	2.28295000	0.23040800
O	-0.10211400	0.69134500	0.13441300
C	-0.54844000	-0.67279900	0.34577800
C	0.27393000	-1.67296100	-0.47246300
C	1.76320700	-1.42189700	-0.40057900
O	2.56169200	-2.34648600	-0.59585900
O	4.50775300	-0.63475500	-0.41652100
C	-2.01965200	-0.71655200	-0.10543100
C	-0.42617400	-0.95595100	1.86363200
C	-2.33436300	-0.05952900	-1.47575300
C	-2.87678500	1.31896800	-1.06839300
C	-3.32351700	1.15584100	0.36750800
O	-2.84634400	-0.02006600	0.86270100
O	-0.97698000	-2.21961900	2.21127800
C	-3.35298300	-0.88055900	-2.27431600
O	-3.99136600	1.89172900	1.04268300
H	-2.33945100	-1.75859000	-0.08044000
H	5.00360600	1.88019000	-0.08334600
H	3.28145600	3.63069500	0.29209400
H	0.86916700	3.04121100	0.41010700
H	0.06774400	-2.68368900	-0.11737500
H	0.00197000	-1.63682800	-1.53317000
H	4.03951700	-1.49813200	-0.52156700
H	0.62878700	-0.98098700	2.14358100
H	-0.90356800	-0.14002400	2.40856800
H	-1.42095100	0.04918500	-2.06034600
H	-2.10434300	2.09075100	-1.08908600
H	-3.71227800	1.66340600	-1.67904600
H	-1.92130000	-2.10349400	2.36256400
H	-3.60099300	-0.37506800	-3.21067700
H	-4.28031400	-1.01842200	-1.70979400
H	-2.95589100	-1.86929500	-2.51772000

1-b

Atom	X axis (Å)	Y axis (Å)	Z axis (Å)
C	1.20551300	0.96442100	0.07673700
C	2.16409500	-0.04489000	-0.17522900
C	3.53927800	0.31495800	-0.24412500

C	3.92163900	1.64719300	-0.08551000
C	2.94623100	2.61212100	0.14181200
C	1.59131400	2.29064100	0.22860700
O	-0.12393400	0.69003700	0.16173700
C	-0.55756400	-0.67642600	0.37752000
C	0.25110300	-1.66518700	-0.47011200
C	1.74032000	-1.41141800	-0.41493900
O	2.53956700	-2.33249900	-0.62329500
O	4.48153100	-0.61264100	-0.47321400
C	-2.04432800	-0.71179900	-0.02515900
C	-0.39189000	-0.97208500	1.88017000
C	-2.38415800	-0.15489600	-1.43497700
C	-2.88854300	1.26295000	-1.12752600
C	-3.27531400	1.23301900	0.33383700
O	-2.81426200	0.08975700	0.90682300
O	-0.86869400	-2.29390900	2.10991000
C	-3.43526400	-1.01723800	-2.14246400
O	-3.89264800	2.04549500	0.96998100
H	-2.38900200	-1.73745900	0.10309200
H	4.97143900	1.90447800	-0.14276000
H	3.24790900	3.64598200	0.26568800
H	0.84055400	3.04471900	0.42400200
H	0.05374200	-2.68217600	-0.13162200
H	-0.03440000	-1.60680500	-1.52565300
H	4.01495400	-1.47782800	-0.57089800
H	0.66498100	-0.87856500	2.15581500
H	-0.96593600	-0.23218900	2.44283200
H	-1.48639900	-0.10887500	-2.05119600
H	-2.10781200	2.01706900	-1.24829500
H	-3.74448200	1.56852700	-1.73078200
H	-0.82447000	-2.46880400	3.05540300
H	-3.69756300	-0.58368500	-3.11059000
H	-4.35072800	-1.09327100	-1.54765900
H	-3.05987400	-2.02922400	-2.31475500

**1-c**			
Atom	X axis (Å)	Y axis (Å)	Z axis (Å)

C	-1.53938200	-0.84720400	0.53670600
C	-2.11198400	0.36795700	0.09371000
C	-3.39749400	0.33993600	-0.51621400
C	-4.08042600	-0.86935200	-0.65076700
C	-3.49148000	-2.03878000	-0.18296000
C	-2.22693300	-2.04739800	0.40737000
O	-0.30929100	-0.89434500	1.11881700
C	0.60351000	0.22441700	0.96466500
C	-0.13777500	1.55993400	1.11057100
C	-1.42185900	1.62464900	0.31617200
O	-1.87035800	2.71184400	-0.06628300
O	-3.97416800	1.46824500	-0.95668700
C	1.23356500	0.11455300	-0.45440600
C	1.59491200	0.07173400	2.12959500
C	1.95984600	-1.20251400	-0.84138500
C	3.44110200	-0.82851200	-0.69463800
C	3.47477200	0.68137400	-0.75818700
O	2.21104600	1.17436700	-0.60172700
O	2.22203200	-1.19812800	2.19522200
C	1.60139100	-1.63363600	-2.26863800
O	4.41279500	1.41741400	-0.90514700
H	0.44951900	0.32996500	-1.18310400
H	-5.05840200	-0.87318800	-1.11452800
H	-4.02603600	-2.97590400	-0.28853000
H	-1.76922100	-2.96403700	0.75487700
H	-0.41278600	1.71782400	2.16008700
H	0.50838300	2.38623500	0.81324900
H	-3.35009000	2.20746300	-0.75869400
H	2.38607500	0.81584100	2.03018200
H	1.04968200	0.28134600	3.05668700
H	1.68968000	-1.99385300	-0.14708900
H	3.83520200	-1.12659000	0.27992000
H	4.08690900	-1.24862200	-1.46617400
H	1.52918200	-1.85430000	2.33585000
H	2.16012700	-2.53028800	-2.54802400
H	1.83907800	-0.84927700	-2.99423800
H	0.53566200	-1.86073300	-2.35360600

Table S3. Energy of all conformers of diaporthone C (**3**).

compound	conformer	E (kcal/mol)	rel.E(kcal/mol)	Boltzmann dist (%)
**3**	**3-a**	-695330.4661	0	52.06%
	**3-b**	-695330.5150	-0.048914363	47.94%

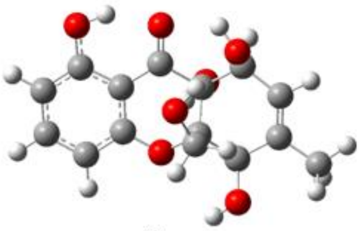

3-a

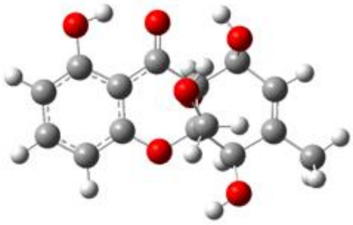

3-b

Figure S58. B3LYP/6-311g** optimized low-energy conformers of diaporthone C (**3**).

Table S4. Cartesian coordinates of the low-energy reoptimized conformers of **3** calculated at b3lyp/6-311g** level of theory in PCM for methanol.

**3-a**			
Atom	X axis (Å)	Y axis (Å)	Z axis (Å)
C	-1.51403000	-1.24687700	-0.11241800
C	-1.91565900	0.11072500	-0.15131000
C	-3.30190400	0.42238600	-0.12927800
C	-4.24829500	-0.60172800	-0.10023900
C	-3.81699300	-1.92409900	-0.08694200
C	-2.46359200	-2.26219200	-0.08762700
O	-0.20897700	-1.61769600	-0.10863500
C	0.81721500	-0.62588100	0.18532000
C	0.50139300	0.67338600	-0.56812500
C	-0.92025100	1.15461100	-0.25091400
C	2.12379600	-1.21228200	-0.38161900
C	3.23505200	-0.17789100	-0.37528700
C	2.96933000	1.13303500	-0.36210000
C	1.59630400	1.74045100	-0.33945200
O	-1.18222700	2.36505500	-0.23100200
O	1.42433800	2.46638400	0.89511000
C	4.63973200	-0.71025100	-0.44173300

O	2.53925900	-2.35134600	0.37127800
C	0.93209600	-0.46446300	1.72279700
O	0.03863000	0.48488700	2.28772000
O	-3.72619100	1.69827600	-0.14668800
O	0.47619900	0.32407500	-1.96096600
H	-5.30027000	-0.34747100	-0.08732100
H	-4.55487400	-2.71793100	-0.06237600
H	-2.13673800	-3.29322600	-0.05774500
H	1.93157800	-1.50628900	-1.41946100
H	3.78696600	1.84704100	-0.38228700
H	1.51333300	2.45936500	-1.16311600
H	0.69321700	3.08198700	0.74147000
H	5.36436100	0.10405700	-0.48171500
H	4.77339900	-1.33948200	-1.32838100
H	4.85779500	-1.33979100	0.42386300
H	1.90234500	-3.05451000	0.20034600
H	1.96588700	-0.21957600	1.97678000
H	0.70080400	-1.43395900	2.16202400
H	0.44813000	1.35056700	2.12766100
H	-2.93141200	2.27635600	-0.15835800
H	0.29206900	1.12221100	-2.47235300

3-b

Atom	X axis (Å)	Y axis (Å)	Z axis (Å)
C	-1.51648800	-1.24609200	-0.15051700
C	-1.91214600	0.11336000	-0.17410700
C	-3.29563600	0.42768800	-0.10324600
C	-4.24177600	-0.59691000	-0.03966500
C	-3.81457900	-1.91919500	-0.03858900
C	-2.46100500	-2.26022400	-0.08763000
O	-0.20273300	-1.61108200	-0.23225400
C	0.81484800	-0.63278200	0.15176200
C	0.51169100	0.67681900	-0.59274700
C	-0.91315000	1.15924200	-0.27841200
C	2.14194100	-1.21053500	-0.37124300
C	3.24745200	-0.17196700	-0.31732000
C	2.97442200	1.13725500	-0.32183600
C	1.60144000	1.74311800	-0.35415600
O	-1.18005100	2.36648600	-0.24770800

O	1.39837700	2.48890900	0.86175000
C	4.65610200	-0.69812000	-0.32710500
O	2.53261200	-2.35546700	0.38439200
C	0.86326300	-0.50575500	1.69750400
O	0.02701500	0.50628300	2.23998300
O	-3.71912000	1.70228200	-0.10290000
O	0.52988800	0.46419800	-2.01347400
H	-5.29206700	-0.34029100	0.01064700
H	-4.55277400	-2.71120300	0.01387300
H	-2.13438800	-3.29151000	-0.07165600
H	1.99734900	-1.49948100	-1.41935200
H	3.78919100	1.85482300	-0.31191700
H	1.53253600	2.43476100	-1.19990900
H	0.65150800	3.08063100	0.68810300
H	5.37735300	0.11999600	-0.34073200
H	4.82776000	-1.32860600	-1.20629700
H	4.84368400	-1.32425200	0.54809300
H	1.90356500	-3.05884700	0.18689900
H	1.90114400	-0.35382300	2.00301000
H	0.53309500	-1.45814800	2.11130300
H	0.48457600	1.34768700	2.07241400
H	-2.92425700	2.27993400	-0.14022200
H	0.03554700	-0.33994300	-2.21873600

Table S5. Energy of all conformers of diaporthone D (**4**).

compound	conformer	E_{tot} (a.u.)	$G_{298.15}$ (a.u.)	P (%)
**4**	**4-a**	-1033.827651	-1033.556385	18.26%
	**4-b**	-1033.825971	-1033.556269	16.15%
	**4-c**	-1033.827221	-1033.556004	12.19%
	**4-d**	-1033.827219	-1033.555981	11.91%
	**4-e**	-1033.826509	-1033.555857	10.44%
	**4-f**	-1033.825952	-1033.555652	8.40%
	**4-g**	-1033.826526	-1033.555202	5.22%
	**4-h**	-1033.825788	-1033.555028	4.34%
	**4-i**	-1033.82362	-1033.554905	3.81%
	**4-j**	-1033.824412	-1033.554629	2.84%
	**4-k**	-1033.824707	-1033.554528	2.55%
	**4-l**	-1033.824384	-1033.553745	1.12%

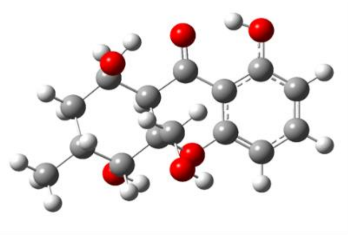
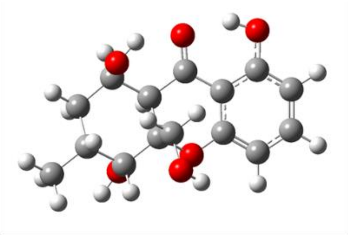
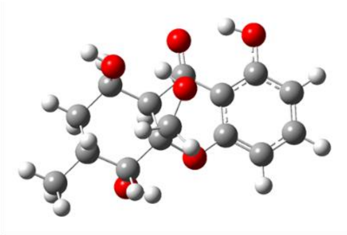

4-a **4-b** **4-c**

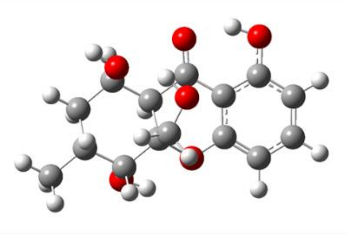
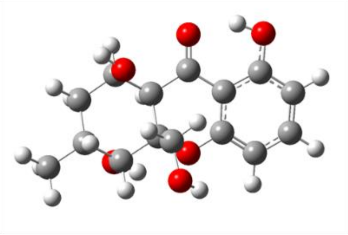
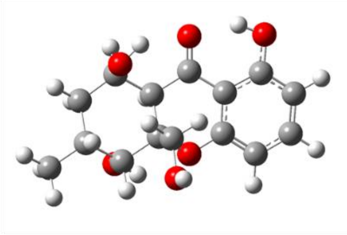

4-d **4-e** **4-f**

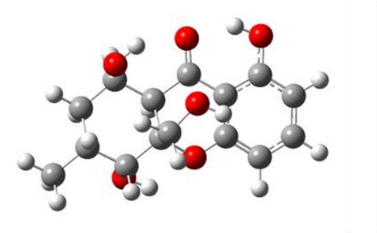
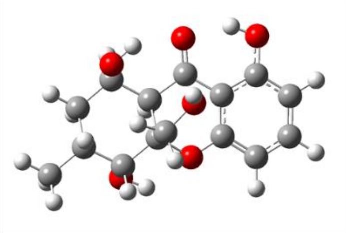
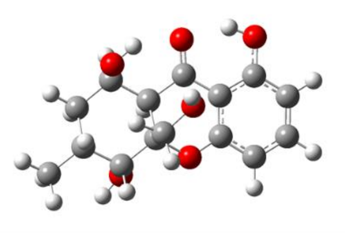

4-g **4-h** **4-i**

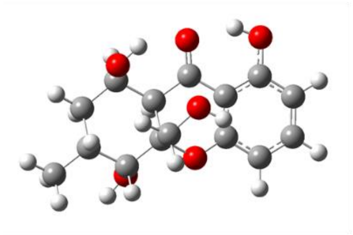
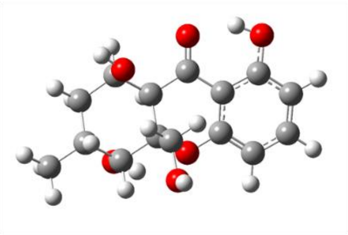
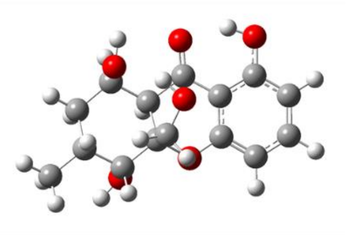

4-j **4-k** **4-l**

Figure S59. B3LYP/6-311g** optimized low-energy conformers of diaporthone D (**4**).

Table S6. Cartesian coordinates of the low-energy reoptimized conformers of **4** calculated at b3lyp/6-311g** level of theory in PCM for methanol.

**4-a**			
Atom	X axis (Å)	Y axis (Å)	Z axis (Å)
C	1.565362	-1.088861	-0.24815
C	1.925603	0.271627	-0.177181
C	3.295477	0.613521	-0.138031
C	4.26451	-0.379393	-0.214468
C	3.870553	-1.702678	-0.310813
C	2.531858	-2.074459	-0.323134
O	0.270392	-1.48854	-0.240546
C	-0.732654	-0.542549	0.206898

C	-0.492206	0.792112	-0.500567
C	0.893955	1.294528	-0.206187
C	-2.073818	-1.128962	-0.259498
C	-3.213554	-0.143902	0.009964
C	-2.949505	1.206568	-0.662222
C	-1.605334	1.824354	-0.304013
O	1.133243	2.490721	-0.046248
O	-1.661113	2.317054	1.02886
C	-4.558082	-0.721057	-0.420411
O	-2.031674	-1.408622	-1.648689
C	-0.634777	-0.471623	1.737689
O	-0.821059	-1.732716	2.343931
O	3.685733	1.889992	-0.043566
H	-0.481179	0.551146	-1.571702
H	5.309709	-0.10289	-0.189242
H	4.628752	-2.474025	-0.364693
H	2.233809	-3.112589	-0.3718
H	-2.253675	-2.052269	0.29943
H	-3.249968	0.008327	1.091301
H	-2.985464	1.076426	-1.746632
H	-3.737822	1.91301	-0.396064
H	-1.406515	2.65664	-0.985355
H	-0.85963	2.827105	1.178804
H	-4.588725	-0.876524	-1.499851
H	-4.746529	-1.679607	0.06765
H	-5.366361	-0.038812	-0.150893
H	-1.287942	-1.997726	-1.802415
H	-1.399036	0.195688	2.124907
H	0.334783	-0.046386	2.020153
H	-0.153737	-2.332237	1.999124
H	2.881044	2.446242	0.008107

**4-b**			
Atom	X axis (Å)	Y axis (Å)	Z axis (Å)
C	1.571137	-1.097387	-0.245201
C	1.934334	0.263159	-0.176852
C	3.305137	0.604374	-0.132479
C	4.273814	-0.388333	-0.20072
C	3.87847	-1.712318	-0.294749
C	2.540743	-2.083209	-0.31254
O	0.282051	-1.499524	-0.245617
C	-0.728668	-0.556731	0.17728
C	-0.482874	0.787798	-0.512954
C	0.905094	1.285403	-0.213301

C	-2.073181	-1.120847	-0.290958
C	-3.205512	-0.135052	0.04215
C	-2.947609	1.223251	-0.614777
C	-1.588285	1.826034	-0.290092
O	1.14495	2.482803	-0.056926
O	-1.608297	2.319894	1.043629
C	-4.564659	-0.692561	-0.368913
O	-1.97045	-1.350109	-1.689133
C	-0.647282	-0.49065	1.711711
O	-0.858422	-1.751606	2.310129
O	3.69483	1.881566	-0.040451
H	-0.474593	0.564597	-1.587591
H	5.319129	-0.112729	-0.171519
H	4.636464	-2.484341	-0.343168
H	2.242459	-3.121285	-0.361379
H	-2.245067	-2.066887	0.229698
H	-3.219623	-0.008961	1.127149
H	-3.014155	1.104746	-1.699209
H	-3.721301	1.934467	-0.320188
H	-1.399772	2.657245	-0.975582
H	-0.798047	2.821862	1.173326
H	-4.743592	-1.669677	0.085179
H	-5.362726	-0.020681	-0.04915
H	-4.642913	-0.79652	-1.454212
H	-2.691294	-1.922828	-1.955547
H	-1.400097	0.187878	2.099775
H	0.328527	-0.084373	1.999945
H	-0.203959	-2.361612	1.958727
H	2.889293	2.437323	0.005071

4-c

Atom	X axis (Å)	Y axis (Å)	Z axis (Å)
C	1.542955	-1.192143	0.017479
C	1.892167	0.143979	-0.246841
C	3.257553	0.485155	-0.338601
C	4.234776	-0.493767	-0.197464
C	3.851117	-1.80176	0.040727
C	2.515403	-2.166528	0.153778
O	0.25279	-1.587705	0.127684
C	-0.759364	-0.589539	0.397099
C	-0.547705	0.578845	-0.569967
C	0.850217	1.136381	-0.474869
C	-2.089228	-1.291961	0.059044
C	-3.265136	-0.307298	0.04751

C	-2.99187	0.885669	-0.872182
C	-1.679809	1.595634	-0.552025
O	1.097953	2.319919	-0.684154
O	-1.711624	2.222343	0.73115
C	-4.568161	-1.010102	-0.317829
O	-2.008277	-1.909743	-1.211603
C	-0.714069	-0.236322	1.898987
O	0.110059	0.85851	2.228803
O	3.639906	1.747543	-0.573173
H	-0.578297	0.123589	-1.568464
H	5.277325	-0.216638	-0.275515
H	4.613956	-2.562661	0.151253
H	2.223754	-3.187482	0.357543
H	-2.269908	-2.053474	0.829664
H	-3.371512	0.076037	1.064455
H	-2.955546	0.551571	-1.912686
H	-3.815413	1.600713	-0.798667
H	-1.471444	2.351958	-1.312499
H	-2.370598	2.919103	0.727137
H	-4.52691	-1.406351	-1.333119
H	-4.770004	-1.838654	0.364325
H	-5.403599	-0.310251	-0.257045
H	-1.201396	-2.433511	-1.221938
H	-0.321896	-1.110985	2.418753
H	-1.730281	-0.063868	2.258736
H	-0.374353	1.646409	1.94343
H	2.826594	2.29009	-0.654087

4-d

Atom	X axis (Å)	Y axis (Å)	Z axis (Å)
C	1.543021	-1.192071	0.017817
C	1.892238	0.144058	-0.246677
C	3.257639	0.485085	-0.338776
C	4.234836	-0.493937	-0.197807
C	3.851185	-1.801875	0.040591
C	2.515445	-2.16651	0.153998
O	0.252891	-1.58757	0.128572
C	-0.759467	-0.589374	0.397485
C	-0.547638	0.578795	-0.569833
C	0.850204	1.136442	-0.474571
C	-2.089212	-1.292032	0.059335
C	-3.265241	-0.307458	0.047136
C	-2.991749	0.885338	-0.872716
C	-1.67984	1.595513	-0.552282

O	1.097796	2.320031	-0.683664
O	-1.712132	2.222461	0.730753
C	-4.568075	-1.010508	-0.318491
O	-2.007742	-1.910328	-1.211026
C	-0.714402	-0.235702	1.899208
O	0.109593	0.859288	2.228608
O	3.64017	1.747335	-0.573644
H	-0.578069	0.123348	-1.568202
H	5.277356	-0.216846	-0.276202
H	4.613986	-2.562821	0.150969
H	2.22376	-3.187405	0.357922
H	-2.270051	-2.053264	0.830147
H	-3.371956	0.076062	1.063935
H	-2.954998	0.550996	-1.913096
H	-3.815368	1.600295	-0.799659
H	-1.471397	2.35173	-1.312791
H	-2.370163	2.920058	0.726046
H	-4.52593	-1.407867	-1.333277
H	-4.770627	-1.838247	0.3644
H	-5.403484	-0.310532	-0.259214
H	-1.200601	-2.433669	-1.221049
H	-0.322316	-1.110121	2.419385
H	-1.730659	-0.063161	2.258717
H	-0.374902	1.646936	1.942692
H	2.827148	2.290194	-0.654022

**4-e**			
Atom	X axis (Å)	Y axis (Å)	Z axis (Å)
C	1.568224	-1.065399	-0.255148
C	1.927251	0.293559	-0.172708
C	3.298873	0.630224	-0.130516
C	4.266313	-0.366584	-0.184655
C	3.871591	-1.689011	-0.281661
C	2.531766	-2.054952	-0.31574
O	0.271925	-1.461557	-0.290025
C	-0.731669	-0.54006	0.200888
C	-0.501783	0.827437	-0.448202
C	0.900365	1.32716	-0.215433
C	-2.069794	-1.112808	-0.297883
C	-3.219455	-0.143837	-0.011591
C	-2.949916	1.233933	-0.622867
C	-1.615379	1.832315	-0.182019
O	1.168236	2.523121	-0.153385
O	-1.606672	2.167139	1.201466

C	-4.553895	-0.712491	-0.48222
O	-2.012047	-1.347298	-1.69514
C	-0.632096	-0.552166	1.733372
O	-0.771035	-1.85412	2.263035
O	3.694117	1.905732	-0.049349
H	-0.53352	0.63006	-1.528161
H	5.311649	-0.091345	-0.150314
H	4.628162	-2.462703	-0.323292
H	2.229366	-3.091311	-0.375817
H	-2.251352	-2.054754	0.228777
H	-3.271046	-0.029074	1.07392
H	-2.952917	1.157884	-1.713539
H	-3.755976	1.92027	-0.350997
H	-1.403785	2.728674	-0.771818
H	-2.251644	2.862729	1.345229
H	-4.566112	-0.834355	-1.566242
H	-4.745572	-1.686573	-0.027196
H	-5.369821	-0.042626	-0.204514
H	-1.256116	-1.917535	-1.860684
H	-1.41888	0.060954	2.160652
H	0.322989	-0.109264	2.03703
H	-0.101158	-2.413541	1.86059
H	2.887179	2.464446	-0.048143

4-f

Atom	X axis (Å)	Y axis (Å)	Z axis (Å)
C	1.568316	-1.100036	-0.255115
C	1.933421	0.259785	-0.175473
C	3.303678	0.597867	-0.119566
C	4.27066	-0.397111	-0.187398
C	3.87348	-1.719341	-0.293379
C	2.53458	-2.087583	-0.32301
O	0.275352	-1.491647	-0.271164
C	-0.721251	-0.553563	0.194615
C	-0.481904	0.784624	-0.512095
C	0.904617	1.284729	-0.213464
C	-2.067619	-1.133028	-0.268576
C	-3.201475	-0.145383	0.013417
C	-2.942145	1.201594	-0.667958
C	-1.593432	1.822361	-0.330679
O	1.145251	2.481441	-0.054901
O	-1.642015	2.350892	0.988814
C	-4.552886	-0.720088	-0.398323
O	-2.032704	-1.400642	-1.660796

C	-0.630634	-0.460172	1.71807
O	-0.885345	-1.737405	2.272062
O	3.696059	1.873786	-0.017589
H	-0.466159	0.538525	-1.581853
H	5.316392	-0.124126	-0.148597
H	4.630394	-2.492479	-0.340912
H	2.234502	-3.124734	-0.378423
H	-2.245877	-2.059939	0.283004
H	-3.223265	0.009141	1.095006
H	-2.991432	1.064427	-1.750925
H	-3.726258	1.911139	-0.397483
H	-1.397008	2.637692	-1.03315
H	-0.832086	2.85217	1.123568
H	-5.356168	-0.034914	-0.121164
H	-4.597674	-0.879065	-1.476827
H	-4.737589	-1.676596	0.095211
H	-1.291951	-1.991657	-1.82151
H	-1.346076	0.284915	2.067149
H	0.368588	-0.110398	2.000183
H	-0.867374	-1.655063	3.226857
H	2.89181	2.431633	0.022079

**4-g**			
Atom	X axis (Å)	Y axis (Å)	Z axis (Å)
C	1.52087	-1.18074	0.0013
C	1.872315	0.160858	-0.238119
C	3.234054	0.498293	-0.376945
C	4.207358	-0.492572	-0.327255
C	3.822532	-1.805753	-0.118499
C	2.491779	-2.165638	0.053269
O	0.238509	-1.568832	0.201187
C	-0.788269	-0.568831	0.420331
C	-0.556544	0.592312	-0.546216
C	0.82528	1.160589	-0.387968
C	-2.109638	-1.272458	0.052292
C	-3.283477	-0.286607	0.042171
C	-3.003007	0.909041	-0.873343
C	-1.690023	1.618092	-0.575342
O	1.054671	2.364457	-0.476123
O	-1.819384	2.334969	0.644558
C	-4.585152	-0.984259	-0.338236
O	-2.006752	-1.866848	-1.227712
C	-0.791809	-0.207219	1.909717
O	0.303792	0.58747	2.319595

O	3.612992	1.768426	-0.572564
H	-0.541929	0.132615	-1.543568
H	5.247671	-0.221517	-0.445211
H	4.582766	-2.575802	-0.075157
H	2.204163	-3.190532	0.242226
H	-2.300131	-2.048273	0.804908
H	-3.396711	0.085206	1.062693
H	-2.975814	0.567616	-1.911265
H	-3.817555	1.631471	-0.793732
H	-1.47612	2.323003	-1.384442
H	-0.991983	2.80047	0.797415
H	-5.41948	-0.283011	-0.279064
H	-4.536441	-1.372596	-1.356345
H	-4.794508	-1.817683	0.33572
H	-1.224826	-2.426635	-1.222223
H	-0.832096	-1.142676	2.475804
H	-1.680869	0.374975	2.132751
H	1.078364	0.030328	2.423187
H	2.80727	2.325257	-0.558155

	**4-h**		
Atom	X axis (Å)	Y axis (Å)	Z axis (Å)
C	1.524335	-1.181584	0.023486
C	1.876878	0.153555	-0.241273
C	3.238151	0.487583	-0.383911
C	4.21133	-0.502344	-0.319211
C	3.825239	-1.812004	-0.088869
C	2.49439	-2.166943	0.089655
O	0.242055	-1.567828	0.222816
C	-0.78505	-0.568174	0.430548
C	-0.551613	0.585957	-0.544848
C	0.832514	1.152783	-0.39631
C	-2.105346	-1.275253	0.059185
C	-3.28137	-0.291726	0.030109
C	-2.994893	0.900193	-0.888341
C	-1.684739	1.611217	-0.582513
O	1.062319	2.355977	-0.500697
O	-1.82348	2.330615	0.635209
C	-4.576981	-0.995355	-0.360243
O	-1.991248	-1.88006	-1.214649
C	-0.801318	-0.198165	1.910171
O	0.335531	0.581037	2.233197
O	3.617726	1.755913	-0.598087
H	-0.540502	0.116954	-1.537755

H	5.251879	-0.234035	-0.441776
H	4.584688	-2.582238	-0.034151
H	2.205876	-3.188506	0.295052
H	-2.300755	-2.044761	0.816918
H	-3.406506	0.085909	1.046905
H	-2.958725	0.554779	-1.924697
H	-3.811154	1.621724	-0.818356
H	-1.465518	2.315032	-1.391164
H	-0.991183	2.782799	0.800405
H	-4.514941	-1.391841	-1.374437
H	-4.792659	-1.823763	0.317901
H	-5.413438	-0.295473	-0.316705
H	-1.206129	-2.435189	-1.199436
H	-0.806023	-1.13346	2.48024
H	-1.712996	0.356378	2.127961
H	0.194497	0.980071	3.093119
H	2.811193	2.311932	-0.588144

4-i

Atom	X axis (Å)	Y axis (Å)	Z axis (Å)
C	1.529425	-1.185178	0.023467
C	1.882612	0.149658	-0.246397
C	3.243893	0.486726	-0.388044
C	4.219356	-0.499482	-0.319727
C	3.834755	-1.80982	-0.084764
C	2.505766	-2.167344	0.093217
O	0.255743	-1.576811	0.22714
C	-0.783252	-0.591623	0.405912
C	-0.546645	0.580914	-0.550135
C	0.837934	1.145731	-0.39524
C	-2.10292	-1.278986	0.01713
C	-3.278482	-0.284389	0.05998
C	-3.001998	0.932489	-0.827625
C	-1.671397	1.618306	-0.555517
O	1.063897	2.351708	-0.488493
O	-1.763623	2.341975	0.664439
C	-4.589471	-0.959495	-0.332839
O	-1.921829	-1.819518	-1.281362
C	-0.820408	-0.226775	1.890224
O	0.348635	0.4911	2.239618
O	3.619662	1.756561	-0.603749
H	-0.540941	0.133493	-1.552548
H	5.259529	-0.229317	-0.441325
H	4.595986	-2.578176	-0.026308

H	2.219845	-3.189065	0.301521
H	-2.287471	-2.088767	0.732993
H	-3.387005	0.049953	1.093521
H	-3.005287	0.613032	-1.87313
H	-3.804047	1.66437	-0.714108
H	-1.465433	2.319567	-1.369861
H	-0.924542	2.79427	0.792506
H	-5.425732	-0.27478	-0.184052
H	-4.59183	-1.246563	-1.387605
H	-4.77314	-1.852097	0.269442
H	-2.662076	-2.397458	-1.474583
H	-0.882357	-1.163593	2.454782
H	-1.703562	0.374506	2.095108
H	0.191387	0.93571	3.073808
H	2.811777	2.310406	-0.588753

	**4-j**		
Atom	X axis (Å)	Y axis (Å)	Z axis (Å)
C	1.52727	-1.181616	-0.000581
C	1.878587	0.16047	-0.241807
C	3.239861	0.501407	-0.381782
C	4.215731	-0.48584	-0.333417
C	3.833136	-1.800275	-0.122306
C	2.504525	-2.163409	0.050882
O	0.25384	-1.575601	0.208249
C	-0.787186	-0.591429	0.396116
C	-0.55078	0.590542	-0.547607
C	0.830989	1.157026	-0.38097
C	-2.105414	-1.275387	0.002663
C	-3.279197	-0.280502	0.066694
C	-3.008715	0.942538	-0.814248
C	-1.676929	1.627292	-0.54497
O	1.055942	2.363881	-0.453208
O	-1.764619	2.344832	0.678548
C	-4.595012	-0.950588	-0.318304
O	-1.930548	-1.797537	-1.304291
C	-0.819559	-0.241515	1.892177
O	0.314911	0.473619	2.338942
O	3.614282	1.773735	-0.575989
H	-0.539797	0.15426	-1.555015
H	5.255392	-0.212518	-0.451694
H	4.595315	-2.568502	-0.078561
H	2.220274	-3.188993	0.241187
H	-2.286368	-2.095376	0.707745

H	-3.376142	0.045017	1.104359
H	-3.018609	0.630154	-1.861766
H	-3.809775	1.673758	-0.69057
H	-1.47475	2.332057	-1.357157
H	-0.932023	2.811032	0.798808
H	-5.428713	-0.265703	-0.156701
H	-4.60791	-1.230301	-1.374986
H	-4.774099	-1.84718	0.279282
H	-2.672153	-2.371852	-1.503098
H	-0.936203	-1.17799	2.446292
H	-1.670269	0.399506	2.098721
H	1.054283	-0.131288	2.431746
H	2.807253	2.328208	-0.55184

**4-k**			
Atom	X axis (Å)	Y axis (Å)	Z axis (Å)
C	1.569436	-1.074252	-0.265224
C	1.933863	0.283691	-0.172211
C	3.30584	0.616166	-0.114622
C	4.270618	-0.382932	-0.163268
C	3.872091	-1.704067	-0.271644
C	2.532193	-2.065971	-0.321585
O	0.275709	-1.460617	-0.323559
C	-0.720454	-0.549729	0.189123
C	-0.493468	0.822783	-0.456673
C	0.910019	1.319813	-0.22184
C	-2.064412	-1.116535	-0.304458
C	-3.20934	-0.144941	-0.009827
C	-2.942997	1.229172	-0.630933
C	-1.606234	1.832716	-0.202943
O	1.18049	2.515668	-0.161828
O	-1.596986	2.197022	1.173346
C	-4.54941	-0.712799	-0.465435
O	-2.013652	-1.343853	-1.703562
C	-0.624857	-0.541707	1.714996
O	-0.805252	-1.863816	2.189784
O	3.703705	1.891081	-0.024445
H	-0.522009	0.622207	-1.536057
H	5.316528	-0.111591	-0.11664
H	4.62705	-2.479661	-0.309498
H	2.227329	-3.101025	-0.3893
H	-2.244347	-2.060048	0.218345
H	-3.249923	-0.026222	1.075875
H	-2.952537	1.144704	-1.720905

H	-3.747625	1.91759	-0.359795
H	-1.394847	2.717699	-0.809955
H	-2.241615	2.896	1.30138
H	-4.57262	-0.838413	-1.5489
H	-4.73812	-1.685092	-0.00529
H	-5.361715	-0.040752	-0.182245
H	-1.255585	-1.909643	-1.874758
H	-1.376631	0.138688	2.112573
H	0.355784	-0.154915	2.013382
H	-0.816546	-1.833243	3.147674
H	2.89799	2.451397	-0.034504

**4-l**			
Atom	X axis (Å)	Y axis (Å)	Z axis (Å)
C	1.546648	-1.210948	-0.000519
C	1.896076	0.128858	-0.252894
C	3.260443	0.4795	-0.3261
C	4.241614	-0.494058	-0.184538
C	3.860935	-1.806498	0.039802
C	2.527299	-2.179921	0.137224
O	0.263763	-1.616442	0.097643
C	-0.755843	-0.629667	0.358448
C	-0.541699	0.554502	-0.591935
C	0.851428	1.114393	-0.473738
C	-2.098348	-1.299688	0.028923
C	-3.257138	-0.285102	0.105552
C	-2.997826	0.918408	-0.802215
C	-1.658405	1.59576	-0.551221
O	1.084955	2.308767	-0.6477
O	-1.68723	2.293102	0.702931
C	-4.593052	-0.944694	-0.223953
O	-1.983735	-1.86354	-1.266633
C	-0.711996	-0.289976	1.868459
O	0.108191	0.803585	2.209033
O	3.635426	1.747864	-0.543477
H	-0.571652	0.115822	-1.597791
H	5.283576	-0.211203	-0.248399
H	4.626818	-2.564176	0.152417
H	2.240629	-3.204128	0.331574
H	-2.267541	-2.094167	0.767058
H	-3.32037	0.068857	1.136187
H	-3.01752	0.589159	-1.84487
H	-3.79422	1.654689	-0.680988
H	-1.470892	2.320259	-1.34427

H	-1.311233	3.164679	0.565463
H	-5.412578	-0.248089	-0.041754
H	-4.648348	-1.237507	-1.27588
H	-4.760599	-1.831092	0.391833
H	-2.76515	-2.393359	-1.435431
H	-0.314396	-1.169634	2.374955
H	-1.724649	-0.126704	2.240768
H	-0.383337	1.587553	1.923873
H	2.819697	2.287148	-0.616894

Table S7. Energy of all conformers of diaporthone E (**5**).

compound	conformer	E (kcal/mol)	rel.E(kcal/mol)	Boltzmann dist (%)
**5**	**5-a**	-743961.4245	0	86.46%
	**5-b**	-743960.3269	1.097652113	13.54%

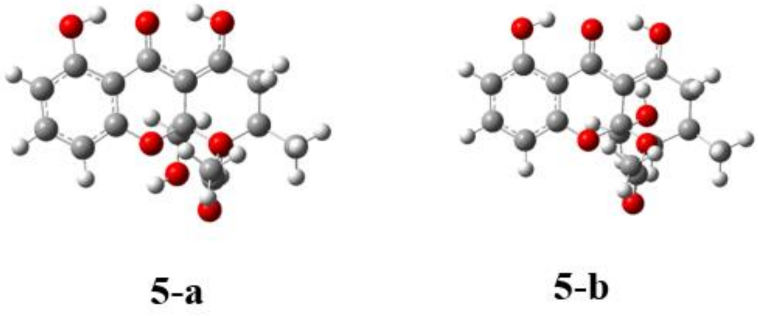

Figure S60. B3LYP/6-311g** optimized low-energy conformers of diaporthone E (**5**).

Table S8. Cartesian coordinates of the low-energy reoptimized conformers of **5** calculated at b3lyp/6-311g** level of theory in PCM for methanol.

**5-a**			
Atom	X axis (Å)	Y axis (Å)	Z axis (Å)
C	1.59118100	-1.08256200	0.20148800
C	2.23139300	0.14659600	-0.07740000
C	3.63325600	0.15078900	-0.31071000
C	4.35836900	-1.03945100	-0.22990100
C	3.69237900	-2.22802100	0.04599500
C	2.31391800	-2.26794800	0.25775800
O	0.24350900	-1.16477600	0.38079300
C	-0.47872800	0.03266500	0.79045200

C	0.02688100	1.26003800	0.06838000
C	1.43423500	1.35506000	-0.22352600
C	-1.95704200	-0.24883200	0.46796900
C	-2.81997600	1.00351600	0.66316800
C	-2.31805000	2.15976200	-0.21778300
C	-0.82821600	2.26607700	-0.31024200
O	1.95397400	2.42778300	-0.65548300
O	-0.40127300	3.39648400	-0.85630400
C	-4.30578900	0.72014700	0.42248600
O	-2.05127000	-0.65327600	-0.91716000
C	-0.25808600	0.15890800	2.31707000
O	-0.71243500	-0.98106300	3.02950600
O	4.28547800	1.28591600	-0.61597500
C	-2.33750800	-1.94802400	-1.19841800
C	-2.30924900	-2.18167100	-2.68461700
O	-2.58264200	-2.78353400	-0.36234700
H	5.42703200	-1.01478100	-0.40035500
H	4.25913400	-3.15054300	0.09943500
H	1.79802400	-3.19503200	0.47026600
H	-2.30634700	-1.06637600	1.09428300
H	-2.70523000	1.29825300	1.71025200
H	-2.68317400	2.03651900	-1.24496600
H	-2.71592500	3.11536400	0.13444900
H	0.59217800	3.33932000	-0.89703500
H	-4.48329900	0.40130000	-0.60759200
H	-4.66655100	-0.06415600	1.09243000
H	-4.90015600	1.61896600	0.60434300
H	-0.80791600	1.01659000	2.70445900
H	0.80801800	0.34366600	2.49677800
H	-0.23438100	-1.74531300	2.68591600
H	3.60515400	1.99331400	-0.70986000
H	-1.29660100	-2.01432800	-3.05869300
H	-2.96848600	-1.47346200	-3.18986300
H	-2.61800200	-3.20187400	-2.90150500

5-b

Atom	X axis (Å)	Y axis (Å)	Z axis (Å)
C	-1.53493100	1.06682300	0.36327100
C	-2.13359100	-0.10320500	-0.15437800

C	-3.50903200	-0.07085600	-0.50580400
C	-4.25254100	1.09622800	-0.31768300
C	-3.62746900	2.22857400	0.19135200
C	-2.27404500	2.23312800	0.53020900
O	-0.21491500	1.12504900	0.68287800
C	0.54621700	-0.09710800	0.88361900
C	0.07638000	-1.20968500	-0.02855800
C	-1.31497600	-1.27936900	-0.39474000
C	2.01197400	0.28029700	0.58876000
C	2.92513000	-0.95144600	0.61607300
C	2.45068200	-2.00798700	-0.39602700
C	0.96416000	-2.12900800	-0.52984400
O	-1.79605400	-2.29297900	-0.98694000
O	0.57991000	-3.17495900	-1.25176900
C	4.39596100	-0.58553100	0.39368800
O	2.07035300	0.86112800	-0.73360700
C	0.39521900	-0.46230700	2.38341000
O	-0.94199900	-0.76533200	2.74485000
O	-4.12069900	-1.14953600	-1.02634100
C	2.30642500	2.19139300	-0.85263100
C	2.24716100	2.60744200	-2.29729100
O	2.53536300	2.92330100	0.07938800
H	-5.30171800	1.09824100	-0.58403900
H	-4.20660000	3.13444700	0.33003200
H	-1.78927600	3.11599200	0.92550500
H	2.34543700	1.03089800	1.30487100
H	2.84078100	-1.37751500	1.61975300
H	2.82784100	-1.76448700	-1.39709500
H	2.86182500	-2.99050600	-0.14825200
H	-0.41354800	-3.13317900	-1.30966600
H	5.02302300	-1.47850600	0.45372000
H	4.54464900	-0.13504000	-0.59062400
H	4.74212000	0.12079000	1.15221600
H	0.79260700	0.36279100	2.98538400
H	0.97619700	-1.36016900	2.59662700
H	-1.42315700	0.06080800	2.85912700
H	-3.42854200	-1.84232500	-1.14475400
H	2.92744300	1.99574300	-2.89274700

H	2.51173300	3.65863000	-2.38758200
H	1.23717000	2.44569800	-2.68069400

Table S9. Energy of all conformers of compound **7.**

compound	conformer	E_{tot} (a.u.)	$G_{298.15}$ (a.u.)	P (%)
	**7-a**	-1109.054472	-1108.776343	26.32%
	**7-b**	-1109.052785	-1108.775977	17.86%
	**7-c**	-1109.053205	-1108.775397	9.66%
	**7-d**	-1109.051862	-1108.77532	8.90%
	**7-e**	-1109.052834	-1108.774784	5.05%
	**7-f**	-1109.053063	-1108.774674	4.49%
	**7-g**	-1109.052609	-1108.774589	4.10%
**7**	**7-h**	-1109.050718	-1108.774418	3.42%
	**7-i**	-1109.050883	-1108.774339	3.15%
	**7-j**	-1109.052817	-1108.774138	2.55%
	**7-k**	-1109.052338	-1108.77403	2.27%
	**7-l**	-1109.051143	-1108.773722	1.64%
	**7-m**	-1109.050781	-1108.773559	1.38%
	**7-n**	-1109.049731	-1108.773361	1.12%
	**7-o**	-1109.051055	-1108.773331	1.08%

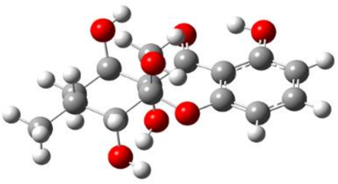
7-a

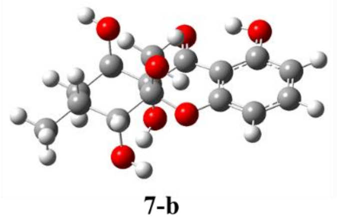
7-b

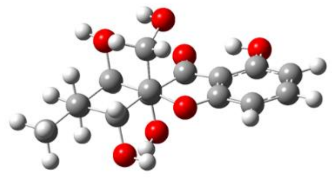
7-c

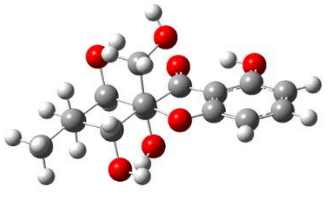
7-d

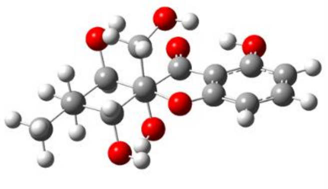
7-e

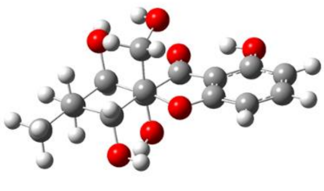
7-f

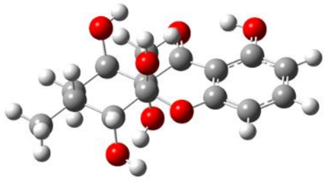
7-g

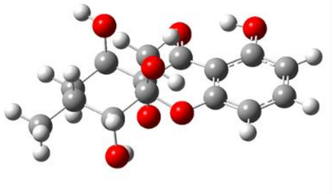
7-h

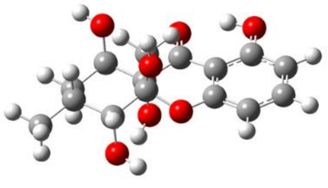
7-i

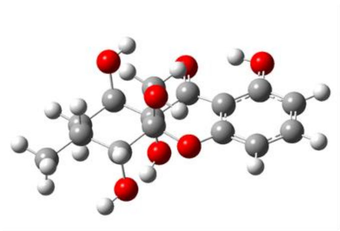

7-j

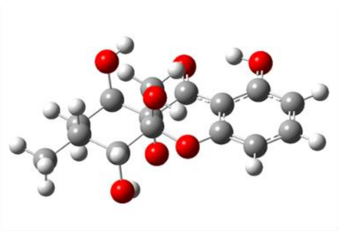

7-k

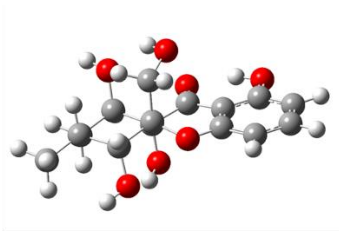

7-l

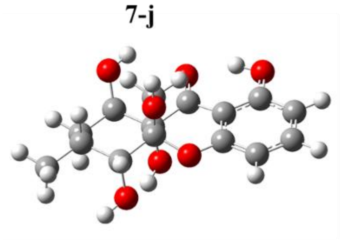

7-m

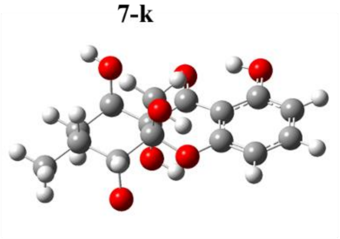

7-n

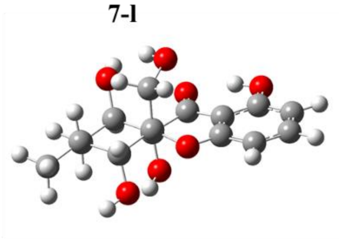

7-o

Figure S61. B3LYP/6-311g** optimized low-energy conformers of compound **7.**

Table S10. Cartesian coordinates of the low-energy reoptimized conformers of **7** calculated at b3lyp/6-311g** level of theory in PCM for methanol.

**7-a**			
Atom	X axis (Å)	Y axis (Å)	Z axis (Å)
C	4.285627	-0.408753	-0.154737
C	3.886582	-1.726737	-0.292651
C	2.54666	-2.093808	-0.312254
C	1.583408	-1.108615	-0.205751
C	1.949179	0.248826	-0.095039
C	3.320424	0.584163	-0.044431
O	0.288708	-1.507505	-0.196703
C	-0.702484	-0.565916	0.262419
C	-0.469973	0.781928	-0.45469
C	0.926322	1.275798	-0.081445
C	-2.064429	-1.154968	-0.141186
C	-3.190774	-0.16301	0.159252
C	-2.939849	1.193429	-0.50724
C	-1.58575	1.805334	-0.173521
O	3.717231	1.855074	0.092121
O	1.15262	2.461011	0.1412
O	-1.595012	2.248919	1.173467
O	-0.374273	0.603685	-1.859644
C	-0.572728	-0.488133	1.796368
O	-0.750107	-1.751764	2.397862
O	-2.07768	-1.465213	-1.536731
C	-4.54967	-0.731554	-0.234082
H	5.331833	-0.137111	-0.121749
H	4.641993	-2.498566	-0.372784

H	2.245344	-3.1291	-0.391404
H	-2.226829	-2.070207	0.431233
H	-3.194189	-0.018488	1.241999
H	-3.014797	1.083248	-1.59149
H	-3.716542	1.899211	-0.208848
H	-1.398421	2.650157	-0.841992
H	2.920373	2.415473	0.176167
H	-0.80563	2.7827	1.305052
H	-0.984172	-0.112622	-2.103237
H	0.40371	-0.069071	2.060233
H	-1.326051	0.178208	2.20287
H	-0.072537	-2.344584	2.061501
H	-1.543969	-2.252596	-1.672302
H	-5.344767	-0.047748	0.067719
H	-4.726777	-1.69341	0.251491
H	-4.61573	-0.877061	-1.313111

7-b

Atom	X axis (Å)	Y axis (Å)	Z axis (Å)
C	4.288384	-0.400405	-0.134907
C	3.88849	-1.716442	-0.282858
C	2.547289	-2.076278	-0.324689
C	1.587367	-1.087099	-0.222612
C	1.952019	0.267252	-0.090944
C	3.32498	0.596632	-0.03767
O	0.290624	-1.481362	-0.255592
C	-0.699013	-0.56548	0.252618
C	-0.479135	0.816133	-0.404935
C	0.934377	1.307178	-0.084951
C	-2.060241	-1.145471	-0.175759
C	-3.19741	-0.170948	0.142078
C	-2.944211	1.213213	-0.463022
C	-1.596122	1.805783	-0.053624
O	3.727314	1.866033	0.094557
O	1.191881	2.495959	0.045738
O	-1.537676	2.092488	1.335421
O	-0.424985	0.69998	-1.820608
C	-0.561031	-0.572914	1.788503
O	-0.677553	-1.879717	2.308891
O	-2.063719	-1.419615	-1.579141
C	-4.546732	-0.733931	-0.289989
H	5.334798	-0.130549	-0.092661
H	4.642081	-2.49055	-0.357805
H	2.241051	-3.108663	-0.422213

H	-2.221236	-2.077079	0.37055
H	-3.215066	-0.064015	1.229129
H	-2.994427	1.160296	-1.553158
H	-3.736013	1.897815	-0.150309
H	-1.39771	2.713292	-0.629863
H	2.927608	2.430925	0.131385
H	-2.134999	2.820506	1.517668
H	-1.009437	-0.032799	-2.079287
H	0.397056	-0.126601	2.072857
H	-1.340752	0.029024	2.240677
H	0.004714	-2.425876	1.908973
H	-1.506988	-2.187516	-1.733764
H	-4.595934	-0.846103	-1.373778
H	-5.350246	-0.064201	0.021211
H	-4.72566	-1.711366	0.162824

7-c

Atom	X axis (Å)	Y axis (Å)	Z axis (Å)
C	4.257661	-0.515787	-0.122021
C	3.867154	-1.822698	0.109496
C	2.529162	-2.180787	0.218512
C	1.562019	-1.201736	0.083362
C	1.918829	0.133946	-0.175819
C	3.285579	0.468375	-0.261295
O	0.269806	-1.591913	0.196506
C	-0.72441	-0.585272	0.463979
C	-0.525734	0.568927	-0.543198
C	0.887679	1.137896	-0.389718
C	-2.07994	-1.278652	0.212428
C	-3.242529	-0.277302	0.213098
C	-2.98916	0.907759	-0.723841
C	-1.655478	1.601889	-0.459128
O	3.677378	1.730023	-0.488074
O	1.127888	2.322902	-0.575477
O	-1.628476	2.221528	0.823395
O	-0.489041	0.083261	-1.878427
C	-0.635705	-0.192	1.958406
O	0.226647	0.880598	2.257227
O	-2.059313	-1.960141	-1.041115
C	-4.565886	-0.967341	-0.09843
H	5.301682	-0.243126	-0.195721
H	4.625907	-2.587769	0.218945
H	2.231891	-3.200797	0.418861
H	-2.241891	-2.009865	1.01215

H	-3.309967	0.118141	1.22833
H	-3.016504	0.581593	-1.765856
H	-3.793701	1.637839	-0.608513
H	-1.459371	2.34901	-1.231246
H	2.872472	2.283297	-0.558854
H	-2.216257	2.978981	0.820914
H	-1.049641	-0.70864	-1.925644
H	-1.636896	0.013011	2.339151
H	-0.256213	-1.071892	2.478689
H	-0.25084	1.681342	1.998323
H	-1.425109	-2.679928	-0.972035
H	-5.388904	-0.256911	-0.004606
H	-4.749245	-1.791991	0.593394
H	-4.571948	-1.365655	-1.113559

7-d

Atom	X axis (Å)	Y axis (Å)	Z axis (Å)
C	4.236847	-0.525578	-0.22336
C	3.843687	-1.832688	0.006638
C	2.509334	-2.181636	0.173764
C	1.544316	-1.193844	0.093497
C	1.904856	0.139626	-0.173032
C	3.268038	0.467889	-0.300793
O	0.258138	-1.575248	0.285303
C	-0.750621	-0.56676	0.491148
C	-0.527828	0.569021	-0.529243
C	0.870906	1.146577	-0.325353
C	-2.101019	-1.264348	0.215313
C	-3.26073	-0.260042	0.199918
C	-2.990997	0.923589	-0.735741
C	-1.65487	1.614688	-0.498604
O	3.657666	1.734008	-0.51378
O	1.091258	2.349338	-0.412749
O	-1.710661	2.336754	0.720113
O	-0.445129	0.060246	-1.85469
C	-0.725011	-0.147738	1.964177
O	0.418397	0.62918	2.2667
O	-2.057713	-1.943466	-1.0383
C	-4.579873	-0.946157	-0.138501
H	5.279518	-0.261212	-0.335832
H	4.599522	-2.605668	0.071194
H	2.214782	-3.201246	0.380243
H	-2.277033	-1.998111	1.00875
H	-3.348394	0.127732	1.216552

H	-3.024397	0.584261	-1.773353
H	-3.78548	1.663207	-0.623031
H	-1.457043	2.302047	-1.326098
H	2.860274	2.29974	-0.503435
H	-0.897595	2.846002	0.788628
H	-1.02716	-0.714916	-1.912945
H	-1.628853	0.410181	2.19381
H	-0.71913	-1.072524	2.550523
H	0.183476	1.262763	2.945775
H	-1.441884	-2.677229	-0.953944
H	-5.402382	-0.233379	-0.059507
H	-4.779453	-1.771883	0.547551
H	-4.566629	-1.341838	-1.154628

**7-e**			
Atom	X axis (Å)	Y axis (Å)	Z axis (Å)
C	4.232561	-0.510807	-0.238545
C	3.841033	-1.822549	-0.035663
C	2.507179	-2.17762	0.124539
C	1.540516	-1.190269	0.064062
C	1.899516	0.15117	-0.171569
C	3.26338	0.48366	-0.295541
O	0.255406	-1.575067	0.256287
C	-0.754033	-0.569175	0.479026
C	-0.534051	0.582157	-0.524171
C	0.863313	1.158951	-0.312361
C	-2.104455	-1.261799	0.195793
C	-3.261978	-0.255842	0.207418
C	-2.998378	0.938334	-0.716553
C	-1.661753	1.627198	-0.475735
O	3.651334	1.752529	-0.484267
O	1.08326	2.362484	-0.376665
O	-1.717184	2.330242	0.752924
O	-0.449401	0.094254	-1.857193
C	-0.720474	-0.17474	1.966173
O	0.392882	0.601748	2.355685
O	-2.069172	-1.915646	-1.071814
C	-4.586426	-0.934342	-0.125332
H	5.274884	-0.243231	-0.345847
H	4.597947	-2.595469	0.01347
H	2.21441	-3.20158	0.310499
H	-2.276832	-2.011267	0.975198
H	-3.336189	0.118867	1.230155
H	-3.036964	0.611082	-1.757862

H	-3.792396	1.676395	-0.591128
H	-1.466577	2.325896	-1.294478
H	2.854429	2.318711	-0.465068
H	-0.896335	2.823129	0.844197
H	-1.033448	-0.678439	-1.92835
H	-1.594728	0.423386	2.199721
H	-0.766551	-1.106229	2.538225
H	1.160739	0.0333	2.448765
H	-1.462849	-2.65882	-1.003598
H	-5.406623	-0.221551	-0.025313
H	-4.779193	-1.770338	0.550128
H	-4.586432	-1.314163	-1.147633

**7-f**			
Atom	X axis (Å)	Y axis (Å)	Z axis (Å)
C	4.255292	-0.519375	-0.123212
C	3.865672	-1.827793	0.10212
C	2.528272	-2.188347	0.211636
C	1.559405	-1.210378	0.081715
C	1.916084	0.126845	-0.17369
C	3.282123	0.464355	-0.25563
O	0.267884	-1.601705	0.199958
C	-0.727066	-0.593732	0.461371
C	-0.524157	0.55385	-0.55201
C	0.883111	1.127165	-0.382933
C	-2.085245	-1.282531	0.216853
C	-3.241002	-0.272824	0.231107
C	-2.989392	0.908546	-0.710648
C	-1.649218	1.596765	-0.480674
O	3.671024	1.728401	-0.474059
O	1.112817	2.319446	-0.542257
O	-1.653974	2.287528	0.772096
O	-0.479195	0.05878	-1.883732
C	-0.64343	-0.195117	1.954751
O	0.212054	0.88172	2.255155
O	-2.078356	-1.959412	-1.039003
C	-4.572176	-0.953111	-0.068642
H	5.299109	-0.245646	-0.195477
H	4.6253	-2.592598	0.20719
H	2.233262	-3.209489	0.409452
H	-2.244706	-2.015423	1.015491
H	-3.299471	0.12179	1.247217
H	-3.032922	0.570195	-1.748329
H	-3.782169	1.647846	-0.58693

H	-1.464711	2.31097	-1.283523
H	2.866695	2.282931	-0.533494
H	-1.337884	3.180158	0.620581
H	-1.049882	-0.725671	-1.930906
H	-1.646018	0.00788	2.333663
H	-0.264578	-1.072361	2.479576
H	-0.27418	1.673765	1.987524
H	-1.461439	-2.694137	-0.973259
H	-5.389083	-0.236907	0.033728
H	-4.754765	-1.777367	0.623815
H	-4.590225	-1.349904	-1.084277

	**7-g**		
Atom	X axis (Å)	Y axis (Å)	Z axis (Å)
C	4.291362	-0.426388	-0.1279
C	3.889241	-1.743272	-0.273226
C	2.549093	-2.106364	-0.30858
C	1.585864	-1.119156	-0.209556
C	1.956409	0.237755	-0.092892
C	3.328122	0.568879	-0.026787
O	0.293293	-1.510346	-0.222488
C	-0.692167	-0.575762	0.251587
C	-0.460141	0.776255	-0.464782
C	0.936599	1.267293	-0.087813
C	-2.058714	-1.15896	-0.149293
C	-3.179086	-0.163738	0.160846
C	-2.932134	1.189151	-0.514758
C	-1.575466	1.805835	-0.198343
O	3.727167	1.839039	0.1158
O	1.164626	2.453347	0.131978
O	-1.581498	2.287165	1.135569
O	-0.359981	0.593893	-1.86885
C	-0.571287	-0.476105	1.777099
O	-0.812097	-1.758796	2.324269
O	-2.077883	-1.460439	-1.546966
C	-4.543746	-0.729952	-0.21533
H	5.338046	-0.158338	-0.082434
H	4.643387	-2.51711	-0.346412
H	2.245782	-3.140645	-0.392465
H	-2.21987	-2.076366	0.418397
H	-3.168855	-0.016135	1.243308
H	-3.016724	1.071401	-1.597537
H	-3.706211	1.897273	-0.214995
H	-1.389622	2.63317	-0.888832

H	2.930693	2.401607	0.188073
H	-0.783297	2.811602	1.252671
H	-0.97247	-0.120433	-2.111962
H	0.432989	-0.12914	2.041254
H	-1.279791	0.262907	2.147623
H	-0.801103	-1.679453	3.279479
H	-1.551696	-2.252033	-1.687083
H	-5.334077	-0.0431	0.092179
H	-4.717931	-1.689593	0.275677
H	-4.621966	-0.879347	-1.293082

**7-h**			
Atom	X axis (Å)	Y axis (Å)	Z axis (Å)
C	4.293733	-0.400309	-0.116917
C	3.894522	-1.714501	-0.288066
C	2.554923	-2.074797	-0.351205
C	1.59077	-1.089089	-0.242982
C	1.956234	0.263333	-0.083278
C	3.329578	0.594291	-0.01601
O	0.299302	-1.48346	-0.3057
C	-0.704252	-0.591798	0.212344
C	-0.478956	0.811404	-0.384788
C	0.939759	1.297478	-0.067688
C	-2.07522	-1.156291	-0.221893
C	-3.197356	-0.173311	0.165555
C	-2.947207	1.221925	-0.409276
C	-1.590788	1.797773	-0.006088
O	3.728322	1.862247	0.135583
O	1.190421	2.490317	0.054306
O	-1.501281	2.057725	1.383228
O	-0.482449	0.643413	-1.805843
C	-0.565925	-0.635874	1.751357
O	-0.666214	-1.95643	2.236572
O	-2.110387	-1.512352	-1.589783
C	-4.561004	-0.710081	-0.252019
H	5.33969	-0.131551	-0.060565
H	4.649279	-2.487162	-0.366683
H	2.251142	-3.105495	-0.471132
H	-2.228908	-2.086156	0.326915
H	-3.193899	-0.094387	1.255446
H	-3.011896	1.192678	-1.500104
H	-3.728131	1.908395	-0.073375
H	-1.401272	2.722861	-0.562304
H	2.929158	2.427633	0.163881

H	-2.112431	2.765735	1.597091
H	-0.417681	1.505204	-2.22914
H	0.387645	-0.187391	2.047026
H	-1.35068	-0.056788	2.222879
H	0.00685	-2.489136	1.804034
H	-1.689596	-0.801479	-2.087778
H	-4.627992	-0.803732	-1.3368
H	-5.352704	-0.037779	0.084028
H	-4.743147	-1.694033	0.185398

7-i

Atom	X axis (Å)	Y axis (Å)	Z axis (Å)
C	4.292261	-0.416559	-0.112359
C	3.889102	-1.731203	-0.270334
C	2.548024	-2.087041	-0.327728
C	1.588209	-1.096083	-0.230783
C	1.957587	0.257352	-0.090298
C	3.330999	0.582508	-0.021752
O	0.294361	-1.480783	-0.286836
C	-0.688346	-0.57474	0.240159
C	-0.471554	0.812793	-0.413948
C	0.943322	1.300329	-0.091722
C	-2.054417	-1.149736	-0.182967
C	-3.186665	-0.173441	0.144589
C	-2.938452	1.207674	-0.469843
C	-1.589033	1.807275	-0.073385
O	3.735154	1.851207	0.118498
O	1.20383	2.489221	0.036087
O	-1.531647	2.126066	1.309128
O	-0.415988	0.695605	-1.82954
C	-0.554016	-0.56029	1.768103
O	-0.707822	-1.888501	2.234666
O	-2.064744	-1.4157	-1.588239
C	-4.541467	-0.73623	-0.270408
H	5.339096	-0.150476	-0.057975
H	4.641433	-2.507108	-0.340487
H	2.239853	-3.118123	-0.431348
H	-2.212585	-2.083428	0.359019
H	-3.191575	-0.063131	1.231593
H	-2.995571	1.146469	-1.559206
H	-3.729384	1.893396	-0.157248
H	-1.391744	2.703013	-0.667972
H	2.935776	2.417402	0.144233
H	-2.122198	2.864335	1.4708

H	-1.001268	-0.036764	-2.087545
H	0.42993	-0.167389	2.043891
H	-1.301632	0.106196	2.19181
H	-0.726199	-1.862495	3.192629
H	-1.512314	-2.185621	-1.747812
H	-5.341028	-0.064669	0.047061
H	-4.716303	-1.711949	0.187714
H	-4.602776	-0.852226	-1.353213

7-j

Atom	X axis (Å)	Y axis (Å)	Z axis (Å)
C	4.291911	-0.412662	-0.143085
C	3.893339	-1.730634	-0.28772
C	2.554229	-2.097761	-0.317135
C	1.588533	-1.113251	-0.213306
C	1.954822	0.243519	-0.095589
C	3.326033	0.579289	-0.036147
O	0.29696	-1.514454	-0.217299
C	-0.700298	-0.579815	0.236847
C	-0.465133	0.77805	-0.461706
C	0.93192	1.268431	-0.082745
C	-2.065952	-1.155108	-0.159937
C	-3.184157	-0.159042	0.186359
C	-2.938093	1.201748	-0.470448
C	-1.575103	1.80283	-0.157992
O	3.720992	1.850311	0.106482
O	1.157361	2.454168	0.141078
O	-1.560282	2.239769	1.191255
O	-0.368391	0.621256	-1.868822
C	-0.574208	-0.514004	1.774215
O	-0.757116	-1.780588	2.366675
O	-2.012797	-1.444804	-1.557231
C	-4.557546	-0.710143	-0.186395
H	5.337915	-0.141246	-0.10278
H	4.649365	-2.502102	-0.365865
H	2.253575	-3.132691	-0.403207
H	-2.219687	-2.084887	0.392383
H	-3.170233	-0.035181	1.271227
H	-3.030431	1.098913	-1.554233
H	-3.705401	1.910237	-0.154714
H	-1.395013	2.650246	-0.825105
H	2.92273	2.409658	0.185323
H	-0.764885	2.767398	1.312219
H	-0.961702	-0.103547	-2.12712

H	0.405317	-0.102896	2.037925
H	-1.321354	0.153891	2.188428
H	-0.089668	-2.377174	2.016743
H	-2.858959	-1.7973	-1.840711
H	-4.684068	-0.791032	-1.269744
H	-5.340456	-0.041812	0.174236
H	-4.722631	-1.694424	0.25661

**7-k**			
Atom	X axis (Å)	Y axis (Å)	Z axis (Å)
C	4.29132	-0.408484	-0.143783
C	3.892741	-1.72493	-0.303949
C	2.55429	-2.093583	-0.338216
C	1.586853	-1.11229	-0.221141
C	1.953467	0.243881	-0.084953
C	3.325351	0.581194	-0.024697
O	0.296944	-1.51332	-0.233413
C	-0.70856	-0.591259	0.228953
C	-0.4685	0.776144	-0.435593
C	0.931506	1.26508	-0.057809
C	-2.078699	-1.167806	-0.181145
C	-3.191169	-0.163213	0.175129
C	-2.941873	1.200558	-0.471688
C	-1.58189	1.799402	-0.139952
O	3.718471	1.850618	0.131056
O	1.151306	2.453027	0.16514
O	-1.569915	2.224014	1.209329
O	-0.421071	0.537513	-1.844047
C	-0.581268	-0.539055	1.767932
O	-0.752636	-1.812931	2.346428
O	-2.118434	-1.568874	-1.5366
C	-4.561876	-0.709723	-0.205534
H	5.337232	-0.137324	-0.101154
H	4.649411	-2.494755	-0.391779
H	2.255189	-3.127643	-0.438229
H	-2.236284	-2.076886	0.400037
H	-3.177699	-0.037556	1.260483
H	-3.026404	1.105538	-1.557139
H	-3.709035	1.910646	-0.158787
H	-1.400938	2.661315	-0.792626
H	2.921163	2.410088	0.213858
H	-0.776256	2.752698	1.339143
H	-0.370353	1.378684	-2.308728
H	0.393995	-0.121979	2.039382

H	-1.336074	0.116152	2.188439
H	-0.08696	-2.40282	1.982085
H	-1.673169	-0.890711	-2.058439
H	-5.346893	-0.021292	0.112741
H	-4.743018	-1.675259	0.271462
H	-4.641356	-0.844773	-1.285212

**7-l**			
Atom	X axis (Å)	Y axis (Å)	Z axis (Å)
C	4.265452	-0.51442	-0.112874
C	3.875393	-1.822876	0.114324
C	2.538093	-2.184131	0.212792
C	1.567531	-1.207267	0.072443
C	1.925435	0.129165	-0.181332
C	3.292143	0.467072	-0.257571
O	0.279222	-1.604168	0.17087
C	-0.721881	-0.60859	0.439319
C	-0.521157	0.564099	-0.547665
C	0.893521	1.130853	-0.390733
C	-2.084384	-1.282432	0.189532
C	-3.237516	-0.261751	0.240567
C	-2.986512	0.928974	-0.68744
C	-1.642384	1.604864	-0.43689
O	3.681506	1.730778	-0.479194
O	1.131702	2.317504	-0.572167
O	-1.592304	2.211875	0.851325
O	-0.490049	0.103205	-1.891681
C	-0.635188	-0.234146	1.94249
O	0.241375	0.821673	2.256795
O	-2.005087	-1.93698	-1.074914
C	-4.580416	-0.923723	-0.056324
H	5.309446	-0.239576	-0.178531
H	4.635067	-2.586488	0.228277
H	2.241717	-3.205364	0.408183
H	-2.237394	-2.035492	0.971387
H	-3.284878	0.112597	1.26435
H	-3.027256	0.607216	-1.730554
H	-3.782097	1.66643	-0.558836
H	-1.449877	2.357606	-1.204301
H	2.87476	2.281194	-0.552841
H	-2.14908	2.992309	0.855021
H	-1.016874	-0.711094	-1.947463
H	-1.632712	-0.02089	2.326449
H	-0.267406	-1.126509	2.449621

H	-0.22519	1.632676	2.009586
H	-2.852201	-2.340705	-1.276128
H	-4.666195	-1.219256	-1.105746
H	-5.393279	-0.224006	0.141213
H	-4.736247	-1.805882	0.568209

**7-m**			
Atom	X axis (Å)	Y axis (Å)	Z axis (Å)
C	4.298218	-0.431313	-0.117349
C	3.896282	-1.748075	-0.269617
C	2.557042	-2.111148	-0.313145
C	1.591524	-1.124527	-0.215138
C	1.962708	0.231615	-0.091058
C	3.33453	0.562966	-0.017845
O	0.302369	-1.51801	-0.239368
C	-0.689628	-0.58981	0.227504
C	-0.453933	0.771802	-0.471516
C	0.943182	1.259547	-0.087053
C	-2.059009	-1.159711	-0.168478
C	-3.171676	-0.16052	0.184849
C	-2.929643	1.195697	-0.483431
C	-1.565119	1.803041	-0.186966
O	3.73184	1.833163	0.130778
O	1.170814	2.44597	0.133894
O	-1.552133	2.281463	1.148075
O	-0.3498	0.609175	-1.877351
C	-0.574741	-0.498464	1.75649
O	-0.833821	-1.779152	2.299281
O	-2.010226	-1.441	-1.568084
C	-4.549951	-0.710846	-0.170488
H	5.34471	-0.163625	-0.065986
H	4.650902	-2.521568	-0.342296
H	2.254237	-3.144965	-0.404139
H	-2.21227	-2.091418	0.378879
H	-3.145137	-0.031672	1.269083
H	-3.03013	1.083431	-1.565521
H	-3.694957	1.906712	-0.168475
H	-1.385619	2.631395	-0.877853
H	2.934032	2.394444	0.199802
H	-0.750316	2.802212	1.256138
H	-0.944915	-0.114097	-2.13583
H	0.434592	-0.167755	2.020717
H	-1.271017	0.249427	2.131328
H	-0.810424	-1.70498	3.25471

H	-2.857158	-1.792167	-1.850522
H	-5.328102	-0.037558	0.19138
H	-4.712985	-1.691052	0.282145
H	-4.686515	-0.801163	-1.251902

**7-n**			
Atom	X axis (Å)	Y axis (Å)	Z axis (Å)
C	4.289771	-0.397347	-0.09389
C	3.892923	-1.711735	-0.261635
C	2.553135	-2.072234	-0.343421
C	1.590939	-1.085194	-0.257699
C	1.953116	0.270121	-0.104042
C	3.325524	0.600086	-0.01402
O	0.293707	-1.471713	-0.357376
C	-0.704154	-0.590194	0.200309
C	-0.483344	0.813447	-0.403579
C	0.933774	1.306366	-0.086991
C	-2.084667	-1.151145	-0.208975
C	-3.195845	-0.163034	0.194904
C	-2.951742	1.22842	-0.394205
C	-1.58688	1.800763	-0.017068
O	3.722614	1.866418	0.141296
O	1.187562	2.495292	0.048449
O	-1.478688	2.049721	1.374622
O	-0.542484	0.751182	-1.832651
C	-0.534309	-0.649296	1.734087
O	-0.629851	-1.973076	2.213111
O	-2.149296	-1.512086	-1.574515
C	-4.569978	-0.69804	-0.189761
H	5.334875	-0.129304	-0.021078
H	4.648231	-2.485277	-0.322571
H	2.250231	-3.103316	-0.46165
H	-2.229916	-2.082083	0.339234
H	-3.168973	-0.075215	1.283474
H	-3.037786	1.2007	-1.483626
H	-3.723767	1.917925	-0.044273
H	-1.396624	2.719888	-0.577068
H	2.922778	2.432093	0.163578
H	-2.035512	2.80036	1.589442
H	0.150031	0.167012	-2.159666
H	0.424794	-0.204643	2.016875
H	-1.309179	-0.07084	2.223035
H	0.042897	-2.503388	1.777451
H	-1.909797	-0.7385	-2.09538

H	-4.664078	-0.795084	-1.272322
H	-5.351527	-0.022096	0.162161
H	-4.744772	-1.680053	0.25481

**7-o**			
Atom	X axis (Å)	Y axis (Å)	Z axis (Å)
C	4.262201	-0.518108	-0.111862
C	3.873294	-1.828675	0.105814
C	2.536608	-2.193228	0.202776
C	1.564166	-1.217622	0.068671
C	1.92171	0.121073	-0.178546
C	3.28761	0.462944	-0.248421
O	0.276301	-1.615737	0.171605
C	-0.724619	-0.617779	0.435863
C	-0.519539	0.546269	-0.560124
C	0.8877	1.11851	-0.382739
C	-2.09018	-1.286488	0.196815
C	-3.234819	-0.256681	0.264228
C	-2.987588	0.928238	-0.671728
C	-1.635926	1.598385	-0.468671
O	3.673626	1.729658	-0.458154
O	1.113544	2.313846	-0.530921
O	-1.614347	2.295946	0.779769
O	-0.475914	0.071786	-1.89912
C	-0.639442	-0.238401	1.938181
O	0.220164	0.830474	2.252399
O	-2.027311	-1.936752	-1.069865
C	-4.586161	-0.90864	-0.016264
H	5.305966	-0.241726	-0.174091
H	4.634046	-2.591937	0.21463
H	2.242844	-3.216026	0.393754
H	-2.23917	-2.040846	0.97823
H	-3.269754	0.117804	1.288499
H	-3.050303	0.591382	-1.709041
H	-3.769783	1.676208	-0.533974
H	-1.460895	2.308371	-1.277655
H	2.867566	2.281594	-0.518509
H	-1.20369	3.150256	0.629949
H	-1.017213	-0.73268	-1.954472
H	-1.638619	-0.03887	2.32582
H	-0.260459	-1.12493	2.446856
H	-0.266157	1.628683	2.005054
H	-2.875277	-2.343531	-1.260821
H	-4.684626	-1.206008	-1.064088

H	-5.391351	-0.202146	0.188162
H	-4.741703	-1.78813	0.612035

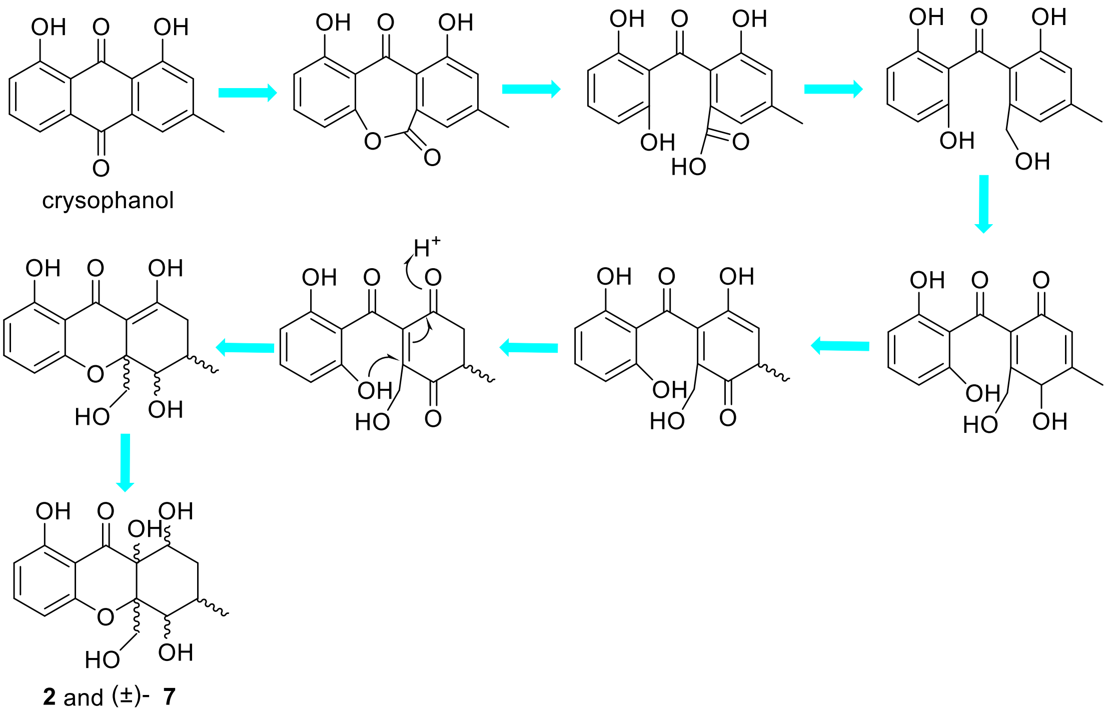

Figure S62. Plausible biogenetic pathway leading to a formation of 2 and (±)-**7**.

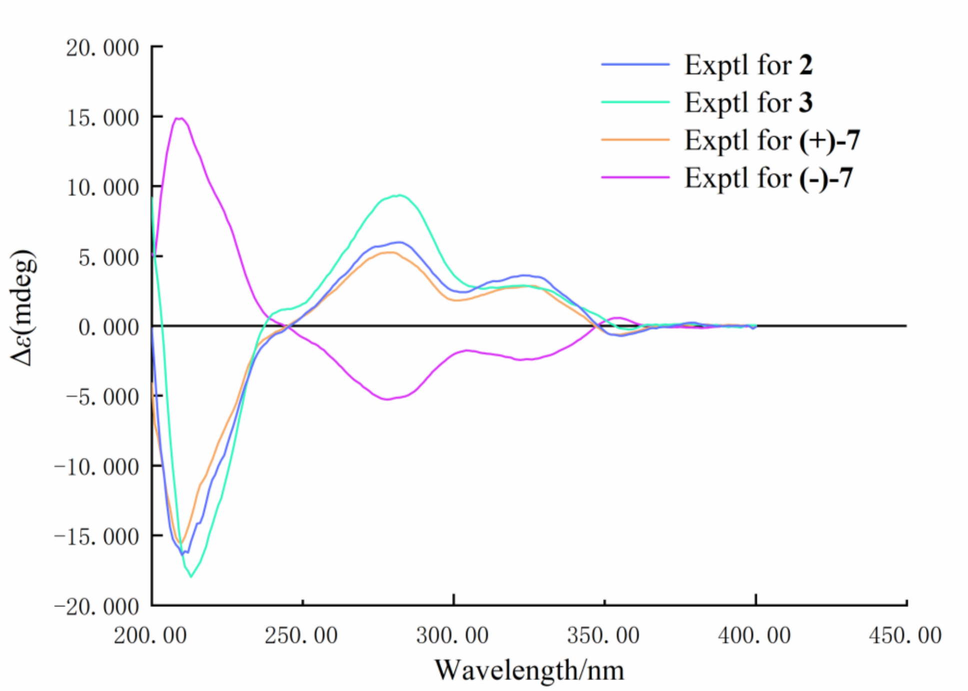

Figure S63. Experimental ECD spectra of **2**, **3**, (+) and (-)-**7** (in MeOH).
